# Effect of Meal-Timing on the Association of Unsaturated Fatty Acids with All-Cause and Cardiovascular Mortality among Adults: A Prospective Cohort Study with 10-Year Follow-Up

**DOI:** 10.3390/nu16132071

**Published:** 2024-06-28

**Authors:** Jian Gao, Chuan Li, Huan Chen, Zhi-Hao Li, Fang-Fei You, Wei-Qi Song, Wen-Fang Zhong, Pei-Liang Chen, Jin Yang, Qing-Mei Huang, Chen Mao

**Affiliations:** 1 Department of Laboratory Medicine, Microbiome Medicine Center, Zhujiang Hospital, Guangzhou 510515, China; gaojian_2022@smu.edu.cn; 2 Department of Epidemiology, School of Public Health, Southern Medical University, Guangzhou 510515, China; lichuan202105@163.com (C.L.); chenhuanoool@163.com (H.C.); zhihaoli2013@163.com (Z.-H.L.); fangfei1@smu.edu.cn (F.-F.Y.); songweiqi40@126.com (W.-Q.S.); zhongwf0613@hotmail.com (W.-F.Z.); tianlang@smu.edu.cn (P.-L.C.); 13546460199@163.com (J.Y.); qingmei71@126.com (Q.-M.H.)

**Keywords:** unsaturated fatty acid, chrono-nutrition, mortality, cardiovascular disease

## Abstract

Background: Conflicting results have been reported on the association of dietary unsaturated fatty acids (UFAs) with longevity and cardiovascular health. Most previous studies have focused only on the amount of UFAs consumed, not the timing of intake. Methods: This prospective cohort study used data from 30,136 adults aged 18 years and older. Intakes of UFAs by meal time and types were assessed by a 24-h dietary recall for two days. The covariate-adjusted survey-weighted Cox proportional hazards models were performed to evaluate the associations of dietary total unsaturated fatty acid (TUFA), polyunsaturated fatty acid (PUFA), and monounsaturated fatty acid (MUFA) intakes throughout the day and three meals with mortality. Results: During a median of 10.0 years of follow-up, 4510 total deaths occurred. All-cause mortality decreased with increasing intakes at dinner of TUFA (HR: 0.87 [0.77–0.98]), PUFA (HR: 0.81 [0.73–0.91]), and MUFA (HR: 0.88 [0.77–0.99]). With an increased intake of PUFA at dinner, CVD mortality showed a decreasing trend. However, the inverted L-shaped non-linear trend in all-cause mortality was found with increasing intake at breakfast of TUFA (HR: 1.35 [1.17–1.57], Q3 vs. Q1), PUFA (HR: 1.30 [1.13–1.50]), and MUFA (HR: 1.28 [1.13–1.45]). Meanwhile, increased breakfast intake of UFAs was associated with increased CVD and heart disease mortality. Conclusions: Meal timing influences the association of UFAs with all-cause and CVD-related mortality.

## 1. Introduction

Cardiovascular disease (CVD) is the leading cause of death and loss of healthy life in the world [1,2]. Based on 2020 data, 239.8 deaths per 100,000 people worldwide were attributed to CVD [3]. As one of Life’s Essential 8 promulgated by the American Heart Association, a healthy diet can delay the onset of chronic metabolic diseases, such as CVD, and prolong life [4]. As a major component of a healthy diet, the impact of unsaturated fatty acids (UFAs) on life has been widely studied in the fields of medicine and public health, but the long-term associations of dietary UFAs with health outcomes remain controversial [5]. Previous population-based studies have shown that dietary UPF intake is associated with a reduced risk of death and cardiovascular events [3,6,7,8]. However, clinical studies and reviews in recent years suggest that current evidence does not support the benefit of increased polyunsaturated fatty acid (PUFA) or monounsaturated fatty acid (MUFA) intake for improved cardiovascular health and reducing the risk of all-cause or CVD mortality [9,10,11].

Although the effect of dietary fatty acids is determined by the amount consumed, there is evidence that the timing of energy and macronutrient intake plays an important role in human disease and health [12]. Many physiological processes exhibit fluctuations in circadian rhythms controlled by the circadian clock, including eating behavior and lipid and carbohydrate metabolism [13]. The proper functioning of the circadian clock is essential for maintaining metabolic health [14,15]. The timing of food intake and nutrient content are important factors in the human circadian clock. Therefore, eating at inappropriate times may have an asynchronous effect on the biorhythm and lead to adverse health consequences. However, most current chrono-nutrition studies have focused on time-restricted eating (TRE) and the distribution of total energy throughout the day [16,17]. To our knowledge, no studies have investigated the associations of dietary UFAs with mortality by considering the differences in eating time. Our research may fill this gap and provide scientific evidence for promoting precision nutrition and improved dietary guidelines.

The present study assessed the relationships of total unsaturated fatty acid (TUFA), PUFA, and MUFA intakes at different meal times with all-cause and CVD-related mortality among 30,136 participants in the National Health and Nutrition Examination Survey (NHANES) 2003–2018. We hypothesized that proper and regular mealtime intake of dietary UFAs is necessary for the maintenance of metabolic homeostasis and health.

## 2. Materials and Methods

### 2.1. Study Population

The NHANES is a multistage stratified sampling study to assess information on the health and nutritional status of the noninstitutionalized civilian population in the U.S. [18]. This cohort study enrolled participants aged 18 years or older who completed at least 1 dietary recall during the 8 cycles of NHANES 2003–2018. The individuals with potentially unreliable dietary intake, those who had abnormal caloric intake (<500 or >5000 kcal/day), those who had missing values on any individual component for age, gender, race and ethnicity, and dietary information, and those with no linked mortality data were excluded from the analysis. The participants in the NHANES study provided informed consent, and the survey design received approval from the National Center for Health Statistics Ethics Review Board. As this study utilized publicly available, anonymized data from NHANES, additional review by an institutional review board was not required.

### 2.2. Dietary Assessment

In NHANES, diet information was assessed by a 24-h dietary recall for two nonconsecutive days (Supplementary Materials). Dietary nutrient and energy values were estimated using the U.S. Department of Agriculture’s Food and Nutrient Database for Dietary Studies. Based on the two 24-h dietary recall interviews, the mean values of dietary nutrient intake were calculated. We determined three meals according to the names of eating occasions given by the participants for individual foods. The energy and nutrient intakes of breakfast, lunch, and dinner were measured. We calculated the percentage of total energy (%E) of each type of UFA in both the entire day’s diet and each of the three meals [19,20].

### 2.3. Ascertainment of Mortality

The corresponding mortality information for each participant was identified through linkage to the National Mortality Index up to 31 December 2019. For this study, the primary outcome was all-cause mortality, and the secondary outcome was mortality from cardiovascular disease, heart disease, and hypertension. Cardiovascular disease mortality was defined as any death related to heart disease, cerebrovascular disease, and/or hypertension. The International Classification of Diseases (ICD)-10 was used to determine disease-specific deaths. Death from heart disease was defined as codes I00–09, I11, I13, and I20–51, and death from cerebrovascular disease was defined as codes I60–I69 according to ICD-10.

### 2.4. Covariate Assessment

Several variables were identified as potential confounders in our analysis in the following main aspects: (1) Demographic characteristics: age (years), gender (female, male), race/ethnicity (non-Hispanic white, non-Hispanic black, Mexican American, others), education level (less than 9th grade, 9–11th grade, high school, college, college graduate or above), annual household income (under USD 20,000, USD 20,000–USD 45,000, UAD 45,000–USD 75,000, USD 75,000–USD 100,000, over USD 100,000). (2) Health status: the body mass index (BMI, kg/m^2^), disease history of CVD, diabetes, hypertension, and/or high cholesterol (yes/no), family history of CVD (yes/no). (3) Lifestyle factors: moderate to vigorous activity regularly (yes/no), current smoking status (yes/no), current drinking status (yes/no), sleep duration (hour), night shift work (yes/no). (4) Dietary factors: daily energy intake (kcal/d), alternative healthy eating index (AHEI), meal skipping (at breakfast/lunch/dinner/none), dietary supplements taken (yes/no).

### 2.5. Stratification and Sensitivity Analyses

We applied stratification analyses for the associations of UFA intake in the whole day and three meals with mortality according to gender (female, male) and age group (age ≥ 60 years, age < 60 years).

We conducted four sensitivity analyses to test the robustness of our findings. First, to investigate the effect of UFA intake at breakfast relative to intakes at the other two main meals, we analyzed the difference between total consumption at lunch and dinner and breakfast (Δ = UFAs at lunch + dinner − breakfast, %E) in relation to the risk of all causes of death and CVD-related mortality. Second, in order to avoid factors that might affect longevity due to participants’ severe illness and to observe the long-lasting effects of dietary habits on health, we excluded participants who had been followed for less than 2 years. Third, in order to exclude possible effects of cardiovascular health status on the relationship between the timing of dietary UFA intake and CVD-related mortality, we adjusted not only for CVD disease history in the CPH model but also excluded participants who had CVD at baseline from sensitivity analyses. Last, because the meal timing of participants working night shifts may not be consistent with usual habits and considering that their reversed circadian rhythms may affect the health effects of dietary intake rhythms, we excluded these participants and reanalyzed.

### 2.6. Statistical Analysis

All analyses were performed with the incorporation of sample weights, stratification, and clusters to account for the complex survey design. The baseline characteristics of participants were described according to TUFA quintiles of all-day, breakfast, and dinner intakes. Descriptive analyses were presented as weighted mean (95% CI) for continuous variables and unweighted number of participants (weighted percentage) for categorical variables.

Survey-weighted Cox proportional hazards (CPH) models were developed to evaluate the association of UFA intake throughout the day and three meals with mortality. The percentages of energy from TUFA, PUFA, and MUFA intakes were categorized into quintiles and processed by standard deviation standardization method, respectively. All models were tested for the proportional hazards hypothesis using the Kaplan–Meier method. We also estimated the dose–response relationship between UFA intake and mortality risk with weighted restricted cubic spline analysis (RCS) using 4 knots placed at the 5th, 35th, 65th, and 95th sample-weighted percentiles [21]. The reference value was set to the median of the first quintile. The values outside the 5th and 95th percentiles were excluded from the analysis. The analysis of variance (ANOVA) was used to examine the linear or non-linear relationship of the spline.

The differences between UFA intake at dinner and breakfast (Δ = UFAs at dinner–breakfast, %E) were categorized into quintiles. Participants were divided into four groups based on the median consumption of UFAs at breakfast and dinner, including Lower intake at Breakfast and Lower intake at Dinner (BL-DL), Lower intake at Breakfast and Higher intake at Dinner (BL-DH), Higher intake at Breakfast and Lower intake at Dinner (BH-DL), and Higher intake at Breakfast and Higher intake at Dinner (BH-DH). We further built the predicted isocaloric substitution models to evaluate the extent to which a theoretical shift of 10% energy from breakfast to dinner would impact mortality by holding total energy intake constant.

Hazard ratios (HRs) and 95% CIs adjusted for multiple covariates were controlled in all weighted CPH and RCS models. All statistical analyses were conducted by R 4.4.0, and two-sided *p* < 0.05 was considered to be statistically significant.

## 3. Results

### 3.1. Participant Characteristics

**Figure 1:**
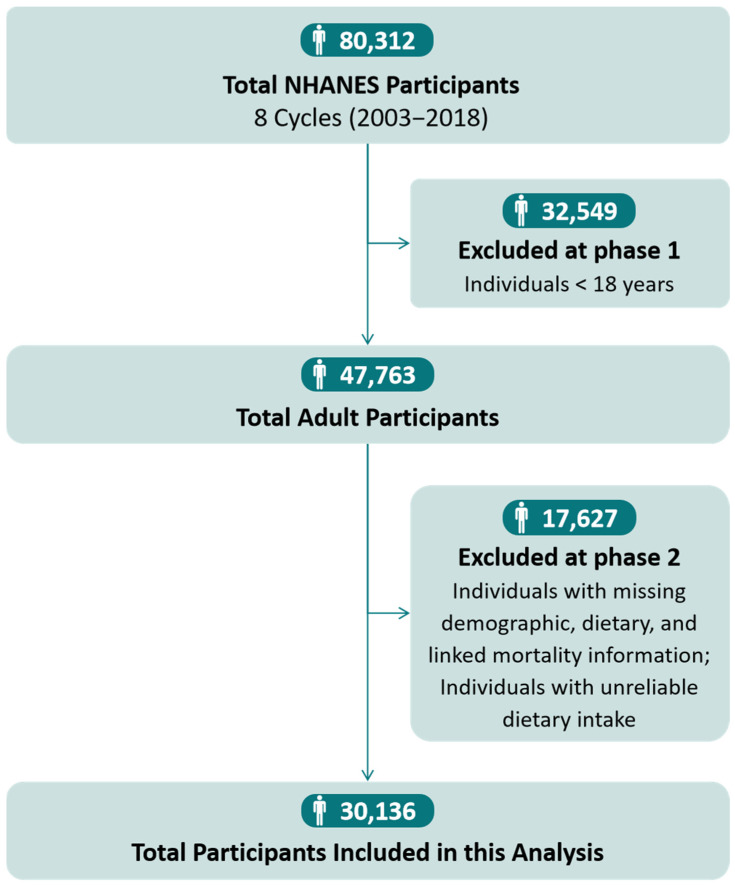
Flow chart of the study population.

A total of 30,136 US adults (mean [standard error, SE] age, 46.33 [0.26] years; 15,303 [51.6%] female) were included in the present analysis (Figure 1). Table 1 and Supplementary Table S1 show the baseline characteristics of participants according to TUFA quintiles of all-day, breakfast, and dinner intakes. Participants with a higher TUFA intake were more likely to be older and have comorbidity conditions (CVD, diabetes, hypertension, and high cholesterol). With increasing TUFA daily intake, the participants tended to be non-Hispanic white, current smokers and drinkers, and traditional schedule workers and had higher levels of education, income, dietary energy, and supplements intake but lower AHEI scores. Similar results were observed for the participants who had higher TUFA intake at dinner. In contrast, participants with a higher TUFA intake at breakfast had lower proportions of being white, smokers, and drinkers and had lower levels of education, income, dietary energy, and supplement intake. They also tended to work shifts and exercise regularly.

### 3.2. Effect of Meal Timing on Associations of UFAs with All-Cause Mortality

**Figure 2:**
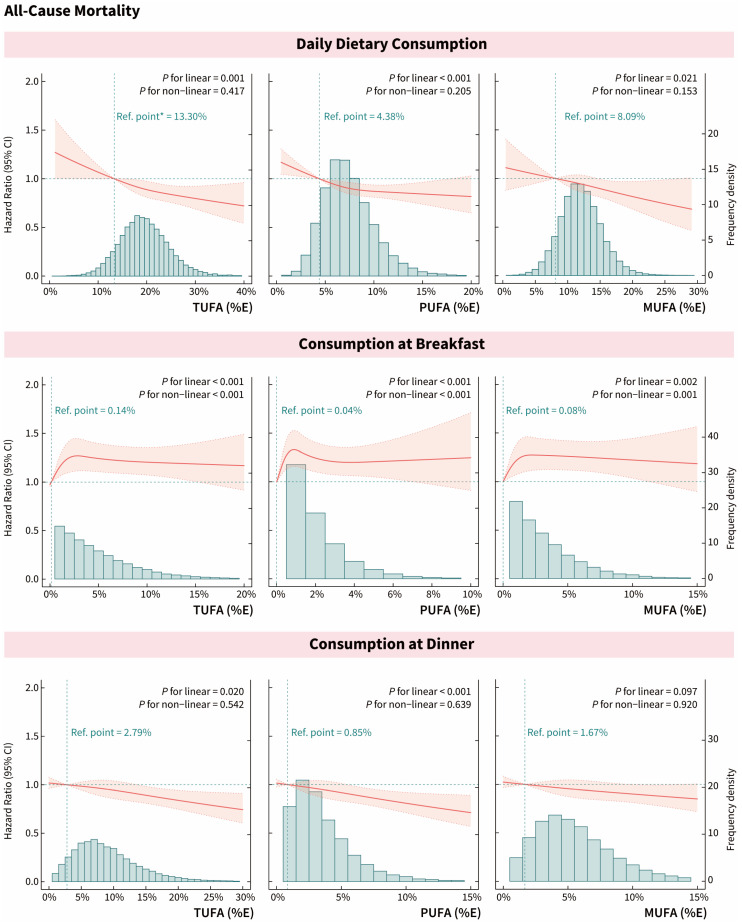
Dose–response associations between dietary UFA intake and the risk of all-cause mortality. Associations were examined by survey-weighted Cox regression models based on restricted cubic splines. All models were adjusted for age, gender, race/ethnicity, education level, annual household income, BMI, disease history of CVD, diabetes, hypertension, and/or high cholesterol, family history of CVD, physical activity regularly, current smoking and drinking status, sleep duration, night shift work, daily energy intake, AHEI, meal skipping, and dietary supplements taken. The hazard ratios are indicated by solid red lines, and 95% CIs are indicated by shaded areas. The distribution of intakes in the population is shown by the green bar charts. * The reference point (the green vertical dashed line) was set to the median of the first quintile. The green horizontal dashed line meant Hazard Ratio = 1.00.

During a median of 10.0 years of follow-up, 4510 total deaths occurred, including 1801 deaths from CVD, 1169 deaths from heart disease, and 702 deaths from hypertension. After adjustment for multivariate variables, daily intakes of TUFA, PUFA, and MUFA were all significantly associated with a reduced risk of all-cause mortality (Figure 2 and Supplementary Tables S2–S4). Similarly, all-cause mortality was significantly reduced among participants in the highest quintile of UFA intake at dinner compared with participants in the lower quintile (TUFA_dinner_: HR [95% CI] = 0.87 [0.77–0.98]; PUFA_dinner_: HR [95% CI] = 0.81 [0.73–0.91]; MUFA_dinner_: HR [95% CI] = 0.88 [0.77–0.99]). And the intakes of UFAs at lunch were not associated with all-cause mortality. However, with increasing UFAs in breakfast, the risk of all-cause mortality increased significantly and then remained relatively stable (Q3 versus Q1: TUFA_breakfast_: HR [95% CI] = 1.35 [1.17–1.57], *p*_non-linear_ < 0.001; PUFA_breakfast_: HR [95% CI] = 1.30 [1.13–1.50], *p*_on-linear_ < 0.001; MUFA_breakfast_: HR [95% CI] = 1.28 [1.13–1.45], *p*_non-linear_ = 0.001). All models generally satisfied the prerequisite of proportional risk assumption.

### 3.3. Effect of Meal Timing on Associations of UFAs with CVD-Related Mortality

**Figure 3:**
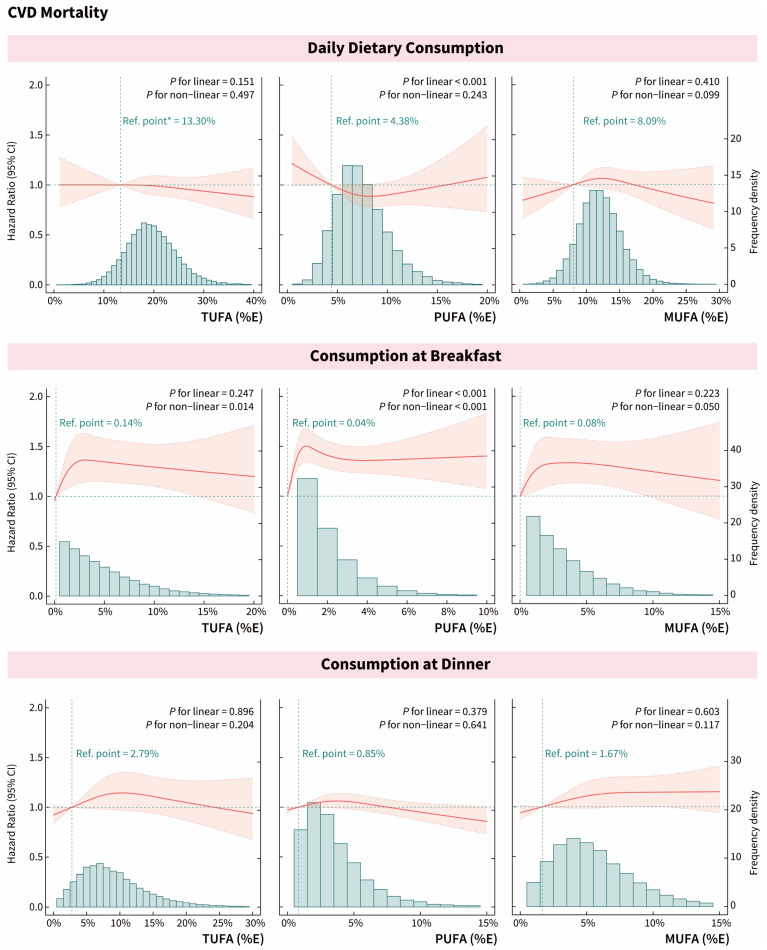
Dose–response associations between dietary UFA intake and the risk of CVD mortality. Associations were examined by survey-weighted Cox regression models based on restricted cubic splines. All models were adjusted for age, gender, race/ethnicity, education level, annual household income, BMI, disease history of CVD, diabetes, hypertension, and/or high cholesterol, family history of CVD, physical activity regularly, current smoking and drinking status, sleep duration, night shift work, daily energy intake, AHEI, meal skipping, and dietary supplements taken. The hazard ratios are indicated by solid red lines, and 95% CIs are indicated by shaded areas. The distribution of intakes in the population is shown by the green bar charts. * The reference point (the green vertical dashed line) was set to the median of the first quintile. The green horizontal dashed line meant Hazard Ratio = 1.00.

The relationships between daily and three-meal intakes of UFAs and mortality from CVD, heart disease, and hypertension mortality are shown in Figure 3 and Supplementary Tables S2–S4. We found the inverted L-shaped non-linear trend in CVD and heart disease mortality with increasing TUFA intake at breakfast (Q3 versus Q1 of TUFA_breakfast_: HR [95% CI] of CVD = 1.33 [1.07–1.65], *p*_non-linear_ = 0.014; HR [95% CI] of heart disease = 1.41 [1.10–1.80], *p*_non-linear_ = 0.099). Meanwhile, the daily and another meal intakes of TUFA were not statistically significantly associated with CVD-related mortality. Similar results were found in the relationship between daily and three-meal intakes of MUFA and CVD-related mortality (Q3 versus Q1 of MUFA_breakfast_: HR [95% CI] of CVD = 1.24 [1.00–1.54], *p*_non-linear_ = 0.050; HR [95% CI] of heart disease = 1.30 [1.01–1.49], *p*_non-linear_ = 0.216).

For dietary PUFA, with each additional SD of all-day and dinner intakes, the risk of CVD mortality decreased by 7% and 6%, respectively (PUFA_all-day_: HR [95% CI] = 0.93 [0.87–0.99]; PUFA_dinner_: HR [95% CI] = 0.94 [0.89–0.99]). However, we also observed that PUFA consumption at breakfast was a risk factor for CVD mortality (Q3 versus Q1 of PUFA_breakfast_: HR [95% CI] = 1.38 [1.12–1.70], *p*_non-linear_ < 0.001). Similarly, heart disease mortality was slightly reduced with increasing daily intake of PUFA but partially increased with increasing PUFA intake at breakfast (Q3 versus Q1: HR [95% CI] = 1.43 [1.09–1.88], *p*_non-linear_ = 0.111). Meanwhile, all-day and three-meal intakes of PUFA were not associated with hypertension mortality.

### 3.4. Differences in UFA Intakes between Breakfast and Dinner and Mortality

**Figure 4:**
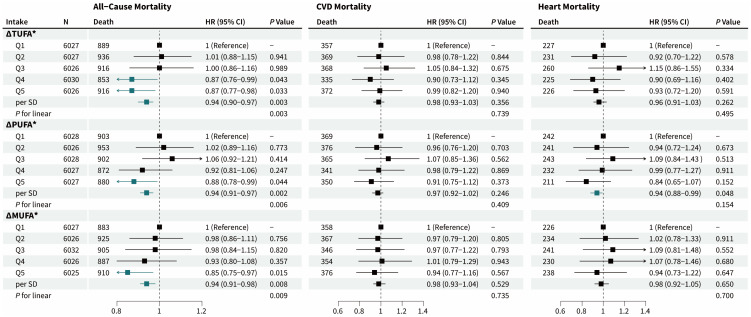
Associations of the difference in UFA intakes between dinner and breakfast with the risk of mortality. The differences in TUFA, PUFA, and MUFA intakes between dinner and breakfast were categorized into quintiles. Risk estimates for sample weight were adjusted for age, gender, race/ethnicity, education level, annual household income, BMI, disease history of CVD, diabetes, hypertension, and/or high cholesterol, family history of CVD, physical activity regularly, current smoking and drinking status, sleep duration, night shift work, daily energy intake, AHEI, meal skipping, and dietary supplements taken. The green squares and arrows meant *p*-value < 0.05. SD, standard deviation. * Δ, UFA intake (%E) at dinner minus at breakfast.

Figure 4 shows the association of ΔUFAs (Δ = dinner − breakfast) with all-cause and CVD-related mortality. As indicated by HR and 95% CI, the participants in the highest quintile of ΔUFAs had a lower risk of all-cause mortality compared to those in the lowest (ΔTUFA: 0.87 [0.77–0.98]; ΔPUFA: 0.88 [0.78–0.99]; ΔMUFA: 0.85 [0.75–0.97]). In addition, each SD increase in ΔPUFA was associated with a 6% lower risk of death from heart disease (HR [95% CI] = 0.94 [0.88–0.99]) (Supplementary Table S5 and Supplementary Figure S1). 

**Figure 5:**
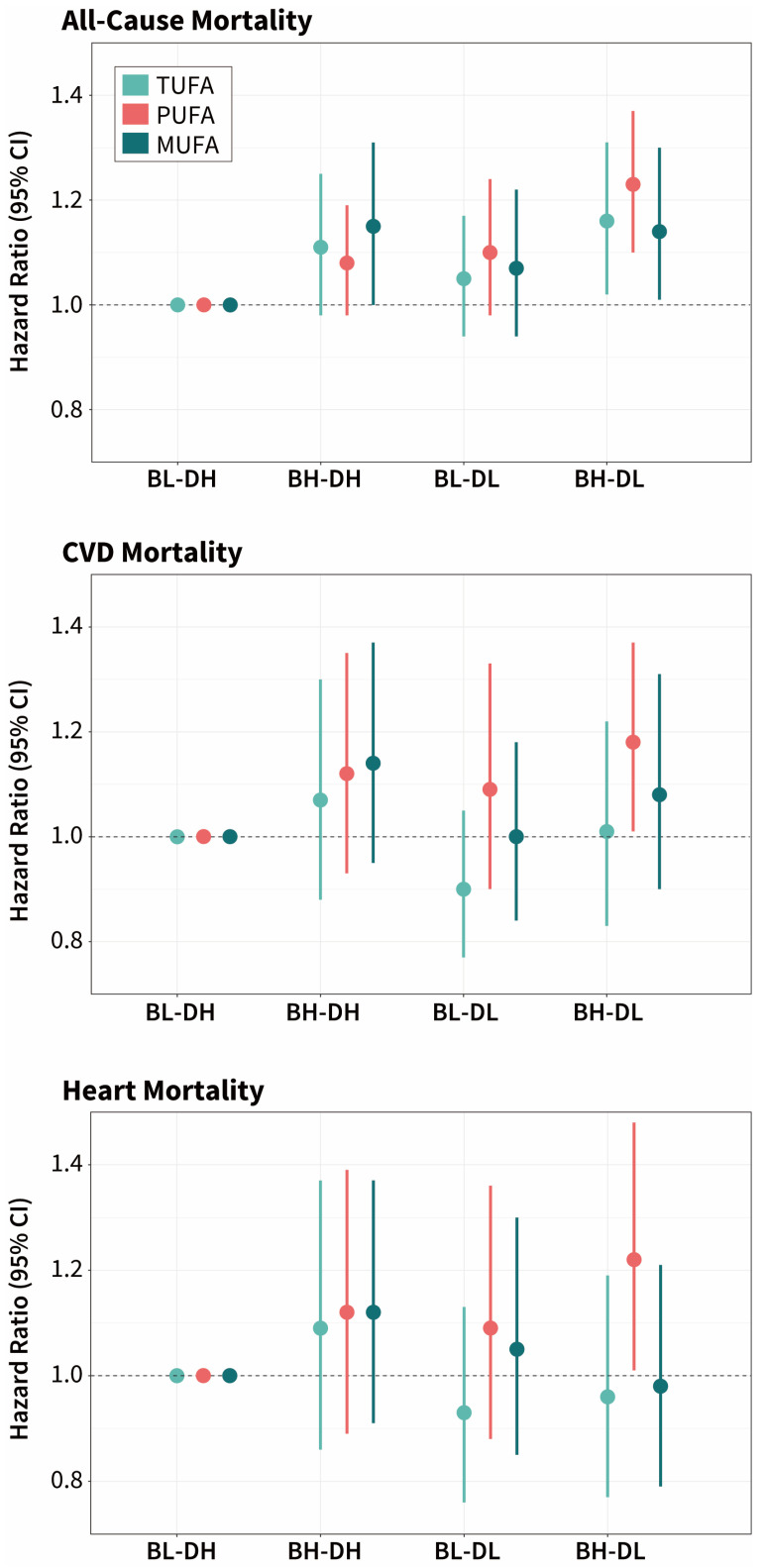
Associations of the different intake levels at breakfast and dinner of UFAs with the risk of mortality. Light green dots and lines represent the HR (95% CI) of TUFA; red dots and lines represent the HR (95% CI) of PUFA; dark green dots and lines represent the HR (95% CI) of MUFA. BL-DL represents the participants with Lower intake at Breakfast and Lower intake at Dinner; BH-DL represents the participants with Higher intake at Breakfast and Lower intake at Dinner; BH-DH represents the participants with Higher intake at Breakfast and Higher intake at Dinner; BL-DH, as the reference group, represents the participants with Lower intake at Breakfast and Higher intake at Dinner. Risk estimates for sample weight were adjusted for multivariate.

At the same time, taking participants with a lower intake of UFAs at breakfast and a higher intake at dinner (BL-DH) as a reference, we found that participants who had a higher intake of UFAs at breakfast and a lower intake at dinner (BH-DL) had a significantly increased risk of all-cause mortality (TUFA: HR [95% CI] = 1.16 [1.02–1.31]; PUFA: HR [95% CI] = 1.23 [1.10–1.37]; MUFA: HR [95% CI] = 1.14 [1.01–1.30]) (Figure 5). In addition, participants with PUFA intake in the BH-DL group had higher CVD and heart disease mortality compared with the BL-DH group (HR [95% CI] of CVD = 1.18 [1.01–1.37], HR [95% CI] of heart disease = 1.22 [1.01–1.48]) (Supplementary Table S6 and Supplementary Figure S2).

Further, we used an isocaloric substitution model to predict changes in mortality by replacing UFA intake at breakfast with that at dinner (Supplementary Table S7). The results showed that when TUFA, PUFA, and MUFA intakes were substituted for 10% of the energy in the breakfast to dinner, the risks of all-cause mortality were reduced by 16%, 28%, and 24%, respectively (TUFA: HR [95% CI] = 0.84 [0.74–0.94]; PUFA: HR [95% CI] = 0.72 [0.54–0.98]; MUFA: HR [95% CI] = 0.76 [0.64–0.91]).

### 3.5. Subgroup and Sensitivity Analyses

Supplementary Tables S8 and S9 show results stratified by gender (female, male) and age group (age ≥ 60 years, age < 60 years). In females, daily TUFA intake was significantly negatively associated with the risk of all-cause mortality. However, with increasing UFAs in breakfast, the risk of all-cause mortality increased significantly and then remained relatively stable (Q3 versus Q1: TUFA_breakfast_: 1.38 [1.13–1.70]; PUFA_breakfast_: 1.37 [1.11–1.70]; MUFA_breakfast_: 1.36 [1.11–1.67]). However, consumption of UFAs at dinner did not affect the risk of all-cause mortality. At the same time, we also found that breakfast intake of UFAs was associated with an increased risk of CVD-related mortality, whereas dinner intake of UFAs had no harmful effect. Similar results were also found in males, middle-aged adults (age < 60 years), and older adults (age ≥ 60 years). There were no substantial differences in results between subgroups. Differences in meal timing on UFAs affected their association with the risk of all-cause and CVD-related mortality and were similar to the primary analysis. In sensitivity analyses, we demonstrated that the more UFAs consumed at breakfast relative to the other two main meals, the higher the risk of all-cause and CVD-related mortality. In addition, the associations were even more pronounced when we further excluded participants with less than 2 years of follow-up, a disease history of CVD, and regular night shifts at baseline (Supplementary Tables S10–S13).

## 4. Discussion

In a large nationally representative sample of US adults, we found significant negative associations between daily intake of UFAs (TUFA, PUFA, MUFA) and all-cause mortality. Additionally, PUFA all-day intake was associated with a reduced risk of CVD and heart disease mortality. However, when we analyzed UFA intake by meal time, the effects of UFA intake at breakfast and dinner on mortality were almost the opposite. Similar to all-day intake, UFA intake at dinner was negatively associated with all-cause mortality, whereas UFA intake at breakfast could increase the risk of all-cause and CVD-related mortality. Moreover, the greater the difference between UFA intake at dinner and breakfast, the higher the risk of mortality. We further examined the stratification results by age and gender [22] and found no differences from the main study findings.

In order to assess the intake of UFAs relative to other macronutrients, including saturated fatty acids, trans fatty acids, protein, carbohydrates, etc., we used the proportion of energy provided by UFAs in total energy throughout the day as the observation index in this study. The results of this study were verified to be stable across age and gender. To avoid the risk of life expectancy being affected by factors such as the participant’s serious illness and to observe the lasting effects of dietary habits on health, we excluded participants with less than 2 years of follow-up and performed sensitivity analyses. In addition, to exclude the effect of underlying cardiovascular health on the relationship between the timing of meal intake and CVD-related mortality, we not only adjusted for disease history of CVD in the CPH model but also excluded participants who had CVD at baseline in sensitivity analyses. The results did not change significantly, suggesting that the effect of meal timing was independent of the participants’ cardiovascular health. Since the timing of UFA intake is also one of the biorhythms, its effects on health are influenced by other circadian rhythms, so we controlled for other confounding factors in the CPH model as much as possible, such as sleep duration, night shift work, and meal skipping. And because the meal times of participants working night shifts may not be consistent with the usual three meals, and their reversed circadian rhythms may affect the health effects of meal intake rhythms, we excluded these participants and reanalyzed. At the same time, we found no statistically significant association between UFA intake at lunch and mortality in our results and hypothesized that most of the fatty acid metabolites or related gene expression levels in circulation and tissues at noon did not reach the peak or trough of the oscillation cycle. Therefore, the effect of UFA intake at lunch on body health may not be significant. And to examine the effect of UFA intake at breakfast on the risk of death relative to the intakes at the other two main meals, we confirmed in sensitivity analyses that the greater the difference between the total amount of lunch and dinner and breakfast (Δ = UFAs at [lunch + dinner] − breakfast, %E), the lower the risk of all-cause and CVD-related death.

As in previous prospective studies, our results show that dietary PUFA intake is inversely associated with all-cause mortality [23,24]. Similarly, a prospective cohort study using PUFA in serum cholesterol esters as a circulating biomarker of dietary intake observed a significant inverse association between PUFA and all-cause mortality [25,26]. Consistent with our findings, most previous prospective cohort studies have shown that PUFA intake reduces the risk of CVD-related events and death [5,26,27,28,29,30,31,32,33,34]. PUFAs are presumed to have anti-inflammatory and antithrombotic properties, lower plasma triglycerides and low-density lipoprotein cholesterol, improve vasomotor and endothelial function, and reduce the risk of hypertension [27,29,35]. And this effect may be responsible for the protective effect on CVD mortality [36]. However, several recent review studies have concluded, to the contrary, that increasing PUFA intake may slightly reduce the risk of cardiovascular events but has little or no effect on all-cause or CVD mortality [10]. In addition, the results of several RCTs have raised concerns about high doses of UFA supplementation and an increased risk of new atrial fibrillation (AF) and bleeding events [37,38,39,40].

Numerous studies have explored the health effects of dietary PUFA, but the conclusions of these studies are not uniform. Although the effect of dietary fatty acids is determined by consumption, the meal time of dietary fatty acids, as another important factor affecting the body’s health, is often ignored. Several possible mechanisms could be involved in the associations between the time of UFA intake and mortality. Feeding time and dietary nutrients are two key environmental factors affecting the circadian rhythm of body fat metabolism [41]. The main function of the circadian rhythm is to ensure that the daily patterns of eating behavior and energy metabolism are aligned with the solar day length and food supply, thereby maintaining homeostasis. The circadian system coordinates lipid synthesis, fatty acid oxidation, emulsification, and absorption of dietary lipids with the daily cycle of feeding behavior. This may optimize the body’s energy resources and promote physiological adaptation to environmental stress. However, when food consumption is out of sync with the circadian clock, it can lead to dysregulation of energy and lipid metabolism, causing metabolic diseases. It is suggested that eating at an unfavorable circadian rhythm phase may have a significant negative impact on metabolic health.

In peripheral tissues, hundreds of genes involved in lipid biosynthesis and fatty acid oxidation are rhythmically activated and repressed by clock proteins, providing a direct mechanism for the circadian regulation of lipids [42]. In the early days of the active phase (breakfast time), food consumption leads to an increase in blood glucose, which leads to an increase in glucose absorption and glycogen synthesis. Late in the active phase (dinner time), lipids and proteins are preferentially metabolized [43,44], and lipolysis in adipose tissue is increased [45,46]. During inactivity (sleep time), glutamine synthase and autophagy pathways are upregulated in skeletal and cardiac muscle tissue and the liver [47]. Therefore, when the dietary UFAs are ingested at dinner, they can be more efficiently metabolized and utilized by the body; that is, they conform to the biological clock law of human lipid metabolism. At the same time, the studies using metabolomics and lipidomics platforms have reconfirmed that intake of UFAs at dinner time, rather than at breakfast time, is more in line with the biorhythm of plasma lipid levels [48,49].

On the other hand, key events in the pathogenesis of atherosclerotic cardiovascular disease also exhibit a remarkably consistent circadian rhythm and peak at the rest–activity transition time (morning). This pathophysiological chain may increase the chance of inflammation with increased exposure to potential risk factors. There is growing evidence that targeting the circadian rhythms of key lipids may enhance the prevention and control of CVD-related diseases. Consistent with our findings, a prospective cohort study of 27,911 US adults also showed that UFA intake at dinner was associated with a lower CVD prevalence [50].

### Strengths and Limitations

In this study, key strengths include the use of a nationally representative cohort study based on a probability-based sampling survey design, which provides a comprehensive and authoritative detailed assessment of dietary intake, lifestyle factors, and health status. In addition, we analyzed and controlled several important covariates that may be associated with dietary fat intake and circadian rhythms. Finally, this is the first study to address the impact of meal timing on the relationship between dietary UFA intake and mortality. This study provides a scientific basis for the guidance and improvement of dietary guidelines and individual precision nutrition. However, this study has several limitations. First, we used questionnaires to obtain information about the health status and dietary intake of subjects. Although there may be recall bias and survey error, it is still widely used in observational studies and can reflect the long-term health and dietary information of respondents to some extent. Second, we controlled for a range of potential confounders, but the study was observational in nature, and most of the variables in this study were self-reported by subjects, so we could not rule out other unmeasured confounders. Therefore, more intervention studies with higher levels of evidence and more detailed chrono-nutrition analyses are needed in the future to further verify the relationship between the meal time of dietary UFAs and body health and to explore the potential mechanism using animal experiments. Our study provides a nutritional epidemiological basis for promoting the protective effect of precision nutrition on health and improving the development of dietary guidelines.

## 5. Conclusions

In conclusion, in a nationally representative United States sample, meal timing influenced the association of TUFA, PUFA, and MUFA intakes with the risk of all-cause and CVD-related mortality. These findings suggest that it is crucial to emphasize the meal timing and types of UFAs when investigating the relationship between diet and health.

## Figures and Tables

**Table 1:** Baseline characteristics of participants according to quintile categories of total unsaturated fatty acid intake *.

Characteristics †	TUFA Daily Intake		TUFA Intake at Breakfast		TUFA Intake at Dinner	
	Quintile 1	Quintile 5	Quintile 1	Quintile 5	Quintile 1	Quintile 5
Participants, n	6027	6027	6028	6026	6027	6027
Mean age, year	45.12 (44.37–45.87)	48.58 (47.80–49.35)	42.96 (42.12–43.80)	46.62 (45.81–47.42)	45.22 (44.54–45.91)	47.74 (46.93–48.55)
Female sex, n (%)	3063 (51.7)	3052 (51.5)	2940 (49.1)	2851 (48.1)	3063 (51.7)	3052 (51.5)
Race/Ethnics, n (%):						
Mexican American	1518 (13.4)	688 (5.5)	648 (5.6)	1562 (15.5)	1518 (13.4)	688 (5.5)
Non-Hispanic white	2040 (58.0)	3153 (74.2)	3103 (73.3)	1920 (55.0)	2040 (58.0)	3153 (74.2)
Non-Hispanic black	1160 (12.1)	1487 (12.2)	1386 (11.0)	1417 (14.7)	1160 (12.1)	1487 (12.2)
Current smoking, n (%)	1340 (24.6)	1545 (27.9)	1845 (30.7)	1453 (26.6)	1340 (24.6)	1545 (27.9)
Current drinking, n (%)	3595 (66.0)	3900 (71.2)	3961 (72.8)	3774 (69.1)	3595 (66.0)	3900 (71.2)
Education level, n (%):						
Less than 9th grade	1036 (9.4)	480 (4.5)	346 (3.2)	1046 (10.2)	1036 (9.4)	480 (4.5)
9–11th grade	993 (13.3)	891 (12.3)	823 (10.3)	1070 (14.8)	993 (13.3)	891 (12.3)
High school	1452 (24.1)	1635 (26.0)	1660 (26.1)	1540 (26.1)	1452 (24.1)	1635 (26.0)
College	1458 (29.1)	1832 (32.6)	1896 (33.5)	1525 (29.9)	1459 (29.1)	1832 (32.6)
College graduate or above	1081 (24.0)	1181 (24.5)	1298 (26.9)	840 (19.0)	1081 (24.0)	1181 (24.5)
Annual household income, n (%):						
Under USD 20,000	1520 (18.5)	1238 (14.4)	1261 (14.6)	1512 (19.9)	1520 (18.5)	1238 (14.4)
USD 20,000 to USD 45,000	2103 (31.6)	1966 (29.0)	1898 (27.7)	2135 (32.1)	2103 (31.6)	1966 (29.0)
USD 45,000 to USD 75,000	958 (19.3)	1168 (21.4)	1082 (20.3)	1075 (21.0)	958 (19.3)	1168 (21.4)
USD 75,000 to USD 100,000	560 (12.0)	826 (17.9)	803 (17.6)	562 (12.5)	560 (12.0)	826 (17.9)
Over USD 100,000	561 (14.4)	616 (14.4)	726 (16.5)	439 (10.5)	561 (14.4)	616 (14.4)
BMI, kg/m^2^	28.12 (27.84–28.40)	29.38 (29.07–29.69)	28.29 (28.03–28.55)	29.24 (28.93–29.55)	28.27 (28.02–28.52)	29.19 (28.93–29.45)
Exercised regularly, n (%)	3143 (44.3)	2945 (44.9)	2693 (40.5)	3298 (48.0)	3143 (44.3)	2945 (44.9)
Dietary supplement use, n (%)	2559 (46.9)	2749 (49.2)	2416 (45.3)	2428 (43.7)	2559 (46.9)	2749 (49.2)
Duration of sleep, hour	6.92 (6.87–6.98)	6.93 (6.88–6.98)	6.90 (6.85–6.94)	6.92 (6.87–6.96)	6.92 (6.87–6.97)	6.91 (6.86–6.95)
Regular night shift work, n (%)	55 (0.9)	32 (0.5)	12 (0.2)	84 (1.3)	55 (0.9)	32 (0.5)
Family history of CVD, n (%)	1680 (32.6)	1632 (31.2)	1840 (34.5)	1550 (30.9)	1680 (32.6)	1632 (31.2)
Ever had CVD, n (%)	585 (8.1)	707 (9.3)	521 (7.1)	679 (9.6)	585 (8.1)	707 (9.3)
Ever had diabetes, n (%)	719 (9.0)	723 (9.5)	437 (5.4)	900 (12.0)	719 (9.0)	723 (9.5)
Ever had hypertension, n (%)	1976 (29.9)	2233 (34.6)	1761 (27.6)	2177 (33.0)	1976 (29.9)	2233 (34.6)
Ever had high cholesterol, n (%)	1735 (28.2)	1973 (34.3)	1554 (27.4)	1901 (32.3)	1735 (28.2)	1973 (34.3)
Dietary intake						
Total energy, kcal/d	1890.05 (1861.54–1918.56)	2180.49 (2151.34–2209.63)	2076.91 (2049.04–2104.79)	2054.81 (2028.29–2081.33)	2047.38 (2018.75–2076.02)	2069.65 (2037.22–2102.08)
Protein, g/d	73.53 (72.19–74.86)	86.23 (84.8–87.66)	80.48 (79–81.97)	81.67 (80.4–82.94)	79.61 (78.07–81.15)	81.03 (79.53–82.52)
Carbohydrate, g/d	264.7 (260.44–268.97)	223.9 (220.67–227.13)	254.33 (250.7–257.96)	234.92 (231.32–238.52)	261.92 (257.93–265.92)	232.21 (229.18–235.23)
Total fat, g/d	50.77 (49.84–51.71)	104.02 (102.47–105.57)	76.26 (74.94–77.58)	84.04 (82.6–85.47)	69.99 (68.73–71.25)	87.76 (85.97–89.55)
TUFA, g/d	27.24 (26.76–27.71)	65.41 (64.39–66.43)	44.34 (43.54–45.15)	50.09 (49.23–50.96)	40.7 (39.93–41.47)	52.77 (51.64–53.89)
PUFA, g/d	9.88 (9.71–10.05)	26.3 (25.83–26.76)	16.85 (16.52–17.18)	18.9 (18.53–19.28)	15.46 (15.11–15.81)	20.75 (20.24–21.26)
MUFA, g/d	17.35 (17.01–17.7)	39.11 (38.49–39.74)	27.49 (26.97–28.02)	31.19 (30.63–31.75)	25.24 (24.77–25.71)	32.02 (31.35–32.69)
SFA, g/d	18.48 (18.05–18.91)	30.32 (29.82–30.83)	25.17 (24.69–25.65)	26.7 (26.17–27.23)	23.04 (22.55–23.53)	27.61 (27.01–28.21)
AHEI, score	52.87 (52.35–53.4)	48.85 (48.42–49.28)	47.97 (47.55–48.4)	48.41 (48.03–48.8)	51.58 (51.17–51.99)	48.19 (47.73–48.65)
Breakfast skipping, n (%)	237 (4.1)	450 (6.9)	1677 (24.2)	0	237 (4.1)	450 (6.9)
Lunch skipping, n (%)	423 (5.5)	1105 (15.2)	363 (5.2)	1322 (18.4)	423 (5.5)	1105 (15.2)
Dinner skipping, n (%)	784 (9.9)	0	78 (1.0)	287 (3.5)	784 (9.9)	0

Abbreviations: BMI, body mass index (calculated as weight in kilograms divided by height in meters squared); CVD, cardiovascular disease; TUFA, total unsaturated fatty acid; PUFA, polyunsaturated fatty acid; MUFA, monounsaturated fatty acid; SFA, saturated fatty acid; AHEI, alternative healthy eating index. * Complete baseline characteristics of participants is presented in Supplementary Table S1. † Continuous variables are presented as weighted mean (95% CI), and categorical variables are presented as unweighted number of participants (weighted percentage).

## Data Availability

NHANES data are publicly available from the CDC website at https://www.cdc.gov/nchs/nhanes/ (accessed on 13 October 2023).

## Supplementary Materials

The following supporting information can be downloaded at: https://www.mdpi.com/article/10.3390/nu16132071/s1, Supplementary Table S1: Baseline characteristics of participants according to quintile categories of total unsaturated fatty acid intake; Supplementary Table S2: Adjusted hazard ratios for mortalities across quintiles of dietary TUFA intake in all-day and three meals; Supplementary Table S3: Adjusted hazard ratios for mortalities across quintiles of dietary PUFA intake in all-day and three meals; Supplementary Table S4: Adjusted hazard ratios for mortalities across quintiles of dietary MUFA intake in all-day and three meals; Supplementary Table S5: Association of the difference between unsaturated fat intakes of dinner and breakfast (%en) with mortality; Supplementary Table S6: Association of the mortality with the difference level between unsaturated fat intakes (%E) at dinner and breakfast; Supplementary Table S7: Isocaloric substitution model of the risk of mortality via replacing UFA intake at breakfast with that at dinner; Supplementary Table S8: Adjusted hazard ratios for mortalities stratified by gender across quintiles of dietary UFA intake in all-day and three meals; Supplementary Table S9: Adjusted hazard ratios for mortalities stratified by age group across quintiles of dietary UFA intake in all-day and three meals; Supplementary Table S10: Adjusted hazard ratios for mortalities across quintiles of dietary UFA intake in all-day and three meals, excluding participants with follow-up less than 2 years; Supplementary Table S11: Adjusted hazard ratios for mortalities across quintiles of dietary UFA intake in all-day and three meals, excluding participants who self-reported CVD; Supplementary Table S12: Adjusted hazard ratios for mortalities across quintiles of dietary UFA intake in all-day and three meals, excluding participants with regular night shifts; Supplementary Table S13: Adjusted hazard ratios for mortalities across quintiles of the difference between total consumption at lunch and dinner and breakfast; Supplementary Figure S1: Associations of the difference in UFA intakes between dinner and breakfast with the risk of hypertension mortality; Supplementary Figure S2: Associations of the difference in intake levels at breakfast and dinner of UFAs with the risk of hypertension mortality.
