# Supplementary material for: Effect of Meal-Timing on the Association of Unsaturated Fatty Acids with All-Cause and Cardiovascular Mortality among Adults: A Prospective Cohort Study with 10-Year Follow-Up
Source: Nutrients. 2024 Jun 28;16(13):2071. doi: 10.3390/nu16132071 (PMC11242975; doi:10.3390/nu16132071)

Supplementary Materials

Supplementary Table S1. Baseline characteristics of participants according to quintile categories of total unsaturated fatty acids intake.

Supplementary Table S2. Adjusted hazard ratios for mortalities across quintiles of dietary TUFA intake in all-day and three meals.

Supplementary Table S3. Adjusted hazard ratios for mortalities across quintiles of dietary PUFA intake in all-day and three meals.

Supplementary Table S4. Adjusted hazard ratios for mortalities across quintiles of dietary MUFA intake in all-day and three meals.

Supplementary Table S5. Association of the difference between unsaturated fats intakes of dinner and breakfast (%en) with mortality.

Supplementary Table S6. Association of the mortality with the difference level between unsaturated fats intakes (%E) at dinner and breakfast.

Supplementary Table S7. Isocaloric substitution model of the risk of mortality via replacing UFAs intake at breakfast with that at dinner.

Supplementary Table S8. Adjusted hazard ratios for mortalities stratified by gender across quintiles of dietary UFAs intake in all-day and three meals.

Supplementary Table S9. Adjusted hazard ratios for mortalities stratified by age group across quintiles of dietary UFAs intake in all-day and three meals.

Supplementary Table S10. Adjusted hazard ratios for mortalities across quintiles of dietary UFAs intake in all-day and three meals excluded participants with follow-up less than 2 years.

Supplementary Table S11. Adjusted hazard ratios for mortalities across quintiles of dietary UFAs intake in all-day and three meals excluded participants self-reported CVD.

Supplementary Table S12. Adjusted hazard ratios for mortalities across quintiles of dietary UFAs intake in all-day and three meals excluded participants with regular night shifts.

Supplementary Table S13. Adjusted hazard ratios for mortalities across quintiles of the difference between total consumption at lunch and dinner and breakfast.

Supplementary Figure S1. Associations of the difference in UFAs intakes between dinner and breakfast with the risk of hypertension mortality.

Supplementary Figure S2. Associations of the difference intake levels at breakfast and dinner of UFAs with the risk of hypertension mortality.

Supplementary Table S1. Baseline characteristics of participants according to quintile categories of total unsaturated fatty acids intake

Characteristics*	TUFA daily intake				
	**Quintile 1**	**Quintile 2**	**Quintile 3**	**Quintile 4**	**Quintile 5**
Participants, *n*	6027	6028	6028	6026	6027
Mean age, y	45.12 (44.37-45.87)	45.39 (44.66-46.13)	45.82 (45.17-46.48)	47 (46.27-47.73)	48.58 (47.8-49.35)
Female sex, *n (%)*	3063 (51.7)	3128 (52.4)	3064 (51.5)	2996 (51.1)	3052 (51.5)
Race/Ethnics, *n (%)*:					
Mexican American	1518 (13.4)	1137 (9.7)	940 (7.6)	835 (6.7)	688 (5.5)
Non-Hispanic white	2040 (58.0)	2673 (67.6)	2956 (71.7)	3103 (73.8)	3153 (74.2)
Non-Hispanic black	1160 (12.1)	1232 (11.0)	1223 (10.0)	1288 (10.7)	1487 (12.2)
Current smoking, *n (%)*	1340 (24.6)	1356 (23.0)	1379 (24.2)	1455 (23.9)	1545 (27.9)
Current drinking, *n (%)*	3595 (66.0)	3808 (69.5)	3886 (71.3)	3981 (72.3)	3900 (71.2)
Education level, *n (%)*:					
Less than 9th grade	1036 (9.4)	671 (5.8)	509 (4.3)	482 (4.4)	480 (4.5)
9-11th grade	993 (13.3)	856 (11.0)	822 (10.0)	917 (11.5)	891 (12.3)
High school	1452 (24.1)	1480 (23.4)	1579 (24.9)	1531 (24.2)	1635 (26.0)
College	1458 (29.1)	1741 (31.7)	1791 (32.3)	1792 (31.5)	1832 (32.6)
College graduate or above	1081 (24.0)	1272 (28.0)	1321 (28.6)	1303 (28.4)	1181 (24.5)
Annual household income, n (%):					
Under $20,000	1520 (18.5)	1315 (15.6)	1235 (14.8)	1219 (14.1)	1238 (14.4)
$20,000 to $45,000	2103 (31.6)	1944 (27.9)	1934 (27.3)	1933 (28.0)	1966 (29.0)
$45,000 to $75,000	958 (19.3)	1081 (20.5)	1153 (21.1)	1186 (21.8)	1168 (21.4)
$75,000 to $100,000	560 (12.0)	737 (16.0)	762 (17.3)	785 (17.5)	826 (17.9)
Over $100,000	561 (14.4)	678 (16.2)	674 (15.8)	667 (15.5)	616 (14.4)
BMI, kg/m^2	28.12 (27.84-28.4)	28.05 (27.81-28.28)	28.34 (28.13-28.54)	28.94 (28.71-29.17)	29.38 (29.07-29.69)
Exercised regularly, *n (%)*	3143 (44.3)	2766 (39.2)	2769 (41)	2801 (39.8)	2945 (44.9)
Dietary supplements use, *n (%)*	2559 (46.9)	2733 (49.6)	2848 (51.1)	2805 (51.1)	2749 (49.2)
Duration of sleep, *hour*	6.92 (6.87-6.98)	6.97 (6.92-7.02)	6.97 (6.92-7.01)	6.9 (6.86-6.93)	6.93 (6.88-6.98)
Regular night shift-work, *n (%)*	55 (0.9)	32 (0.5)	20 (0.2)	22 (0.4)	32 (0.5)

Family history of CVD, *n (%)*	1680 (32.6)	1719 (33.0)	1703 (33.6)	1760 (34.2)	1632 (31.2)
Ever had CVD, *n (%)*	585 (8.1)	614 (8.4)	661 (8.6)	655 (8.3)	707 (9.3)
Ever had diabetes, *n (%)*	719 (9.0)	650 (7.5)	633 (7.8)	707 (8.8)	723 (9.5)
Ever had hypertension, *n (%)*	1976 (29.9)	1981 (29.5)	2010 (29.9)	2058 (31.0)	2233 (34.6)
Ever had high cholesterol, *n (%)*	1735 (28.2)	1775 (29.3)	1908 (32.1)	1881 (31.5)	1973 (34.3)
Dietary intake					
Total energy, *kcal/d*	1890.05 (1861.54-1918.56)	2091.52 (2060.52-2122.52)	2144.12 (2115.33-2172.9)	2185.14 (2158.99-2211.29)	2180.49 (2151.34-2209.63)
Protein, *g/d*	73.53 (72.19-74.86)	82.31 (80.89-83.74)	84.39 (83.14-85.64)	85.91 (84.8-87.02)	86.23 (84.8-87.66)
Carbohydrate, g/d	264.7 (260.44-268.97)	269.49 (265.34-273.63)	263.15 (259.57-266.73)	252.59 (249.43-255.74)	223.9 (220.67-227.13)
Total Fat, *g/d*	50.77 (49.84-51.71)	70.26 (69.11-71.41)	80.04 (78.88-81.21)	89.96 (88.73-91.18)	104.02 (102.47-105.57)
TUFA, *g/d*	27.24 (26.76-27.71)	38.97 (38.4-39.54)	45.79 (45.17-46.42)	53.12 (52.45-53.78)	65.41 (64.39-66.43)
PUFA, *g/d*	9.88 (9.71-10.05)	14.19 (13.97-14.42)	17.05 (16.79-17.32)	20.26 (19.99-20.53)	26.3 (25.83-26.76)
MUFA, *g/d*	17.35 (17.01-17.7)	24.78 (24.37-25.19)	28.74 (28.32-29.17)	32.86 (32.39-33.32)	39.11 (38.49-39.74)
SFA, *g/d*	18.48 (18.05-18.91)	24.76 (24.24-25.28)	27.07 (26.59-27.55)	29.15 (28.63-29.66)	30.32 (29.82-30.83)
AHEI	52.87 (52.35-53.4)	50.6 (50.23-50.97)	49.34 (48.94-49.74)	48.52 (48.15-48.89)	48.85 (48.42-49.28)
Breakfast skipping, *n (%)*	237 (4.1)	262 (4.4)	328 (5.2)	400 (6.1)	450 (6.9)
Lunch skipping, *n (%)*	423 (5.5)	528 (6.3)	541 (6.4)	704 (9.0)	1105 (15.2)
Dinner skipping, *n (%)*	784 (9.9)	0	0	0	0
Mortality, *n (%)*:					
All-cause	852 (11.2)	846 (10.6)	924 (10.9)	902 (11.5)	986 (12.1)
CVD	327 (3.8)	339 (3.9)	364 (4.0)	373 (4.5)	398 (5.0)
Heart disease	203 (2.4)	207 (2.3)	257 (2.8)	253 (3.2)	249 (3.0)
Hypertension	132 (1.5)	147 (1.7)	135 (1.4)	134 (1.5)	154 (2.0)
	**TUFA intake at breakfast**				
	**Quintile 1**	**Quintile 2**	**Quintile 3**	**Quintile 4**	**Quintile 5**
Participants, *n*	6028	6027	6028	6027	6026
Mean age, y	42.96 (42.12-43.8)	47.53 (46.78-48.29)	47.95 (47.25-48.65)	47.45 (46.85-48.06)	46.62 (45.81-47.42)
Female sex, *n (%)*	2940 (49.1)	3315 (55.6)	3180 (53.0)	3017 (51.5)	2851 (48.1)
Race/Ethnics, *n (%)*:					
Mexican American	648 (5.6)	724 (5.5)	947 (7.1)	1237 (10.2)	1562 (15.5)

Non-Hispanic white	3103 (73.3)	3376 (76.2)	2982 (72.3)	2544 (66.6)	1920 (55.0)
Non-Hispanic black	1386 (11.0)	1057 (8.5)	1180 (10.0)	1350 (12.6)	1417 (14.7)
Current smoking, *n (%)*	1845 (30.7)	1185 (20.7)	1226 (21.4)	1366 (24.3)	1453 (26.6)
Current drinking, *n (%)*	3961 (72.8)	3797 (69.4)	3786 (69.5)	3852 (69.9)	3774 (69.1)
Education level, *n (%)*:					
Less than 9th grade	346 (3.2)	479 (4.1)	561 (4.6)	746 (7.0)	1046 (10.2)
9-11th grade	823 (10.3)	774 (10.0)	853 (11.4)	959 (12.4)	1070 (14.8)
High school	1660 (26.1)	1466 (23.2)	1497 (23.7)	1514 (23.9)	1540 (26.1)
College	1896 (33.5)	1745 (30.1)	1757 (31.3)	1692 (32.5)	1525 (29.9)
College graduate or above	1298 (26.9)	1562 (32.6)	1351 (29.0)	1107 (24.1)	840 (19.0)
Annual household income, n (%):					
Under $20,000	1261 (14.6)	1209 (13.9)	1238 (14.2)	1307 (15.4)	1512 (19.9)
$20,000 to $45,000	1898 (27.7)	1827 (26.5)	2003 (28.9)	2017 (29.1)	2135 (32.1)
$45,000 to $75,000	1082 (20.3)	1138 (20.6)	1114 (20.9)	1137 (21.7)	1075 (21.0)
$75,000 to $100,000	803 (17.6)	869 (18.5)	741 (16.5)	695 (15.3)	562 (12.5)
Over $100,000	726 (16.5)	760 (17.3)	683 (16.4)	588 (14.5)	439 (10.5)
BMI, kg/m^2	28.29 (28.03-28.55)	28.23 (27.96-28.5)	28.58 (28.33-28.84)	28.78 (28.53-29.03)	29.24 (28.93-29.55)
Exercised regularly, *n (%)*	2693 (40.5)	2599 (38.0)	2839 (41.0)	2995 (43.1)	3298 (48.0)
Dietary supplements use, *n (%)*	2416 (45.3)	3088 (54.9)	2978 (53.4)	2784 (49.9)	2428 (43.7)
Duration of sleep, *hour*	6.9 (6.85-6.94)	6.96 (6.92-7)	6.95 (6.9-6.99)	6.96 (6.92-7.01)	6.92 (6.87-6.96)
Regular night shift-work, *n (%)*	12 (0.2)	14 (0.3)	26 (0.4)	25 (0.4)	84 (1.3)
Family history of CVD, *n (%)*	1840 (34.5)	1745 (33.7)	1752 (32.7)	1607 (32.0)	1550 (30.9)
Ever had CVD, *n (%)*	521 (7.1)	640 (8.5)	683 (8.6)	699 (9.3)	679 (9.6)
Ever had diabetes, *n (%)*	437 (5.4)	632 (7.9)	687 (8.4)	776 (10.1)	900 (12.0)
Ever had hypertension, *n (%)*	1761 (27.6)	2060 (31.7)	2081 (31.6)	2179 (32.0)	2177 (33.0)
Ever had high cholesterol, *n (%)*	1554 (27.4)	1999 (32.5)	1915 (33.0)	1903 (31.2)	1901 (32.3)
Dietary intake					
Total energy, *kcal/d*	2076.91 (2049.04-2104.79)	2082.79 (2049.23-2116.35)	2155.6 (2130.33-2180.87)	2141.95 (2115.12-2168.77)	2054.81 (2028.29-2081.33)
Protein, *g/d*	80.48 (79-81.97)	82.24 (80.92-83.57)	84.46 (83.11-85.81)	84.69 (83.5-85.88)	81.67 (80.4-82.94)
Carbohydrate, g/d	254.33 (250.7-257.96)	260.06 (255.81-264.31)	263.54 (260.37-266.71)	254.15 (250.58-257.73)	234.92 (231.32-238.52)

Total Fat, *g/d*	76.26 (74.94-77.58)	75.96 (74.35-77.57)	80.86 (79.39-82.32)	83.59 (82.33-84.86)	84.04 (82.6-85.47)
TUFA, *g/d*	44.34 (43.54-45.15)	44.24 (43.25-45.22)	47.01 (46.16-47.85)	48.84 (48.03-49.65)	50.09 (49.23-50.96)
PUFA, *g/d*	16.85 (16.52-17.18)	17.09 (16.68-17.5)	17.97 (17.61-18.34)	18.44 (18.06-18.83)	18.9 (18.53-19.28)
MUFA, *g/d*	27.49 (26.97-28.02)	27.15 (26.53-27.76)	29.03 (28.5-29.56)	30.4 (29.92-30.88)	31.19 (30.63-31.75)
SFA, *g/d*	25.17 (24.69-25.65)	25 (24.42-25.59)	26.76 (26.21-27.3)	27.45 (26.99-27.9)	26.7 (26.17-27.23)
AHEI	47.97 (47.55-48.4)	52.34 (52.03-52.65)	50.91 (50.46-51.36)	49.9 (49.43-50.38)	48.41 (48.03-48.8)
Breakfast skipping, *n (%)*	1677 (24.2)	0	0	0	0
Lunch skipping, *n (%)*	363 (5.2)	435 (5.6)	494 (6.9)	687 (9.5)	1322 (18.4)
Dinner skipping, *n (%)*	78 (1.0)	102 (1.2)	125 (1.3)	192 (2.3)	287 (3.5)
Mortality, *n (%)*:					
All-cause	620 (7.5)	963 (11.6)	1017 (12.9)	998 (13.0)	912 (11.9)
CVD	246 (2.9)	393 (4.4)	403 (4.8)	385 (4.8)	374 (4.6)
Heart disease	149 (1.7)	258 (3.0)	255 (3.1)	267 (3.2)	240 (2.9)
Hypertension	96 (1.2)	156 (1.6)	162 (2.0)	146 (1.8)	142 (1.6)
	**TUFA intake at dinner**				
	**Quintile 1**	**Quintile 2**	**Quintile 3**	**Quintile 4**	**Quintile 5**
Participants, *n*	6027	6028	6028	6026	6027
Mean age, y	45.22 (44.54-45.91)	45.87 (45.24-46.51)	46.22 (45.44-46.99)	46.87 (46.08-47.67)	47.74 (46.93-48.55)
Female sex, *n (%)*	3063 (51.7)	3128 (52.4)	3064 (51.5)	2996 (51.1)	3052 (51.5)
Race/Ethnics, *n (%)*:					
Mexican American	1518 (13.4)	1137 (9.7)	940 (7.6)	835 (6.7)	688 (5.5)
Non-Hispanic white	2040 (58.0)	2673 (67.6)	2956 (71.7)	3103 (73.8)	3153 (74.2)
Non-Hispanic black	1160 (12.1)	1232 (11.0)	1223 (10.0)	1288 (10.7)	1487 (12.2)
Current smoking, *n (%)*	1340 (24.6)	1356 (23.0)	1379 (24.2)	1455 (23.9)	1545 (27.9)
Current drinking, *n (%)*	3595 (66.0)	3808 (69.5)	3886 (71.3)	3981 (72.3)	3900 (71.2)
Education level, *n (%)*:					
Less than 9th grade	1036 (9.4)	671 (5.8)	509 (4.3)	482 (4.4)	480 (4.5)
9-11th grade	993 (13.3)	856 (11.0)	822 (10.0)	917 (11.5)	891 (12.3)
High school	1452 (24.1)	1480 (23.4)	1579 (24.9)	1531 (24.2)	1635 (26.0)
College	1459 (29.1)	1741 (31.7)	1791 (32.3)	1792 (31.5)	1832 (32.6)

College graduate or above	1081 (24.0)	1272 (28.0)	1321 (28.6)	1303 (28.4)	1181 (24.5)
Annual household income, n (%):					
Under $20,000	1520 (18.5)	1315 (15.6)	1235 (14.8)	1219 (14.1)	1238 (14.4)
$20,000 to $45,000	2103 (31.6)	1944 (27.9)	1934 (27.3)	1933 (28.0)	1966 (29.0)
$45,000 to $75,000	958 (19.3)	1081 (20.5)	1153 (21.1)	1186 (21.8)	1168 (21.4)
$75,000 to $100,000	560 (12.0)	737 (16.0)	762 (17.3)	785 (17.5)	826 (17.9)
Over $100,000	561 (14.4)	678 (16.2)	674 (15.8)	667 (15.5)	616 (14.4)
BMI, kg/m^2	28.27 (28.02-28.52)	28.31 (28.02-28.61)	28.4 (28.14-28.66)	28.68 (28.45-28.92)	29.19 (28.93-29.45)
Exercised regularly, *n (%)*	3143 (44.3)	2766 (39.2)	2769 (41.0)	2801 (39.8)	2945 (44.9)
Dietary supplements use, *n (%)*	2559 (46.9)	2733 (49.6)	2848 (51.1)	2805 (51.1)	2749 (49.2)
Duration of sleep, *hour*	6.92 (6.87-6.97)	6.98 (6.94-7.03)	6.95 (6.9-6.99)	6.93 (6.88-6.97)	6.91 (6.86-6.95)
Regular night shift-work, *n (%)*	55 (0.9)	32 (0.5)	20 (0.2)	22 (0.4)	32 (0.5)
Family history of CVD, *n (%)*	1680 (32.6)	1719 (33.0)	1703 (33.6)	1760 (34.2)	1632 (31.2)
Ever had CVD, *n (%)*	585 (8.1)	614 (8.4)	661 (8.6)	655 (8.3)	707 (9.3)
Ever had diabetes, *n (%)*	719 (9.0)	650 (7.5)	633 (7.8)	707 (8.8)	723 (9.5)
Ever had hypertension, *n (%)*	1976 (29.9)	1981 (29.5)	2010 (29.9)	2058 (31.0)	2233 (34.6)
Ever had high cholesterol, *n (%)*	1735 (28.2)	1775 (29.3)	1908 (32.1)	1881 (31.5)	1973 (34.3)
Dietary intake					
Total energy, *kcal/d*	2047.38 (2018.75-2076.02)	2121.23 (2089.75-2152.7)	2149.17 (2120.28-2178.05)	2118.24 (2090.12-2146.36)	2069.65 (2037.22-2102.08)
Protein, *g/d*	79.61 (78.07-81.15)	83.47 (82.03-84.91)	84.87 (83.67-86.08)	83.89 (82.63-85.15)	81.03 (79.53-82.52)
Carbohydrate, g/d	261.92 (257.93-265.92)	266.71 (262.66-270.77)	262.83 (259.07-266.59)	249.57 (245.77-253.37)	232.21 (229.18-235.23)
Total Fat, *g/d*	69.99 (68.73-71.25)	75.84 (74.43-77.25)	80.37 (79.05-81.69)	83.09 (81.65-84.52)	87.76 (85.97-89.55)
TUFA, *g/d*	40.7 (39.93-41.47)	43.62 (42.82-44.41)	46.6 (45.81-47.38)	48.4 (47.55-49.26)	52.77 (51.64-53.89)
PUFA, *g/d*	15.46 (15.11-15.81)	16.38 (16.07-16.69)	17.52 (17.21-17.84)	18.27 (17.9-18.64)	20.75 (20.24-21.26)
MUFA, *g/d*	25.24 (24.77-25.71)	27.24 (26.71-27.77)	29.08 (28.56-29.59)	30.13 (29.6-30.66)	32.02 (31.35-32.69)
SFA, *g/d*	23.04 (22.55-23.53)	25.37 (24.81-25.93)	26.68 (26.17-27.19)	27.42 (26.9-27.93)	27.61 (27.01-28.21)
AHEI	51.58 (51.17-51.99)	51.12 (50.63-51.62)	50.26 (49.89-50.63)	49.05 (48.62-49.47)	48.19 (47.73-48.65)
Breakfast skipping, *n (%)*	237 (4.1)	262 (4.4)	328 (5.2)	400 (6.1)	450 (6.9)
Lunch skipping, *n (%)*	423 (5.5)	528 (6.3)	541 (6.4)	704 (9.0)	1105 (15.2)
Dinner skipping, *n (%)*	784 (9.9)	0	0	0	0

Mortality, *n (%)*:					
All-cause	852 (11.2)	846 (10.6)	924 (10.9)	902 (11.5)	986 (12.1)
CVD	327 (3.8)	339 (3.9)	364 (4.0)	373 (4.5)	398 (5.0)
Heart disease	203 (2.4)	207 (2.3)	257 (2.8)	253 (3.2)	249 (3.0)
Hypertension	132 (1.5)	147 (1.7)	135 (1.4)	134 (1.5)	154 (2.0)

[*]Continuous variables were presented as weighted mean (95% CI), and categorical variables were presented as unweighted number (weighted percentage).

Supplementary Table S2. Adjusted hazard ratios for mortalities across quintiles of dietary TUFA intake in all-day and three meals.

Variable	%E (SE)	N	All-cause Mortality			CVD Mortality			Heart Mortality			Hypertension Mortality		
			Case	HR (95% CI)	P value	Case	HR (95% CI)	P value	Case	HR (95% CI)	P value	Case	HR (95% CI)	P value
**Daily TUFA Intake**														
Q1	12.86 (0.03)	6027	917	1 (Reference)	-	354	1 (Reference)	-	223	1 (Reference)	-	147	1 (Reference)	-
Q2	16.76 (0.01)	6031	893	0.92 (0.83-1.03)	0.137	386	1.10 (0.91-1.32)	0.311	252	1.13 (0.90-1.41)	0.285	145	0.95 (0.70-1.30)	0.754
Q3	19.21 (0.01)	6025	885	0.89 (0.79-1.00)	0.045	352	1.00 (0.83-1.19)	0.973	214	0.98 (0.79-1.22)	0.865	143	0.89 (0.68-1.15)	0.347
Q4	21.87 (0.01)	6027	877	0.84 (0.75-0.95)	0.007	339	0.92 (0.75-1.12)	0.379	234	0.94 (0.74-1.18)	0.581	128	0.79 (0.57-1.10)	0.164
Q5	26.96 (0.06)	6026	938	0.81 (0.72-0.92)	0.001	370	0.95 (0.78-1.15)	0.574	246	0.97 (0.76-1.25)	0.831	139	0.90 (0.68-1.20)	0.465
per SD				0.92 (0.88-0.96)	<0.001		0.95 (0.90-1.01)	0.114		0.97 (0.90-1.05)	0.407		0.93 (0.86-1.01)	0.083
P for linear					0.001			0.151			0.529			0.601
P for nonlinear					0.417			0.497			0.26			0.346
**TUFA Intake at Breakfast**														
Q1	0.32 (0.01)	6028	620	1 (Reference)	-	246	1 (Reference)	-	149	1 (Reference)	-	96	1 (Reference)	-
Q2	1.62 (0.01)	6027	963	1.18 (1.03-1.36)	0.021	393	1.17 (0.96-1.42)	0.121	258	1.27 (0.98-1.64)	0.072	156	1.01 (0.75-1.36)	0.933
Q3	3.27 (0.01)	6028	1017	1.35 (1.17-1.57)	<0.001	403	1.33 (1.07-1.65)	0.010	255	1.41 (1.10-1.80)	0.007	162	1.23 (0.86-1.78)	0.254
Q4	5.57 (0.01)	6027	998	1.26 (1.08-1.46)	0.004	385	1.19 (0.95-1.50)	0.124	267	1.29 (1.00-1.66)	0.048	146	1.05 (0.71-1.56)	0.802
Q5	10.69 (0.06)	6026	912	1.18 (1.01-1.38)	0.040	374	1.20 (0.94-1.53)	0.141	240	1.23 (0.92-1.64)	0.154	142	0.97 (0.68-1.39)	0.853
per SD				1.03 (0.98-1.07)	0.231		1.03 (0.96-1.10)	0.492		1.02 (0.94-1.11)	0.632		0.97 (0.87-1.09)	0.655
P for linear					<0.001			0.247			0.214			0.976
P for nonlinear					<0.001			0.014			0.099			0.315
**TUFA Intake at Lunch**														
Q1	0.58 (0.01)	6027	1031	1 (Reference)	-	411	1 (Reference)	-	268	1 (Reference)	-	145	1 (Reference)	-
Q2	3.58 (0.01)	6029	945	1.14 (0.98-1.32)	0.094	378	1.22 (0.95-1.57)	0.120	237	1.18 (0.83-1.68)	0.348	159	1.44 (1.01-2.06)	0.045
Q3	5.96 (0.01)	6026	880	1.04 (0.91-1.19)	0.584	349	1.16 (0.91-1.49)	0.225	215	1.12 (0.83-1.51)	0.453	134	1.20 (0.88-1.64)	0.248
Q4	8.64 (0.02)	6028	865	0.97 (0.85-1.11)	0.687	345	1.08 (0.88-1.32)	0.477	233	1.07 (0.08-1.44)	0.636	146	1.37 (1.00-1.88)	0.050
Q5	14.61 (0.07)	6026	789	0.99 (0.86-1.15)	0.940	318	1.11 (0.89-1.37)	0.362	216	1.16 (0.89-1.51)	0.286	118	1.20 (0.82-1.76)	0.343
per SD				0.95 (0.91-0.99)	0.039		0.98 (0.93-1.03)	0.339		1.00 (0.94-1.06)	0.932		1.00 (0.91-1.10)	0.998
P for linear					0.124			0.934			0.603			0.962
P for nonlinear					0.155			0.241			0.084			0.417

**TUFA Intake at Dinner**														
Q1	2.62 (0.02)	6027	852	1 (Reference)	-	327	1 (Reference)	-	203	1 (Reference)	-	132	1 (Reference)	-
Q2	5.65 (0.01)	6028	846	0.96 (0.85-1.09)	0.544	339	1.03 (0.84-1.27)	0.771	207	1.01 (0.77-1.31)	0.966	147	1.11 (0.80-1.53)	0.526
Q3	7.98 (0.01)	6028	924	0.94 (0.82-1.07)	0.319	364	0.99 (0.83-1.18)	0.923	257	1.09 (0.87-1.35)	0.452	135	0.97 (0.72-1.31)	0.825
Q4	10.79 (0.02)	6026	902	0.91 (0.80-1.02)	0.113	373	1.03 (0.86-1.24)	0.737	253	1.12 (0.89-1.41)	0.324	134	0.91 (0.67-1.23)	0.526
Q5	17.19 (0.08)	6027	986	0.87 (0.77-0.98)	0.027	398	0.99 (0.82-1.20)	0.938	249	0.94 (0.73-1.21)	0.632	154	1.06 (0.79-1.41)	0.708
per SD				0.94 (0.91-0.98)	0.003		0.96 (0.92-1.02)	0.181		0.95 (0.89-1.01)	0.099		0.97 (0.89-1.06)	0.484
P for linear					0.020			0.896			0.482			0.826
P for nonlinear					0.542			0.204			0.016			0.447

Supplementary Table S3. Adjusted hazard ratios for mortalities across quintiles of dietary PUFA intake in all-day and three meals.

Variable	%E (SE)	N	All-cause Mortality			CVD Mortality			Heart Mortality			Hypertension Mortality		
			Case	HR (95% CI)	P value	Case	HR (95% CI)	P value	Case	HR (95% CI)	P value	Case	HR (95% CI)	P value
**Daily PUFA Intake**														
Q1	4.21 (0.01)	6026	991	1 (Reference)	-	398	1 (Reference)	-	262	1 (Reference)	-	150	1 (Reference)	-
Q2	5.89 (0.01)	6035	910	0.93 (0.82-1.06)	0.266	380	0.96 (0.81-1.15)	0.675	240	0.93 (0.74-1.17)	0.543	151	1.03 (0.78-1.35)	0.855
Q3	7.10 (0.01)	6025	896	0.86 (0.76-0.97)	0.014	347	0.85 (0.70-1.03)	0.089	224	0.87 (0.70-1.09)	0.215	144	0.88 (0.66-1.19)	0.401
Q4	8.51 (0.01)	6026	880	0.83 (0.74-0.92)	0.001	344	0.85 (0.72-1.01)	0.071	219	0.81 (0.66-0.99)	0.043	135	0.87 (0.66-1.14)	0.301
Q5	11.56 (0.04)	6024	833	0.77 (0.67-0.89)	<0.001	332	0.84 (0.69-1.04)	0.106	224	0.87 (0.66-1.14)	0.305	122	0.86 (0.66-1.14)	0.290
per SD				0.92 (0.87-0.96)	<0.001		0.93 (0.87-0.99)	0.036		0.96 (0.87-1.06)	0.419		0.94 (0.86-1.04)	0.216
P for linear					<0.001			<0.001			0.423			0.436
P for nonlinear					0.205			0.243			0.773			0.703
**PUFA Intake at Breakfast**														
Q1	0.10 (0.001)	6032	602	1 (Reference)	-	226	1 (Reference)	-	137	1 (Reference)	-	90	1 (Reference)	-
Q2	0.53 (0.002)	6029	960	1.16 (1.02-1.32)	0.027	392	1.19 (1.01-1.41)	0.042	252	1.27 (1.02-1.61)	0.043	157	0.98 (0.74-1.30)	0.869
Q3	1.10 (0.004)	6026	1051	1.30 (1.13-1.50)	<0.001	429	1.38 (1.12-1.70)	0.003	278	1.43 (1.09-1.88)	0.011	174	1.32 (0.94-1.86)	0.112
Q4	1.94 (0.004)	6025	984	1.18 (1.02-1.36)	0.029	380	1.21 (0.98-1.49)	0.073	248	1.23 (0.94-1.62)	0.131	150	0.96 (0.69-1.33)	0.786
Q5	4.06 (0.03)	6024	913	1.11 (0.95-1.30)	0.172	374	1.17 (0.94-1.45)	0.167	254	1.30 (0.98-1.73)	0.072	131	0.86 (0.59-1.24)	0.406
per SD				1.01 (0.96-1.06)	0.751		1.01 (0.95-1.08)	0.746		1.03 (0.95-1.12)	0.516		0.95 (0.85-1.07)	0.427
P for linear					<0.001			0.001			0.144			0.557
P for nonlinear					<0.001			0.001			0.111			0.633
**PUFA Intake at Lunch**														
Q1	0.19 (0.004)	6025	1068	1 (Reference)	-	421	1 (Reference)	-	280	1 (Reference)	-	148	1 (Reference)	-
Q2	1.20 (0.004)	6028	961	1.09 (0.92-1.28)	0.322	383	1.17 (0.92-1.47)	0.192	237	1.13 (0.84-1.52)	0.410	162	1.24 (0.90-1.70)	0.193
Q3	2.16 (0.004)	6031	875	0.99 (0.87-1.12)	0.860	356	1.04 (0.84-1.29)	0.731	226	0.99 (0.76-1.30)	0.942	143	1.17 (0.82-1.68)	0.378
Q4	3.36 (0.01)	6024	830	0.96 (0.83-1.10)	0.519	326	1.03 (0.83-1.28)	0.774	220	1.05 (0.81-1.36)	0.717	127	1.11 (0.78-1.59)	0.566
Q5	6.47 (0.05)	6028	776	0.90 (0.78-1.03)	0.129	315	1.00 (0.83-1.21)	0.994	206	1.00 (0.78-1.27)	0.997	122	1.12 (0.76-1.63)	0.571
per SD				0.95 (0.91-0.99)	0.025		0.95 (0.90-0.99)	0.046		0.97 (0.90-1.03)	0.311		0.95 (0.86-1.06)	0.376
P for linear					0.005			0.092			0.736			0.808
P for nonlinear					0.822			0.082			0.155			0.507

PUFA Intake at Dinner														
Q1	0.82 (0.01)	6030	915	1 (Reference)	-	362	1 (Reference)	-	221	1 (Reference)	-	151	1 (Reference)	-
Q2	1.90 (0.004)	6028	851	0.93 (0.82-1.04)	0.192	344	0.96 (0.78-1.18)	0.678	236	1.09 (0.84-1.43)	0.513	133	0.93 (0.71-1.23)	0.622
Q3	2.85 (0.004)	6027	883	0.90 (0.79-1.01)	0.074	338	0.88 (0.73-1.06)	0.176	216	0.89 (0.71-1.12)	0.324	135	0.86 (0.64-1.16)	0.327
Q4	4.11 (0.01)	6029	921	0.93 (0.83-1.03)	0.165	378	0.98 (0.82-1.17)	0.817	258	1.12 (0.89-1.39)	0.328	141	0.84 (0.64-1.11)	0.223
Q5	7.42 (0.05)	6022	940	0.81 (0.73-0.91)	<0.001	379	0.86 (0.71-1.04)	0.114	238	0.86 (0.67-1.09)	0.207	142	0.86 (0.65-1.13)	0.267
per SD				0.93 (0.90-0.96)	<0.001		0.94 (0.89-0.99)	0.041		0.95 (0.88-1.01)	0.117		0.97 (0.88-1.07)	0.559
P for linear					0.001			0.379			0.806			0.549
P for nonlinear					0.639			0.641			0.13			0.849

Supplementary Table S4. Adjusted hazard ratios for mortalities across quintiles of dietary MUFA intake in all-day and three meals.

Variable	%E (SE)	N	All-cause Mortality			CVD Mortality			Heart Mortality			Hypertension Mortality		
			Case	HR (95% CI)	P value	Case	HR (95% CI)	P value	Case	HR (95% CI)	P value	Case	HR (95% CI)	P value
**Daily MUFA Intake**														
Q1	4.21 (0.01)	6026	991	1 (Reference)	-	398	1 (Reference)	-	262	1 (Reference)	-	150	1 (Reference)	-
Q2	5.89 (0.01)	6035	910	0.93 (0.82-1.06)	0.266	380	0.96 (0.81-1.15)	0.675	240	0.93 (0.74-1.17)	0.543	151	1.03 (0.78-1.35)	0.855
Q3	7.10 (0.01)	6025	896	0.86 (0.76-0.97)	0.014	347	0.85 (0.70-1.03)	0.089	224	0.87 (0.70-1.09)	0.215	144	0.88 (0.66-1.19)	0.401
Q4	8.51 (0.01)	6026	880	0.83 (0.74-0.92)	0.001	344	0.85 (0.72-1.01)	0.071	219	0.81 (0.66-0.99)	0.043	135	0.87 (0.66-1.14)	0.301
Q5	11.56 (0.04)	6024	833	0.77 (0.67-0.89)	<0.001	332	0.84 (0.69-1.04)	0.106	224	0.87 (0.66-1.14)	0.305	122	0.86 (0.66-1.14)	0.290
per SD				0.92 (0.87-0.96)	<0.001		0.93 (0.87-0.99)	0.036		0.96 (0.87-1.06)	0.419		0.94 (0.86-1.04)	0.216
P for linear					<0.001			<0.001			0.423			0.436
P for nonlinear					0.205			0.243			0.773			0.703
**MUFA Intake at Breakfast**														
Q1	0.10 (0.001)	6032	602	1 (Reference)	-	226	1 (Reference)	-	137	1 (Reference)	-	90	1 (Reference)	-
Q2	0.53 (0.002)	6029	960	1.16 (1.02-1.32)	0.027	392	1.19 (1.01-1.41)	0.042	252	1.27 (1.02-1.61)	0.043	157	0.98 (0.74-1.30)	0.869
Q3	1.10 (0.004)	6026	1051	1.30 (1.13-1.50)	<0.001	429	1.38 (1.12-1.70)	0.003	278	1.43 (1.09-1.88)	0.011	174	1.32 (0.94-1.86)	0.112
Q4	1.94 (0.004)	6025	984	1.18 (1.02-1.36)	0.029	380	1.21 (0.98-1.49)	0.073	248	1.23 (0.94-1.62)	0.131	150	0.96 (0.69-1.33)	0.786
Q5	4.06 (0.03)	6024	913	1.11 (0.95-1.30)	0.172	374	1.17 (0.94-1.45)	0.167	254	1.30 (0.98-1.73)	0.072	131	0.86 (0.59-1.24)	0.406
per SD				1.01 (0.96-1.06)	0.751		1.01 (0.95-1.08)	0.746		1.03 (0.95-1.12)	0.516		0.95 (0.85-1.07)	0.427
P for linear					<0.001			0.001			0.144			0.557
P for nonlinear					<0.001			0.001			0.111			0.633
**MUFA Intake at Lunch**														
Q1	0.19 (0.004)	6025	1068	1 (Reference)	-	421	1 (Reference)	-	280	1 (Reference)	-	148	1 (Reference)	-
Q2	1.20 (0.004)	6028	961	1.09 (0.92-1.28)	0.322	383	1.17 (0.92-1.47)	0.192	237	1.13 (0.84-1.52)	0.410	162	1.24 (0.90-1.70)	0.193
Q3	2.16 (0.004)	6031	875	0.99 (0.87-1.12)	0.860	356	1.04 (0.84-1.29)	0.731	226	0.99 (0.76-1.30)	0.942	143	1.17 (0.82-1.68)	0.378
Q4	3.36 (0.01)	6024	830	0.96 (0.83-1.10)	0.519	326	1.03 (0.83-1.28)	0.774	220	1.05 (0.81-1.36)	0.717	127	1.11 (0.78-1.59)	0.566
Q5	6.47 (0.05)	6028	776	0.90 (0.78-1.03)	0.129	315	1.00 (0.83-1.21)	0.994	206	1.00 (0.78-1.27)	0.997	122	1.12 (0.76-1.63)	0.571
per SD				0.95 (0.91-0.99)	0.025		0.95 (0.90-0.99)	0.046		0.97 (0.90-1.03)	0.311		0.95 (0.86-1.06)	0.376
P for linear					0.005			0.092			0.736			0.808
P for nonlinear					0.822			0.082			0.155			0.507

MUFA Intake at Dinner														
Q1	0.82 (0.01)	6030	915	1 (Reference)	-	362	1 (Reference)	-	221	1 (Reference)	-	151	1 (Reference)	-
Q2	1.90 (0.004)	6028	851	0.93 (0.82-1.04)	0.192	344	0.96 (0.78-1.18)	0.678	236	1.09 (0.84-1.43)	0.513	133	0.93 (0.71-1.23)	0.622
Q3	2.85 (0.004)	6027	883	0.90 (0.79-1.01)	0.074	338	0.88 (0.73-1.06)	0.176	216	0.89 (0.71-1.12)	0.324	135	0.86 (0.64-1.16)	0.327
Q4	4.11 (0.01)	6029	921	0.93 (0.83-1.03)	0.165	378	0.98 (0.82-1.17)	0.817	258	1.12 (0.89-1.39)	0.328	141	0.84 (0.64-1.11)	0.223
Q5	7.42 (0.05)	6022	940	0.81 (0.73-0.91)	<0.001	379	0.86 (0.71-1.04)	0.114	238	0.86 (0.67-1.09)	0.207	142	0.86 (0.65-1.13)	0.267
per SD				0.93 (0.90-0.96)	<0.001		0.94 (0.89-0.99)	0.041		0.95 (0.88-1.01)	0.117		0.97 (0.88-1.07)	0.559
P for linear					0.001			0.379			0.806			0.549
P for nonlinear					0.639			0.641			0.13			0.849

Supplementary Table S5. Association of the difference between unsaturated fats intakes of dinner and breakfast (%en) with mortality.

Variable	N	All-cause Mortality			CVD Mortality			Heart Mortality			Hypertension Mortality		
		Case	HR (95% CI)	P value	Case	HR (95% CI)	P value	Case	HR (95% CI)	P value	Case	HR (95% CI)	P value
**ΔTUFA Intake**													
Q1	6027	889	1 (Reference)	-	357	1 (Reference)	-	227	1 (Reference)	-	136	1 (Reference)	-
Q2	6027	936	1.01 (0.88-1.15)	0.941	369	0.98 (0.78-1.22)	0.844	231	0.92 (0.70-1.22)	0.578	166	1.22 (0.91-1.63)	0.182
Q3	6026	916	1.00 (0.86-1.16)	0.989	368	1.05 (0.84-1.32)	0.675	260	1.15 (0.86-1.55)	0.334	128	0.96 (0.68-1.34)	0.797
Q4	6030	853	0.87 (0.76-0.99)	0.043	335	0.90 (0.73-1.12)	0.345	225	0.90 (0.69-1.16)	0.402	126	0.98 (0.73-1.32)	0.901
Q5	6026	916	0.87 (0.77-0.98)	0.033	372	0.99 (0.82-1.20)	0.940	226	0.93 (0.72-1.20)	0.591	146	1.16 (0.84-1.61)	0.375
per SD			0.94 (0.90-0.97)	0.003		0.98 (0.93-1.03)	0.356		0.96 (0.91-1.03)	0.262		0.99 (0.90-1.10)	0.959
P for linear				0.003			0.739			0.495			0.838
P for nonlinear				0.106			0.063			0.056			0.222
**ΔPUFA Intake**													
Q1	6028	903	1 (Reference)	-	369	1 (Reference)	-	242	1 (Reference)	-	136	1 (Reference)	-
Q2	6026	953	1.02 (0.89-1.16)	0.773	376	0.96 (0.76-1.20)	0.703	241	0.94 (0.72-1.24)	0.673	154	1.06 (0.77-1.47)	0.717
Q3	6028	902	1.06 (0.92-1.21)	0.414	365	1.07 (0.85-1.36)	0.562	243	1.09 (0.84-1.43)	0.513	157	1.23 (0.85-1.77)	0.281
Q4	6027	872	0.92 (0.81-1.06)	0.247	341	0.98 (0.79-1.22)	0.869	232	0.99 (0.77-1.27)	0.911	116	0.91 (0.61-1.36)	0.652
Q5	6027	880	0.88 (0.78-0.99)	0.044	350	0.91 (0.75-1.12)	0.373	211	0.84 (0.65-1.07)	0.152	139	1.06 (0.75-1.52)	0.732
per SD			0.94 (0.91-0.97)	0.002		0.97 (0.92-1.02)	0.246		0.94 (0.88-0.99)	0.048		0.99 (0.90-1.09)	0.897
P for linear				0.006			0.409			0.154			0.933
P for nonlinear				0.050			0.154			0.091			0.453
**ΔMUFA Intake**													
Q1	6027	883	1 (Reference)	-	358	1 (Reference)	-	226	1 (Reference)	-	134	1 (Reference)	-
Q2	6026	925	0.98 (0.86-1.11)	0.756	367	0.97 (0.79-1.20)	0.805	234	1.02 (0.78-1.33)	0.911	165	1.29 (0.95-1.76)	0.104
Q3	6032	905	0.98 (0.84-1.15)	0.820	346	0.97 (0.77-1.22)	0.793	241	1.09 (0.81-1.48)	0.552	131	1.03 (0.75-1.43)	0.838
Q4	6026	887	0.93 (0.80-1.08)	0.357	354	1.01 (0.79-1.29)	0.943	230	1.07 (0.78-1.46)	0.680	132	1.08 (0.78-1.49)	0.651
Q5	6025	910	0.85 (0.75-0.97)	0.015	376	0.94 (0.77-1.16)	0.567	238	0.94 (0.73-1.22)	0.647	140	1.14 (0.80-1.64)	0.471
per SD			0.94 (0.91-0.98)	0.008		0.98 (0.93-1.04)	0.529		0.98 (0.92-1.05)	0.650		1.00 (0.90-1.12)	0.998
P for linear				0.009			0.735			0.700			0.929
P for nonlinear				0.501			0.222			0.206			0.204

Supplementary Table S6. Association of the mortality with the difference level between unsaturated fats intakes (%E) at dinner and breakfast.

Variable	N	All-cause Mortaility			CVD Mortaility			Heart Mortaility			Hypertension Mortaility		
		Case	HR (95% CI)	P value	Case	HR (95% CI)	P value	Case	HR (95% CI)	P value	Case	HR (95% CI)	P value
**TUFA Intake**													
BL-DH	8199	1174	1 (Reference)	-	485	1 (Reference)	-	313	1 (Reference)	-	187	1 (Reference)	-
BH-DH	6867	1203	1.11 (0.98-1.25)	0.089	788	1.07 (0.88-1.30)	0.483	327	1.09 (0.86-1.37)	0.471	183	1.03 (0.76-1.40)	0.858
BL-DL	6870	925	1.05 (0.94-1.17)	0.362	353	0.90 (0.77-1.05)	0.186	225	0.93 (0.76-1.13)	0.456	145	0.95 (0.74-1.21)	0.663
BH-DL	8200	1208	1.16 (1.02-1.31)	0.021	475	1.01 (0.83-1.22)	0.960	304	0.96 (0.77-1.19)	0.684	187	1.03 (0.73-1.45)	0.864
**PUFA Intake**													
BL-DH	8054	1108	1 (Reference)	-	431	1 (Reference)	-	271	1 (Reference)	-	163	1 (Reference)	-
BH-DH	7010	1197	1.08 (0.98-1.19)	0.136	482	1.12 (0.93-1.35)	0.246	326	1.12 (0.89-1.39)	0.335	179	1.05 (0.80-1.39)	0.713
BL-DL	7019	943	1.10 (0.98-1.24)	0.108	379	1.09 (0.90-1.33)	0.354	234	1.09 (0.88-1.36)	0.432	166	1.22 (0.92-1.61)	0.166
BH-DL	8053	1262	1.23 (1.10-1.37)	0.001	509	1.18 (1.01-1.37)	0.033	338	1.22 (1.01-1.48)	0.045	194	1.14 (0.87-1.50)	0.342
**MUFA Intake**													
BL-DH	8140	1162	1 (Reference)	-	470	1 (Reference)	-	303	1 (Reference)	-	187	1 (Reference)	-
BH-DH	6926	1227	1.15 (1.00-1.31)	0.048	491	1.14 (0.95-1.37)	0.166	325	1.12 (0.91-1.37)	0.281	181	1.09 (0.80-1.49)	0.595
BL-DL	6932	958	1.07 (0.94-1.22)	0.279	362	1.00 (0.84-1.18)	0.982	240	1.05 (0.85-1.30)	0.651	138	0.92 (0.70-1.19)	0.514
BH-DL	8138	1163	1.14 (1.01-1.30)	0.037	478	1.08 (0.90-1.31)	0.401	301	0.98 (0.79-1.21)	0.976	196	1.17 (0.83-1.65)	0.369

BL-DL represented the participants who with Lower intake at Breakfast and Lower intake at Dinner; BH-DL represented the participants who with Higher intake at Breakfast and Lower intake at Dinner; BH-DH represented the participants who with Higher intake at Breakfast and Higher intake at Dinner; BL-DH, as the refernce group, represented the participants who with Lower intake at Breakfast and Higher intake at Dinner.

Supplementary Table S7. Isocaloric substitution model of the risk of mortality via replacing UFAs intake at breakfast with that at dinner.

**Mortality**	**HR (95% CI)**	**P value**
**All-Cause**		
TUFA	0.84 (0.74-0.94)	0.005
PUFA	0.72 (0.54-0.98)	0.034
MUFA	0.76 (0.64-0.91)	0.003
**CVD**		
TUFA	0.92 (0.77-1.10)	0.385
PUFA	0.83 (0.56-1.23)	0.345
MUFA	0.89 (0.67-1.18)	0.430
**Heart Disease**		
TUFA	0.91 (0.74-1.13)	0.383
PUFA	0.70 (0.43-1.16)	0.165
MUFA	0.93 (0.66-1.31)	0.685
**Hypertension**		
TUFA	1.03 (0.73-1.46)	0.852
PUFA	1.20 (0.54-2.67)	0.652
MUFA	1.02 (0.60-1.75)	0.938

Supplementary Table S8. Adjusted hazard ratios for mortalities stratified by gender across quintiles of dietary UFAs intake in all-day and three meals.

Variable	N	All-cause Mortaility		
		Case	HR (95% CI)	P value
**Female**				
**Daily TUFA Intake**				
Q1	3062	430	1 (Reference)	-
Q2	3008	401	0.82 (0.70-0.97)	0.018
Q3	2992	381	0.81 (0.66-0.99)	0.040
Q4	3093	376	0.71 (0.57-0.88)	0.002
Q5	3148	391	0.67 (0.56-0.81)	<0.001
**TUFA Intake at Breakfast**				
Q1	2940	270	1 (Reference)	-
Q2	3315	464	1.25 (1.01-1.55)	0.038
Q3	3180	484	1.38 (1.13-1.70)	0.002
Q4	3017	426	1.26 (1.00-1.58)	0.050
Q5	2851	335	1.03 (0.82-1.31)	0.777
**TUFA Intake at Lunch**				
Q1	2920	410	1 (Reference)	-
Q2	3111	422	1.17 (0.92-1.48)	0.191
Q3	3073	386	1.04 (0.83-1.30)	0.745
Q4	3105	417	1.06 (0.84-1.32)	0.640
Q5	3094	344	0.9 (0.73-1.12)	0.347
**TUFA Intake at Dinner**				
Q1	3063	376	1 (Reference)	-
Q2	3128	379	1.01 (0.82-1.25)	0.930
Q3	3064	410	0.97 (0.79-1.18)	0.724
Q4	2996	398	0.98 (0.81-1.19)	0.832
Q5	3052	416	0.85 (0.69-1.04)	0.115
**Male**				
**Daily TUFA Intake**				
Q1	2965	487	1 (Reference)	-
Q2	3023	492	1.02 (0.85-1.22)	0.850
Q3	3033	504	0.98 (0.80-1.18)	0.801
Q4	2934	501	0.95 (0.79-1.15)	0.588
Q5	2878	547	0.93 (0.78-1.11)	0.434
**TUFA Intake at Breakfast**				
Q1	3088	350	1 (Reference)	-
Q2	2712	499	1.23 (1.01-1.49)	0.041
Q3	2848	533	1.42 (1.13-1.79)	0.003
Q4	3010	572	1.41 (1.16-1.72)	0.001
Q5	3175	577	1.41 (1.14-1.73)	0.001
**TUFA Intake at Lunch**				

Q1	3107	621	1 (Reference)	-
Q2	2918	523	1.16 (0.95-1.41)	0.145
Q3	2953	494	1.08 (0.89-1.30)	0.425
Q4	2923	448	0.90 (0.74-1.10)	0.291
Q5	2932	445	1.05 (0.85-1.28)	0.670
**TUFA Intake at Dinner**				
Q1	2964	476	1 (Reference)	-
Q2	2900	467	0.97 (0.81-1.16)	0.728
Q3	2964	514	0.92 (0.76-1.12)	0.406
Q4	3030	504	0.91 (0.75-1.09)	0.299
Q5	2975	570	0.94 (0.79-1.13)	0.518
**Female**				
**Daily PUFA Intake**				
Q1	3062	430	1 (Reference)	-
Q2	3008	401	0.97 (0.78-1.22)	0.818
Q3	2992	381	0.84 (0.69-1.03)	0.098
Q4	3093	376	0.80 (0.67-0.95)	0.012
Q5	3148	391	0.71 (0.56-0.89)	0.004
**PUFA Intake at Breakfast**				
Q1	2940	270	1 (Reference)	-
Q2	3315	464	1.30 (1.06-1.59)	0.011
Q3	3180	484	1.37 (1.11-1.70)	0.004
Q4	3017	426	1.23 (1.00-1.52)	0.051
Q5	2851	335	1.09 (0.87-1.36)	0.467
**PUFA Intake at Lunch**				
Q1	2920	410	1 (Reference)	-
Q2	3111	422	1.02 (0.75-1.37)	0.910
Q3	3073	386	1.08 (0.85-1.37)	0.525
Q4	3105	417	0.97 (0.76-1.25)	0.827
Q5	3094	344	0.85 (0.67-1.08)	0.177
**PUFA Intake at Dinner**				
Q1	3063	376	1 (Reference)	-
Q2	3128	379	1.05 (0.86-1.29)	0.632
Q3	3064	410	0.92 (0.75-1.13)	0.426
Q4	2996	398	1.05 (0.88-1.25)	0.603
Q5	3052	416	0.79 (0.65-0.96)	0.017
**Male**				
**Daily PUFA Intake**				
Q1	2965	487	1 (Reference)	-
Q2	3023	492	0.93 (0.78-1.10)	0.387
Q3	3033	504	0.86 (0.71-1.03)	0.108
Q4	2934	501	0.8 (0.67-0.97)	0.020
Q5	2878	547	0.83 (0.71-0.98)	0.024
**PUFA Intake at Breakfast**				

Q1	3088	350	1 (Reference)	-
Q2	2712	499	1.12 (0.93-1.36)	0.230
Q3	2848	533	1.37 (1.13-1.68)	0.002
Q4	3010	572	1.25 (1.03-1.53)	0.027
Q5	3175	577	1.20 (0.97-1.49)	0.093
**PUFA Intake at Lunch**				
Q1	3107	621	1 (Reference)	-
Q2	2918	523	1.14 (0.94-1.37)	0.171
Q3	2953	494	0.92 (0.76-1.11)	0.378
Q4	2923	448	0.91 (0.77-1.08)	0.290
Q5	2932	445	0.91 (0.73-1.12)	0.364
**PUFA Intake at Dinner**				
Q1	2964	476	1 (Reference)	-
Q2	2900	467	0.92 (0.77-1.11)	0.386
Q3	2964	514	0.89 (0.75-1.07)	0.203
Q4	3030	504	0.87 (0.72-1.06)	0.162
Q5	2975	570	0.90 (0.76-1.07)	0.218
**Female**				
**Daily MUFA Intake**				
Q1	3062	430	1 (Reference)	-
Q2	3008	401	0.95 (0.78-1.15)	0.608
Q3	2992	381	0.89 (0.73-1.08)	0.239
Q4	3093	376	0.80 (0.66-0.97)	0.024
Q5	3148	391	0.69 (0.58-0.84)	<0.001
**MUFA Intake at Breakfast**				
Q1	2940	270	1 (Reference)	-
Q2	3315	464	1.28 (1.04-1.57)	0.019
Q3	3180	484	1.36 (1.11-1.67)	0.003
Q4	3017	426	1.26 (1.02-1.55)	0.032
Q5	2851	335	1.12 (0.90-1.40)	0.297
**MUFA Intake at Lunch**				
Q1	2920	410	1 (Reference)	-
Q2	3111	422	1.17 (0.95-1.44)	0.140
Q3	3073	386	1.06 (0.87-1.29)	0.561
Q4	3105	417	1.06 (0.87-1.28)	0.574
Q5	3094	344	0.95 (0.77-1.17)	0.613
**MUFA Intake at Dinner**				
Q1	3063	376	1 (Reference)	-
Q2	3128	379	0.93 (0.76-1.13)	0.447
Q3	3064	410	0.99 (0.79-1.23)	0.894
Q4	2996	398	1.06 (0.87-1.29)	0.561
Q5	3052	416	0.83 (0.67-1.04)	0.104
**Male**				

**Daily MUFA Intake**				
Q1	2965	487	1 (Reference)	-
Q2	3023	492	1.11 (0.90-1.36)	0.333
Q3	3033	504	1.05 (0.85-1.29)	0.652
Q4	2934	501	1.14 (0.94-1.38)	0.173
Q5	2878	547	1.04 (0.85-1.27)	0.721
**MUFA Intake at Breakfast**				
Q1	3088	350	1 (Reference)	-
Q2	2712	499	1.14 (0.93-1.40)	0.199
Q3	2848	533	1.24 (1.01-1.52)	0.044
Q4	3010	572	1.39 (1.15-1.70)	0.001
Q5	3175	577	1.39 (1.14-1.70)	0.002
**MUFA Intake at Lunch**				
Q1	3107	621	1 (Reference)	-
Q2	2918	523	1.06 (0.87-1.29)	0.568
Q3	2953	494	1.08 (0.88-1.32)	0.474
Q4	2923	448	1.03 (0.84-1.26)	0.772
Q5	2932	445	0.94 (0.78-1.14)	0.519
**MUFA Intake at Dinner**				
Q1	2964	476	1 (Reference)	-
Q2	2900	467	1.09 (0.91-1.30)	0.366
Q3	2964	514	0.85 (0.68-1.06)	0.139
Q4	3030	504	0.99 (0.82-1.20)	0.921
Q5	2975	570	0.97 (0.81-1.15)	0.701
		**CVD Mortaility**		
		**Case**	**HR (95% CI)**	**P value**
**Female**				
**Daily TUFA Intake**				
Q1	3062	166	1 (Reference)	-
Q2	3008	171	1.03 (0.78-1.36)	0.815
Q3	2992	161	0.96 (0.72-1.30)	0.805
Q4	3093	153	0.85 (0.63-1.14)	0.270
Q5	3148	157	0.88 (0.66-1.17)	0.383
**TUFA Intake at Breakfast**				
Q1	2940	111	1 (Reference)	-
Q2	3315	191	1.21 (0.90-1.63)	0.200
Q3	3180	199	1.41 (1.07-1.85)	0.016
Q4	3017	162	1.22 (0.89-1.68)	0.207
Q5	2851	145	1.13 (0.79-1.6)	0.505
**TUFA Intake at Lunch**				
Q1	2920	167	1 (Reference)	-
Q2	3111	169	1.24 (0.85-1.81)	0.261
Q3	3073	153	1.17 (0.83-1.64)	0.369
Q4	3105	172	1.18 (0.85-1.65)	0.325

Q5	3094	147	1.06 (0.76-1.47)	0.747
**TUFA Intake at Dinner**				
Q1	3063	146	1 (Reference)	-
Q2	3128	156	1.13 (0.83-1.55)	0.433
Q3	3064	167	1.11 (0.85-1.45)	0.433
Q4	2996	168	1.17 (0.89-1.54)	0.246
Q5	3052	171	0.98 (0.75-1.30)	0.912
**Male**				
**Daily TUFA Intake**				
Q1	2965	188	1 (Reference)	-
Q2	3023	215	1.17 (0.89-1.55)	0.249
Q3	3033	191	1.07 (0.83-1.39)	0.576
Q4	2934	186	1.00 (0.74-1.34)	0.982
Q5	2878	213	1.08 (0.84-1.40)	0.534
**TUFA Intake at Breakfast**				
Q1	3088	135	1 (Reference)	-
Q2	2712	202	1.21 (0.9-1.63)	0.196
Q3	2848	204	1.29 (0.93-1.78)	0.121
Q4	3010	223	1.29 (0.94-1.76)	0.116
Q5	3175	229	1.28 (0.95-1.73)	0.107
**TUFA Intake at Lunch**				
Q1	3107	244	1 (Reference)	-
Q2	2918	209	1.20 (0.84-1.71)	0.310
Q3	2953	196	1.13 (0.82-1.56)	0.441
Q4	2923	173	0.91 (0.67-1.24)	0.544
Q5	2932	171	1.06 (0.75-1.50)	0.724
**TUFA Intake at Dinner**				
Q1	2964	181	1 (Reference)	-
Q2	2900	183	0.99 (0.76-1.29)	0.950
Q3	2964	197	0.93 (0.71-1.23)	0.627
Q4	3030	205	1.02 (0.77-1.33)	0.906
Q5	2975	227	1.15 (0.87-1.53)	0.327
**Female**				
**Daily PUFA Intake**				
Q1	3062	166	1 (Reference)	-
Q2	3008	171	0.97 (0.71-1.33)	0.853
Q3	2992	161	0.82 (0.63-1.08)	0.162
Q4	3093	153	0.93 (0.71-1.21)	0.577
Q5	3148	157	0.84 (0.62-1.13)	0.238
**PUFA Intake at Breakfast**				
Q1	2940	111	1 (Reference)	-
Q2	3315	191	1.22 (0.89-1.68)	0.219
Q3	3180	199	1.43 (1.06-1.94)	0.020
Q4	3017	162	1.28 (0.94-1.74)	0.111

Q5	2851	145	1.14 (0.81-1.62)	0.446
**PUFA Intake at Lunch**				
Q1	2920	167	1 (Reference)	-
Q2	3111	169	1.14 (0.76-1.72)	0.524
Q3	3073	153	1.07 (0.74-1.54)	0.734
Q4	3105	172	1.17 (0.79-1.73)	0.429
Q5	3094	147	1.00 (0.69-1.45)	0.992
**PUFA Intake at Dinner**				
Q1	3063	146	1 (Reference)	-
Q2	3128	156	1.02 (0.73-1.43)	0.911
Q3	3064	167	0.92 (0.66-1.27)	0.600
Q4	2996	168	1.19 (0.92-1.54)	0.178
Q5	3052	171	0.83 (0.62-1.13)	0.234
**Male**				
**Daily PUFA Intake**				
Q1	2965	188	1 (Reference)	-
Q2	3023	215	1.03 (0.84-1.27)	0.786
Q3	3033	191	0.85 (0.65-1.12)	0.245
Q4	2934	186	0.78 (0.61-1)	0.048
Q5	2878	213	0.98 (0.77-1.27)	0.901
**PUFA Intake at Breakfast**				
Q1	3088	135	1 (Reference)	-
Q2	2712	202	1.32 (0.99-1.76)	0.059
Q3	2848	204	1.44 (1.06-1.96)	0.019
Q4	3010	223	1.26 (0.93-1.70)	0.135
Q5	3175	229	1.29 (0.96-1.72)	0.089
**PUFA Intake at Lunch**				
Q1	3107	244	1 (Reference)	-
Q2	2918	209	1.09 (0.78-1.50)	0.616
Q3	2953	196	0.91 (0.68-1.21)	0.515
Q4	2923	173	0.82 (0.59-1.15)	0.250
Q5	2932	171	0.89 (0.65-1.22)	0.469
**PUFA Intake at Dinner**				
Q1	2964	181	1 (Reference)	-
Q2	2900	183	0.90 (0.67-1.20)	0.465
Q3	2964	197	0.92 (0.69-1.22)	0.552
Q4	3030	205	0.90 (0.69-1.18)	0.447
Q5	2975	227	1.03 (0.76-1.38)	0.867
**Female**				
**Daily MUFA Intake**				
Q1	3062	166	1 (Reference)	-
Q2	3008	171	1.27 (0.95-1.70)	0.100
Q3	2992	161	1.15 (0.88-1.50)	0.316

Q4	3093	153	1.02 (0.75-1.40)	0.887
Q5	3148	157	0.95 (0.72-1.26)	0.724
**MUFA Intake at Breakfast**				
Q1	2940	111	1 (Reference)	-
Q2	3315	191	1.28 (0.94-1.74)	0.120
Q3	3180	199	1.48 (1.12-1.96)	0.006
Q4	3017	162	1.31 (0.94-1.82)	0.104
Q5	2851	145	1.23 (0.88-1.72)	0.229
**MUFA Intake at Lunch**				
Q1	2920	167	1 (Reference)	-
Q2	3111	169	1.36 (0.98-1.88)	0.064
Q3	3073	153	1.16 (0.86-1.56)	0.317
Q4	3105	172	1.17 (0.86-1.60)	0.304
Q5	3094	147	1.08 (0.81-1.45)	0.592
**MUFA Intake at Dinner**				
Q1	3063	146	1 (Reference)	-
Q2	3128	156	1.10 (0.80-1.50)	0.552
Q3	3064	167	1.35 (1.01-1.79)	0.040
Q4	2996	168	1.19 (0.91-1.56)	0.191
Q5	3052	171	1.03 (0.79-1.36)	0.806
**Male**				
**Daily MUFA Intake**				
Q1	2965	188	1 (Reference)	-
Q2	3023	215	1.06 (0.79-1.42)	0.699
Q3	3033	191	1.23 (0.92-1.63)	0.157
Q4	2934	186	1.21 (0.92-1.59)	0.179
Q5	2878	213	1.01 (0.77-1.33)	0.919
**MUFA Intake at Breakfast**				
Q1	3088	135	1 (Reference)	-
Q2	2712	202	0.91 (0.66-1.25)	0.566
Q3	2848	204	1.05 (0.75-1.46)	0.777
Q4	3010	223	1.19 (0.88-1.6)	0.249
Q5	3175	229	1.11 (0.81-1.51)	0.509
**MUFA Intake at Lunch**				
Q1	3107	244	1 (Reference)	-
Q2	2918	209	1.04 (0.76-1.43)	0.785
Q3	2953	196	1.19 (0.88-1.62)	0.259
Q4	2923	173	1.04 (0.75-1.45)	0.811
Q5	2932	171	1.04 (0.75-1.44)	0.813
**MUFA Intake at Dinner**				
Q1	2964	181	1 (Reference)	-
Q2	2900	183	1.08 (0.81-1.44)	0.582
Q3	2964	197	0.83 (0.60-1.15)	0.254
Q4	3030	205	1.05 (0.80-1.38)	0.728
Q5	2975	227	1.19 (0.90-1.56)	0.219

		Heart Mortaility		
		**Case**	**HR (95% CI)**	**P value**
**Female**				
**Daily TUFA Intake**				
Q1	3062	98	1 (Reference)	-
Q2	3008	109	1.00 (0.72-1.40)	0.985
Q3	2992	91	0.94 (0.66-1.33)	0.720
Q4	3093	101	0.91 (0.62-1.32)	0.608
Q5	3148	96	0.85 (0.61-1.18)	0.326
**TUFA Intake at Breakfast**				
Q1	2940	65	1 (Reference)	-
Q2	3315	122	1.40 (0.94-2.08)	0.100
Q3	3180	121	1.50 (1.03-2.18)	0.034
Q4	3017	104	1.28 (0.90-1.82)	0.169
Q5	2851	83	1.10 (0.70-1.73)	0.665
**TUFA Intake at Lunch**				
Q1	2920	97	1 (Reference)	-
Q2	3111	98	1.19 (0.72-1.96)	0.504
Q3	3073	91	1.15 (0.72-1.84)	0.550
Q4	3105	106	1.24 (0.76-2.01)	0.389
Q5	3094	103	1.24 (0.82-1.88)	0.302
**TUFA Intake at Dinner**				
Q1	3063	90	1 (Reference)	-
Q2	3128	83	0.99 (0.67-1.45)	0.956
Q3	3064	114	1.16 (0.85-1.59)	0.346
Q4	2996	108	1.2 (0.84-1.71)	0.302
Q5	3052	100	0.85 (0.6-1.2)	0.360
**Male**				
**Daily TUFA Intake**				
Q1	2965	125	1 (Reference)	-
Q2	3023	143	1.23 (0.89-1.71)	0.200
Q3	3033	123	1.08 (0.82-1.43)	0.569
Q4	2934	133	1.05 (0.77-1.45)	0.749
Q5	2878	150	1.09 (0.81-1.47)	0.576
**TUFA Intake at Breakfast**				
Q1	3088	84	1 (Reference)	-
Q2	2712	136	1.51 (1.11-2.04)	0.008
Q3	2848	134	1.51 (1.11-2.04)	0.009
Q4	3010	163	1.64 (1.24-2.17)	0.001
Q5	3175	157	1.51 (1.11-2.06)	0.009
**TUFA Intake at Lunch**				
Q1	3107	171	1 (Reference)	-
Q2	2918	139	1.16 (0.77-1.74)	0.484

Q3	2953	124	1.1 (0.74-1.62)	0.643
Q4	2923	127	0.92 (0.64-1.31)	0.625
Q5	2932	113	1.06 (0.71-1.58)	0.780
**TUFA Intake at Dinner**				
Q1	2964	113	1 (Reference)	-
Q2	2900	124	1.04 (0.75-1.45)	0.808
Q3	2964	143	1.13 (0.8-1.59)	0.485
Q4	3030	145	1.25 (0.9-1.76)	0.185
Q5	2975	149	1.17 (0.82-1.69)	0.384
**Female**				
**Daily PUFA Intake**				
Q1	3062	98	1 (Reference)	-
Q2	3008	109	1.07 (0.72-1.59)	0.720
Q3	2992	91	0.89 (0.64-1.24)	0.487
Q4	3093	101	0.94 (0.66-1.34)	0.723
Q5	3148	96	0.92 (0.64-1.34)	0.675
**PUFA Intake at Breakfast**				
Q1	2940	65	1 (Reference)	-
Q2	3315	122	1.45 (0.97-2.16)	0.069
Q3	3180	121	1.6 (1.1-2.32)	0.014
Q4	3017	104	1.39 (0.92-2.09)	0.115
Q5	2851	83	1.33 (0.88-1.99)	0.171
**PUFA Intake at Lunch**				
Q1	2920	97	1 (Reference)	-
Q2	3111	98	1.18 (0.72-1.91)	0.509
Q3	3073	91	1.13 (0.7-1.84)	0.613
Q4	3105	106	1.25 (0.8-1.97)	0.322
Q5	3094	103	1.2 (0.77-1.87)	0.427
**PUFA Intake at Dinner**				
Q1	3063	90	1 (Reference)	-
Q2	3128	83	1.1 (0.72-1.67)	0.656
Q3	3064	114	0.82 (0.54-1.24)	0.343
Q4	2996	108	1.38 (0.99-1.92)	0.054
Q5	3052	100	0.74 (0.5-1.08)	0.115
**Male**				
**Daily PUFA Intake**				
Q1	2965	125	1 (Reference)	-
Q2	3023	143	0.98 (0.74-1.29)	0.860
Q3	3033	123	0.94 (0.7-1.28)	0.708
Q4	2934	133	0.74 (0.54-1.02)	0.062
Q5	2878	150	0.98 (0.69-1.39)	0.923
**PUFA Intake at Breakfast**				
Q1	3088	84	1 (Reference)	-
Q2	2712	136	1.48 (1-2.18)	0.048

Q3	2848	134	1.6 (1.13-2.26)	0.008
Q4	3010	163	1.32 (0.95-1.86)	0.101
Q5	3175	157	1.54 (1.07-2.21)	0.021
**PUFA Intake at Lunch**				
Q1	3107	171	1 (Reference)	-
Q2	2918	139	0.97 (0.66-1.42)	0.866
Q3	2953	124	0.85 (0.6-1.22)	0.385
Q4	2923	127	0.84 (0.58-1.23)	0.377
Q5	2932	113	0.81 (0.58-1.13)	0.209
**PUFA Intake at Dinner**				
Q1	2964	113	1 (Reference)	-
Q2	2900	124	1.1 (0.78-1.54)	0.601
Q3	2964	143	1.02 (0.74-1.41)	0.880
Q4	3030	145	1.07 (0.78-1.48)	0.662
Q5	2975	149	1.11 (0.77-1.59)	0.571
**Female**				
**Daily MUFA Intake**				
Q1	3062	98	1 (Reference)	-
Q2	3008	109	1.37 (0.95-1.97)	0.091
Q3	2992	91	1.2 (0.87-1.65)	0.261
Q4	3093	101	0.96 (0.66-1.39)	0.825
Q5	3148	96	1.09 (0.76-1.55)	0.649
**MUFA Intake at Breakfast**				
Q1	2940	65	1 (Reference)	-
Q2	3315	122	1.39 (0.98-1.97)	0.065
Q3	3180	121	1.5 (1.07-2.1)	0.020
Q4	3017	104	1.31 (0.92-1.86)	0.133
Q5	2851	83	1.14 (0.75-1.74)	0.531
**MUFA Intake at Lunch**				
Q1	2920	97	1 (Reference)	-
Q2	3111	98	1.32 (0.83-2.1)	0.246
Q3	3073	91	1.21 (0.77-1.92)	0.410
Q4	3105	106	1.29 (0.83-2)	0.255
Q5	3094	103	1.26 (0.87-1.85)	0.221
**MUFA Intake at Dinner**				
Q1	3063	90	1 (Reference)	-
Q2	3128	83	1.24 (0.84-1.83)	0.277
Q3	3064	114	1.41 (1.02-1.96)	0.038
Q4	2996	108	1.32 (0.95-1.83)	0.100
Q5	3052	100	0.97 (0.67-1.42)	0.886
**Male**				
**Daily MUFA Intake**				
Q1	2965	125	1 (Reference)	-

Q2	3023	143	1.16 (0.85-1.59)	0.345
Q3	3033	123	1.25 (0.9-1.72)	0.181
Q4	2934	133	1.25 (0.92-1.69)	0.156
Q5	2878	150	1.05 (0.78-1.41)	0.749
**MUFA Intake at Breakfast**				
Q1	3088	84	1 (Reference)	-
Q2	2712	136	1.06 (0.74-1.53)	0.743
Q3	2848	134	1.19 (0.86-1.66)	0.290
Q4	3010	163	1.49 (1.08-2.06)	0.017
Q5	3175	157	1.19 (0.86-1.64)	0.300
**MUFA Intake at Lunch**				
Q1	3107	171	1 (Reference)	-
Q2	2918	139	1.1 (0.79-1.52)	0.566
Q3	2953	124	1.14 (0.77-1.69)	0.512
Q4	2923	127	1.07 (0.73-1.55)	0.735
Q5	2932	113	1.11 (0.76-1.61)	0.593
**MUFA Intake at Dinner**				
Q1	2964	113	1 (Reference)	-
Q2	2900	124	1 (0.7-1.42)	0.979
Q3	2964	143	1.05 (0.71-1.55)	0.820
Q4	3030	145	1.2 (0.84-1.71)	0.310
Q5	2975	149	1.15 (0.81-1.65)	0.432
		**Hypertension Mortaility**		
		**Case**	**HR (95% CI)**	**P value**
**Female**				
**Daily TUFA Intake**				
Q1	3062	75	1 (Reference)	-
Q2	3008	74	1.02 (0.67-1.55)	0.922
Q3	2992	68	0.8 (0.55-1.15)	0.219
Q4	3093	56	0.63 (0.41-0.97)	0.035
Q5	3148	65	0.92 (0.56-1.5)	0.724
**TUFA Intake at Breakfast**				
Q1	2940	46	1 (Reference)	-
Q2	3315	79	0.94 (0.62-1.44)	0.782
Q3	3180	87	1.35 (0.84-2.18)	0.211
Q4	3017	64	1.03 (0.58-1.81)	0.927
Q5	2851	62	0.98 (0.6-1.58)	0.921
**TUFA Intake at Lunch**				
Q1	2920	69	1 (Reference)	-
Q2	3111	78	1.5 (0.88-2.55)	0.134
Q3	3073	57	1.31 (0.78-2.18)	0.301
Q4	3105	77	1.57 (0.97-2.55)	0.066
Q5	3094	57	1.13 (0.64-1.99)	0.679
**TUFA Intake at Dinner**				

Q1	3063	63	1 (Reference)	-
Q2	3128	76	1.24 (0.8-1.94)	0.330
Q3	3064	65	0.95 (0.61-1.48)	0.827
Q4	2996	64	1.03 (0.71-1.51)	0.860
Q5	3052	70	1.07 (0.7-1.65)	0.752
**Male**				
**Daily TUFA Intake**				
Q1	2965	72	1 (Reference)	-
Q2	3023	71	1.09 (0.65-1.83)	0.739
Q3	3033	75	1.04 (0.66-1.63)	0.866
Q4	2934	72	0.99 (0.58-1.7)	0.974
Q5	2878	74	1.14 (0.75-1.73)	0.525
**TUFA Intake at Breakfast**				
Q1	3088	50	1 (Reference)	-
Q2	2712	77	0.99 (0.62-1.59)	0.969
Q3	2848	75	1.12 (0.64-1.97)	0.680
Q4	3010	82	1.03 (0.59-1.8)	0.919
Q5	3175	80	0.98 (0.58-1.63)	0.924
**TUFA Intake at Lunch**				
Q1	3107	76	1 (Reference)	-
Q2	2918	81	1.41 (0.72-2.72)	0.310
Q3	2953	77	1.2 (0.68-2.13)	0.521
Q4	2923	69	1.13 (0.63-2.02)	0.674
Q5	2932	61	1.12 (0.58-2.16)	0.735
**TUFA Intake at Dinner**				
Q1	2964	69	1 (Reference)	-
Q2	2900	71	0.99 (0.63-1.56)	0.965
Q3	2964	70	0.85 (0.55-1.31)	0.449
Q4	3030	70	0.8 (0.49-1.31)	0.372
Q5	2975	84	1.2 (0.79-1.82)	0.394
**Female**				
**Daily PUFA Intake**				
Q1	3062	75	1 (Reference)	-
Q2	3008	74	0.89 (0.58-1.37)	0.608
Q3	2992	68	0.75 (0.5-1.12)	0.157
Q4	3093	56	0.91 (0.59-1.39)	0.657
Q5	3148	65	0.86 (0.54-1.35)	0.499
**PUFA Intake at Breakfast**				
Q1	2940	46	1 (Reference)	-
Q2	3315	79	0.79 (0.52-1.21)	0.273
Q3	3180	87	1.17 (0.72-1.89)	0.519
Q4	3017	64	0.86 (0.53-1.37)	0.519
Q5	2851	62	0.85 (0.53-1.37)	0.506
**PUFA Intake at Lunch**				

Q1	2920	69	1 (Reference)	-
Q2	3111	78	1.05 (0.55-1.99)	0.884
Q3	3073	57	1.23 (0.64-2.36)	0.539
Q4	3105	77	1.23 (0.63-2.41)	0.532
Q5	3094	57	1.08 (0.55-2.1)	0.823
**PUFA Intake at Dinner**				
Q1	3063	63	1 (Reference)	-
Q2	3128	76	1.13 (0.78-1.63)	0.519
Q3	3064	65	1 (0.6-1.68)	0.999
Q4	2996	64	1.03 (0.67-1.57)	0.904
Q5	3052	70	0.9 (0.57-1.44)	0.669
**Male**				
**Daily PUFA Intake**				
Q1	2965	72	1 (Reference)	-
Q2	3023	71	1.1 (0.76-1.58)	0.611
Q3	3033	75	0.79 (0.51-1.23)	0.296
Q4	2934	72	0.75 (0.48-1.16)	0.195
Q5	2878	74	1 (0.65-1.55)	0.984
**PUFA Intake at Breakfast**				
Q1	3088	50	1 (Reference)	-
Q2	2712	77	1.16 (0.75-1.79)	0.506
Q3	2848	75	1.43 (0.85-2.41)	0.172
Q4	3010	82	1.18 (0.72-1.92)	0.514
Q5	3175	80	0.88 (0.48-1.63)	0.688
**PUFA Intake at Lunch**				
Q1	3107	76	1 (Reference)	-
Q2	2918	81	1.34 (0.76-2.37)	0.308
Q3	2953	77	0.94 (0.51-1.72)	0.828
Q4	2923	69	0.89 (0.5-1.57)	0.679
Q5	2932	61	0.99 (0.54-1.83)	0.982
**PUFA Intake at Dinner**				
Q1	2964	69	1 (Reference)	-
Q2	2900	71	0.75 (0.49-1.13)	0.164
Q3	2964	70	0.81 (0.51-1.3)	0.386
Q4	3030	70	0.66 (0.46-0.95)	0.026
Q5	2975	84	0.97 (0.64-1.47)	0.892
**Female**				
**Daily MUFA Intake**				
Q1	3062	75	1 (Reference)	-
Q2	3008	74	1.31 (0.93-1.83)	0.116
Q3	2992	68	1.14 (0.73-1.77)	0.558
Q4	3093	56	0.92 (0.57-1.5)	0.743
Q5	3148	65	0.76 (0.5-1.17)	0.208

**MUFA Intake at Breakfast**				
Q1	2940	46	1 (Reference)	-
Q2	3315	79	1.16 (0.73-1.83)	0.535
Q3	3180	87	1.61 (0.98-2.63)	0.059
Q4	3017	64	1.34 (0.74-2.44)	0.328
Q5	2851	62	1.02 (0.62-1.67)	0.938
**MUFA Intake at Lunch**				
Q1	2920	69	1 (Reference)	-
Q2	3111	78	1.55 (0.94-2.57)	0.085
Q3	3073	57	1.41 (0.87-2.28)	0.158
Q4	3105	77	1.32 (0.82-2.13)	0.245
Q5	3094	57	1.15 (0.69-1.92)	0.587
**MUFA Intake at Dinner**				
Q1	3063	63	1 (Reference)	-
Q2	3128	76	0.8 (0.51-1.24)	0.319
Q3	3064	65	1.05 (0.66-1.66)	0.846
Q4	2996	64	0.99 (0.71-1.37)	0.932
Q5	3052	70	0.91 (0.59-1.41)	0.671
**Male**				
**Daily MUFA Intake**				
Q1	2965	72	1 (Reference)	-
Q2	3023	71	0.84 (0.52-1.36)	0.476
Q3	3033	75	1.1 (0.69-1.76)	0.674
Q4	2934	72	1.33 (0.83-2.12)	0.229
Q5	2878	74	1.04 (0.68-1.59)	0.854
**MUFA Intake at Breakfast**				
Q1	3088	50	1 (Reference)	-
Q2	2712	77	0.77 (0.46-1.3)	0.330
Q3	2848	75	0.88 (0.48-1.62)	0.675
Q4	3010	82	1.03 (0.58-1.84)	0.919
Q5	3175	80	0.96 (0.56-1.66)	0.897
**MUFA Intake at Lunch**				
Q1	3107	76	1 (Reference)	-
Q2	2918	81	1.03 (0.56-1.86)	0.933
Q3	2953	77	1.12 (0.66-1.92)	0.664
Q4	2923	69	1.18 (0.66-2.09)	0.574
Q5	2932	61	1.13 (0.63-2.02)	0.685
**MUFA Intake at Dinner**				
Q1	2964	69	1 (Reference)	-
Q2	2900	71	0.97 (0.61-1.53)	0.889
Q3	2964	70	0.6 (0.38-0.94)	0.025
Q4	3030	70	0.81 (0.5-1.29)	0.366
Q5	2975	84	1.19 (0.78-1.83)	0.418

Supplementary Table S9. Adjusted hazard ratios for mortalities stratified by age group across quintiles of dietary UFAs intake in all-day and three meals.

Variable	N	All-cause Mortaility		
		Case	HR (95% CI)	P value
**Age≤60**				
**Daily TUFA Intake**				
Q1	4154	174	1 (Reference)	-
Q2	4146	163	0.96 (0.7-1.31)	0.790
Q3	4138	151	0.82 (0.57-1.18)	0.279
Q4	4084	168	0.77 (0.55-1.09)	0.140
Q5	3926	166	0.72 (0.53-0.99)	0.042
**TUFA Intake at Breakfast**				
Q1	4691	177	1 (Reference)	-
Q2	3905	122	1.04 (0.73-1.48)	0.835
Q3	3920	162	1.22 (0.87-1.71)	0.243
Q4	3914	189	1.41 (1.02-1.94)	0.036
Q5	4018	172	1.09 (0.81-1.45)	0.568
**TUFA Intake at Lunch**				
Q1	3863	193	1 (Reference)	-
Q2	3974	174	1.18 (0.85-1.65)	0.323
Q3	4115	149	0.93 (0.65-1.31)	0.660
Q4	4191	144	0.73 (0.53-1)	0.048
Q5	4305	162	0.77 (0.54-1.09)	0.140
**TUFA Intake at Dinner**				
Q1	4123	157	1 (Reference)	-
Q2	4184	158	1.07 (0.76-1.5)	0.688
Q3	4097	150	1.09 (0.79-1.5)	0.582
Q4	4032	174	1.22 (0.91-1.63)	0.176
Q5	4012	183	1.03 (0.77-1.38)	0.846
**Age>60**				
**Daily TUFA Intake**				
Q1	1873	743	1 (Reference)	-
Q2	1885	730	0.94 (0.83-1.05)	0.267
Q3	1887	734	0.91 (0.8-1.04)	0.166
Q4	1943	709	0.85 (0.73-0.98)	0.028
Q5	2100	772	0.88 (0.78-0.99)	0.038
**TUFA Intake at Breakfast**				
Q1	1337	443	1 (Reference)	-
Q2	2122	841	1.16 (0.99-1.36)	0.073
Q3	2108	855	1.33 (1.14-1.54)	<0.001
Q4	2113	809	1.2 (1.03-1.4)	0.019
Q5	2008	740	1.25 (1.05-1.49)	0.012
**TUFA Intake at Lunch**				
Q1	2164	838	1 (Reference)	-

Q2	2055	771	1.11 (0.96-1.28)	0.174
Q3	1911	731	1.07 (0.92-1.24)	0.380
Q4	1837	721	1.05 (0.9-1.23)	0.528
Q5	1721	627	1.08 (0.94-1.24)	0.257
**TUFA Intake at Dinner**				
Q1	1904	695	1 (Reference)	-
Q2	1844	688	0.96 (0.82-1.13)	0.648
Q3	1931	774	0.93 (0.81-1.07)	0.300
Q4	1994	728	0.89 (0.78-1.03)	0.118
Q5	2015	803	0.88 (0.76-1.01)	0.069
**Age≤60**				
**Daily PUFA Intake**				
Q1	4213	205	1 (Reference)	-
Q2	4161	173	0.98 (0.7-1.37)	0.907
Q3	4073	147	0.83 (0.59-1.18)	0.303
Q4	4036	132	0.63 (0.45-0.88)	0.007
Q5	3965	165	0.76 (0.55-1.06)	0.105
**PUFA Intake at Breakfast**				
Q1	4796	184	1 (Reference)	-
Q2	3961	129	0.96 (0.69-1.34)	0.820
Q3	3888	175	1.4 (0.98-2)	0.066
Q4	3894	172	1.07 (0.75-1.53)	0.701
Q5	3909	162	1.05 (0.78-1.41)	0.735
**PUFA Intake at Lunch**				
Q1	3858	208	1 (Reference)	-
Q2	4028	173	0.96 (0.66-1.39)	0.823
Q3	4134	141	0.8 (0.57-1.12)	0.187
Q4	4214	165	0.78 (0.57-1.07)	0.125
Q5	4214	138	0.6 (0.41-0.88)	0.010
**PUFA Intake at Dinner**				
Q1	4140	179	1 (Reference)	-
Q2	4213	150	1.16 (0.84-1.6)	0.354
Q3	4117	159	1.05 (0.78-1.41)	0.758
Q4	4057	173	1.16 (0.89-1.52)	0.257
Q5	3921	161	1.01 (0.77-1.32)	0.958
**Age>60**				
**Daily PUFA Intake**				
Q1	1813	786	1 (Reference)	-
Q2	1874	737	0.9 (0.8-1.03)	0.121
Q3	1952	749	0.85 (0.75-0.96)	0.013
Q4	1990	748	0.87 (0.77-1)	0.043
Q5	2059	668	0.77 (0.66-0.9)	0.001
**PUFA Intake at Breakfast**				
Q1	1236	418	1 (Reference)	-

Q2	2068	831	1.13 (0.98-1.31)	0.087
Q3	2138	876	1.24 (1.07-1.45)	0.006
Q4	2131	812	1.18 (1.04-1.35)	0.014
Q5	2115	751	1.13 (0.97-1.33)	0.112
**PUFA Intake at Lunch**				
Q1	2167	860	1 (Reference)	-
Q2	2000	788	1.07 (0.9-1.26)	0.450
Q3	1897	734	1.05 (0.92-1.21)	0.467
Q4	1810	668	0.99 (0.84-1.16)	0.910
Q5	1814	638	1.01 (0.88-1.15)	0.934
**PUFA Intake at Dinner**				
Q1	1890	736	1 (Reference)	-
Q2	1815	701	0.98 (0.85-1.12)	0.734
Q3	1910	724	0.94 (0.81-1.09)	0.376
Q4	1972	748	0.91 (0.79-1.05)	0.188
Q5	2101	779	0.84 (0.73-0.96)	0.011
**Age≤60**				
**Daily MUFA Intake**				
Q1	4096	149	1 (Reference)	-
Q2	4134	168	1.12 (0.8-1.57)	0.513
Q3	4140	151	0.89 (0.63-1.26)	0.515
Q4	4072	175	1.01 (0.71-1.43)	0.966
Q5	4006	179	0.83 (0.6-1.15)	0.263
**MUFA Intake at Breakfast**				
Q1	4616	177	1 (Reference)	-
Q2	3901	132	1.04 (0.74-1.45)	0.831
Q3	3914	144	0.99 (0.74-1.32)	0.937
Q4	3955	189	1.33 (0.99-1.78)	0.062
Q5	4062	180	1.09 (0.82-1.46)	0.542
**MUFA Intake at Lunch**				
Q1	3822	187	1 (Reference)	-
Q2	3999	167	1.06 (0.77-1.45)	0.719
Q3	4133	148	0.88 (0.64-1.21)	0.429
Q4	4133	154	0.77 (0.55-1.07)	0.112
Q5	4361	166	0.71 (0.5-1.02)	0.063
**MUFA Intake at Dinner**				
Q1	4095	152	1 (Reference)	-
Q2	4150	155	1.2 (0.89-1.6)	0.230
Q3	4061	139	0.92 (0.65-1.3)	0.648
Q4	4118	189	1.38 (1.06-1.8)	0.019
Q5	4024	187	1.05 (0.78-1.4)	0.754
**Age>60**				
**Daily MUFA Intake**				

Q1	1930	716	1 (Reference)	-
Q2	1894	707	1.02 (0.89-1.17)	0.735
Q3	1887	716	1 (0.87-1.15)	0.982
Q4	1957	756	0.99 (0.87-1.13)	0.891
Q5	2020	793	0.93 (0.82-1.05)	0.258
**MUFA Intake at Breakfast**				
Q1	1412	468	1 (Reference)	-
Q2	2130	833	1.17 (1.01-1.36)	0.033
Q3	2113	852	1.3 (1.13-1.5)	0.000
Q4	2071	791	1.24 (1.07-1.43)	0.005
Q5	1962	754	1.36 (1.14-1.61)	0.001
**MUFA Intake at Lunch**				
Q1	2204	844	1 (Reference)	-
Q2	2029	735	1.11 (0.95-1.31)	0.190
Q3	1896	723	1.08 (0.92-1.26)	0.335
Q4	1895	748	1.15 (0.99-1.35)	0.071
Q5	1664	638	1.06 (0.92-1.21)	0.443
**MUFA Intake at Dinner**				
Q1	1934	692	1 (Reference)	-
Q2	1878	693	0.9 (0.77-1.05)	0.182
Q3	1964	737	0.93 (0.8-1.08)	0.335
Q4	1811	759	0.94 (0.81-1.09)	0.396
Q5	2001	807	0.87 (0.75-1.01)	0.070
		**CVD Mortaility**		
		**Case**	**HR (95% CI)**	**P value**
**Age≤60**				
**Daily TUFA Intake**				
Q1	4154	49	1 (Reference)	-
Q2	4146	42	1.13 (0.66-1.93)	0.645
Q3	4138	51	1.26 (0.7-2.3)	0.437
Q4	4084	48	1.11 (0.65-1.89)	0.694
Q5	3926	58	1.24 (0.76-2.02)	0.386
**TUFA Intake at Breakfast**				
Q1	4691	60	1 (Reference)	-
Q2	3905	34	0.85 (0.42-1.73)	0.648
Q3	3920	47	1.03 (0.58-1.82)	0.918
Q4	3914	50	1.29 (0.7-2.38)	0.407
Q5	4018	57	0.98 (0.57-1.68)	0.931
**TUFA Intake at Lunch**				
Q1	3863	57	1 (Reference)	-
Q2	3974	48	1.12 (0.62-2.03)	0.700
Q3	4115	40	0.82 (0.44-1.54)	0.540
Q4	4191	47	0.85 (0.5-1.45)	0.552
Q5	4305	56	0.87 (0.5-1.53)	0.630

**TUFA Intake at Dinner**				
Q1	4123	44	1 (Reference)	-
Q2	4184	43	0.79 (0.46-1.36)	0.397
Q3	4097	50	1.14 (0.64-2.05)	0.651
Q4	4032	51	1.24 (0.79-1.96)	0.348
Q5	4012	60	1.36 (0.82-2.26)	0.236
**Age>60**				
**Daily TUFA Intake**				
Q1	1873	305	1 (Reference)	-
Q2	1885	344	1.14 (0.92-1.4)	0.222
Q3	1887	301	0.97 (0.79-1.18)	0.744
Q4	1943	291	0.89 (0.71-1.11)	0.306
Q5	2100	312	1 (0.8-1.25)	0.995
**TUFA Intake at Breakfast**				
Q1	1337	186	1 (Reference)	-
Q2	2122	359	1.16 (0.92-1.46)	0.209
Q3	2108	356	1.32 (1.05-1.68)	0.020
Q4	2113	335	1.17 (0.93-1.46)	0.175
Q5	2008	317	1.28 (0.98-1.66)	0.071
**TUFA Intake at Lunch**				
Q1	2164	354	1 (Reference)	-
Q2	2055	330	1.17 (0.91-1.51)	0.225
Q3	1911	309	1.15 (0.9-1.47)	0.257
Q4	1837	298	1.05 (0.84-1.32)	0.666
Q5	1721	262	1.12 (0.88-1.41)	0.357
**TUFA Intake at Dinner**				
Q1	1904	283	1 (Reference)	-
Q2	1844	296	1.14 (0.9-1.45)	0.282
Q3	1931	314	1.04 (0.87-1.24)	0.661
Q4	1994	322	1.11 (0.9-1.37)	0.309
Q5	2015	338	1.03 (0.84-1.26)	0.767
**Age≤60**				
**Daily PUFA Intake**				
Q1	4213	61	1 (Reference)	-
Q2	4161	53	1.03 (0.64-1.68)	0.896
Q3	4073	39	0.96 (0.55-1.69)	0.893
Q4	4036	38	0.72 (0.36-1.45)	0.352
Q5	3965	57	1.22 (0.74-2.01)	0.430
**PUFA Intake at Breakfast**				
Q1	4796	59	1 (Reference)	-
Q2	3961	37	0.91 (0.52-1.58)	0.726
Q3	3888	52	1.53 (0.91-2.6)	0.109
Q4	3894	51	1.17 (0.64-2.14)	0.599
Q5	3909	49	0.98 (0.61-1.56)	0.930

**PUFA Intake at Lunch**				
Q1	3858	59	1 (Reference)	-
Q2	4028	53	0.84 (0.41-1.72)	0.626
Q3	4134	37	0.65 (0.33-1.3)	0.222
Q4	4214	51	0.85 (0.44-1.65)	0.633
Q5	4214	48	0.67 (0.34-1.32)	0.240
**PUFA Intake at Dinner**				
Q1	4140	49	1 (Reference)	-
Q2	4213	49	0.99 (0.6-1.64)	0.968
Q3	4117	49	1.21 (0.76-1.94)	0.412
Q4	4057	52	1.28 (0.79-2.06)	0.309
Q5	3921	49	1.39 (0.88-2.21)	0.159
**Age>60**				
**Daily PUFA Intake**				
Q1	1813	337	1 (Reference)	-
Q2	1874	327	0.95 (0.78-1.16)	0.606
Q3	1952	308	0.8 (0.64-0.99)	0.040
Q4	1990	306	0.88 (0.75-1.04)	0.141
Q5	2059	275	0.83 (0.67-1.03)	0.085
**PUFA Intake at Breakfast**				
Q1	1236	167	1 (Reference)	-
Q2	2068	355	1.15 (0.93-1.43)	0.192
Q3	2138	377	1.29 (1.03-1.6)	0.024
Q4	2131	329	1.15 (0.95-1.41)	0.153
Q5	2115	325	1.23 (0.98-1.54)	0.073
**PUFA Intake at Lunch**				
Q1	2167	362	1 (Reference)	-
Q2	2000	330	1.11 (0.88-1.39)	0.368
Q3	1897	319	1.04 (0.84-1.28)	0.708
Q4	1810	275	0.99 (0.76-1.29)	0.942
Q5	1814	267	1.02 (0.83-1.25)	0.850
**PUFA Intake at Dinner**				
Q1	1890	313	1 (Reference)	-
Q2	1815	295	1 (0.77-1.3)	0.990
Q3	1910	289	0.95 (0.76-1.19)	0.638
Q4	1972	326	1.01 (0.8-1.28)	0.939
Q5	2101	330	0.89 (0.71-1.12)	0.316
**Age≤60**				
**Daily MUFA Intake**				
Q1	4096	43	1 (Reference)	-
Q2	4134	43	1.25 (0.65-2.41)	0.502
Q3	4140	42	1.44 (0.84-2.48)	0.182
Q4	4072	59	1.52 (0.87-2.64)	0.138

Q5	4006	61	1.27 (0.73-2.19)	0.395
**MUFA Intake at Breakfast**				
Q1	4616	65	1 (Reference)	-
Q2	3901	33	0.69 (0.36-1.32)	0.261
Q3	3914	36	0.56 (0.32-0.99)	0.048
Q4	3955	57	1.32 (0.77-2.27)	0.304
Q5	4062	57	0.9 (0.53-1.53)	0.699
**MUFA Intake at Lunch**				
Q1	3822	55	1 (Reference)	-
Q2	3999	42	0.82 (0.43-1.58)	0.557
Q3	4133	45	0.83 (0.48-1.42)	0.482
Q4	4133	51	0.89 (0.5-1.57)	0.683
Q5	4361	55	0.76 (0.43-1.33)	0.334
**MUFA Intake at Dinner**				
Q1	4095	44	1 (Reference)	-
Q2	4150	41	1.05 (0.61-1.79)	0.863
Q3	4061	44	1.03 (0.55-1.93)	0.933
Q4	4118	56	1.44 (0.86-2.41)	0.167
Q5	4024	63	1.49 (0.92-2.41)	0.106
**Age>60**				
**Daily MUFA Intake**				
Q1	1930	290	1 (Reference)	-
Q2	1894	313	1.21 (0.98-1.48)	0.070
Q3	1887	316	1.16 (0.95-1.43)	0.143
Q4	1957	324	1.12 (0.9-1.39)	0.310
Q5	2020	310	1.01 (0.82-1.26)	0.891
**MUFA Intake at Breakfast**				
Q1	1412	197	1 (Reference)	-
Q2	2130	343	1.12 (0.9-1.41)	0.311
Q3	2113	376	1.37 (1.09-1.71)	0.007
Q4	2071	317	1.16 (0.92-1.47)	0.207
Q5	1962	320	1.31 (1.03-1.69)	0.031
**MUFA Intake at Lunch**				
Q1	2204	355	1 (Reference)	-
Q2	2029	323	1.27 (1.01-1.6)	0.042
Q3	1896	307	1.18 (0.94-1.49)	0.150
Q4	1895	297	1.13 (0.9-1.43)	0.295
Q5	1664	271	1.17 (0.94-1.44)	0.153
**MUFA Intake at Dinner**				
Q1	1934	283	1 (Reference)	-
Q2	1878	290	1.03 (0.85-1.26)	0.739
Q3	1964	306	1.1 (0.91-1.34)	0.305
Q4	1811	324	1.09 (0.88-1.33)	0.424
Q5	2001	350	1.05 (0.87-1.26)	0.602

		Heart Mortaility		
		Case	HR (95% CI)	P value
**Age≤60**				
**Daily TUFA Intake**				
Q1	4154	30	1 (Reference)	-
Q2	4146	30	1.46 (0.74-2.88)	0.265
Q3	4138	35	1.63 (0.8-3.32)	0.176
Q4	4084	32	1.18 (0.59-2.34)	0.639
Q5	3926	43	1.44 (0.81-2.55)	0.207
**TUFA Intake at Breakfast**				
Q1	4691	39	1 (Reference)	-
Q2	3905	21	1.1 (0.45-2.66)	0.830
Q3	3920	34	1.3 (0.72-2.36)	0.381
Q4	3914	37	1.41 (0.75-2.68)	0.285
Q5	4018	39	1.05 (0.62-1.79)	0.848
**TUFA Intake at Lunch**				
Q1	3863	38	1 (Reference)	-
Q2	3974	31	0.94 (0.48-1.84)	0.853
Q3	4115	27	0.9 (0.45-1.78)	0.756
Q4	4191	31	0.76 (0.4-1.47)	0.413
Q5	4305	43	1.05 (0.57-1.94)	0.884
**TUFA Intake at Dinner**				
Q1	4123	28	1 (Reference)	-
Q2	4184	26	0.67 (0.33-1.36)	0.268
Q3	4097	40	1.52 (0.72-3.22)	0.269
Q4	4032	39	1.69 (0.89-3.19)	0.108
Q5	4012	37	1.36 (0.71-2.59)	0.347
**Age>60**				
**Daily TUFA Intake**				
Q1	1873	193	1 (Reference)	-
Q2	1885	222	1.1 (0.86-1.41)	0.455
Q3	1887	179	0.91 (0.72-1.15)	0.417
Q4	1943	202	0.96 (0.73-1.28)	0.797
Q5	2100	203	0.97 (0.72-1.3)	0.835
**TUFA Intake at Breakfast**				
Q1	1337	110	1 (Reference)	-
Q2	2122	237	1.36 (1.02-1.81)	0.033
Q3	2108	221	1.42 (1.05-1.92)	0.022
Q4	2113	230	1.36 (1.05-1.75)	0.020
Q5	2008	201	1.38 (1.01-1.89)	0.041
**TUFA Intake at Lunch**				
Q1	2164	230	1 (Reference)	-
Q2	2055	206	1.16 (0.81-1.68)	0.410
Q3	1911	188	1.11 (0.8-1.52)	0.535

Q4	1837	202	1.12 (0.78-1.59)	0.539
Q5	1721	173	1.17 (0.85-1.62)	0.335
**TUFA Intake at Dinner**				
Q1	1904	175	1 (Reference)	-
Q2	1844	181	1.1 (0.82-1.48)	0.507
Q3	1931	217	1.12 (0.89-1.42)	0.316
Q4	1994	214	1.19 (0.93-1.53)	0.158
Q5	2015	212	0.99 (0.76-1.28)	0.909
**Age≤60**				
**Daily PUFA Intake**				
Q1	4213	44	1 (Reference)	-
Q2	4161	30	0.82 (0.46-1.49)	0.516
Q3	4073	26	1.07 (0.55-2.08)	0.845
Q4	4036	27	0.74 (0.31-1.75)	0.494
Q5	3965	43	1.19 (0.65-2.16)	0.569
**PUFA Intake at Breakfast**				
Q1	4796	40	1 (Reference)	-
Q2	3961	23	1.04 (0.5-2.18)	0.912
Q3	3888	35	1.71 (0.88-3.32)	0.113
Q4	3894	35	0.96 (0.52-1.77)	0.887
Q5	3909	37	1.14 (0.71-1.82)	0.595
**PUFA Intake at Lunch**				
Q1	3858	41	1 (Reference)	-
Q2	4028	34	0.8 (0.39-1.61)	0.519
Q3	4134	23	0.73 (0.34-1.6)	0.433
Q4	4214	37	1.03 (0.52-2.04)	0.941
Q5	4214	35	0.83 (0.43-1.61)	0.574
**PUFA Intake at Dinner**				
Q1	4140	27	1 (Reference)	-
Q2	4213	38	1.53 (0.73-3.22)	0.261
Q3	4117	37	1.73 (0.86-3.46)	0.123
Q4	4057	37	2.02 (1.03-3.96)	0.040
Q5	3921	31	1.82 (0.94-3.5)	0.074
**Age>60**				
**Daily PUFA Intake**				
Q1	1813	218	1 (Reference)	-
Q2	1874	210	1.02 (0.81-1.29)	0.857
Q3	1952	198	0.87 (0.68-1.11)	0.251
Q4	1990	192	0.87 (0.7-1.07)	0.176
Q5	2059	181	0.89 (0.67-1.2)	0.449
**PUFA Intake at Breakfast**				
Q1	1236	97	1 (Reference)	-
Q2	2068	229	1.38 (1.07-1.77)	0.013
Q3	2138	243	1.47 (1.14-1.89)	0.004

Q4	2131	213	1.34 (1.01-1.78)	0.042
Q5	2115	217	1.51 (1.14-1.98)	0.004
**PUFA Intake at Lunch**				
Q1	2167	239	1 (Reference)	-
Q2	2000	203	1.06 (0.79-1.43)	0.682
Q3	1897	203	1.02 (0.74-1.39)	0.913
Q4	1810	183	1 (0.73-1.37)	0.998
Q5	1814	171	1.03 (0.75-1.4)	0.870
**PUFA Intake at Dinner**				
Q1	1890	194	1 (Reference)	-
Q2	1815	198	1.08 (0.8-1.45)	0.602
Q3	1910	179	0.9 (0.7-1.15)	0.387
Q4	1972	221	1.13 (0.85-1.49)	0.407
Q5	2101	207	0.84 (0.65-1.09)	0.195
**Age≤60**				
**Daily MUFA Intake**				
Q1	4096	23	1 (Reference)	-
Q2	4134	32	2.1 (0.92-4.79)	0.077
Q3	4140	32	2.03 (1.05-3.93)	0.035
Q4	4072	37	1.85 (0.89-3.86)	0.100
Q5	4006	46	2.01 (1.04-3.85)	0.037
**MUFA Intake at Breakfast**				
Q1	4616	42	1 (Reference)	-
Q2	3901	23	0.99 (0.47-2.09)	0.974
Q3	3914	24	0.56 (0.29-1.05)	0.072
Q4	3955	46	1.83 (1.05-3.19)	0.034
Q5	4062	35	0.82 (0.5-1.36)	0.444
**MUFA Intake at Lunch**				
Q1	3822	36	1 (Reference)	-
Q2	3999	26	0.85 (0.41-1.75)	0.649
Q3	4133	33	0.95 (0.48-1.86)	0.875
Q4	4133	34	1.01 (0.57-1.79)	0.984
Q5	4361	41	0.98 (0.54-1.76)	0.936
**MUFA Intake at Dinner**				
Q1	4095	28	1 (Reference)	-
Q2	4150	27	0.93 (0.48-1.78)	0.823
Q3	4061	32	1.26 (0.58-2.76)	0.552
Q4	4118	41	1.68 (0.89-3.16)	0.108
Q5	4024	42	1.45 (0.75-2.8)	0.263
**Age>60**				
**Daily MUFA Intake**				
Q1	1930	183	1 (Reference)	-
Q2	1894	197	1.22 (0.94-1.58)	0.131

Q3	1887	204	1.14 (0.9-1.43)	0.268
Q4	1957	208	1.1 (0.85-1.44)	0.466
Q5	2020	207	1.03 (0.79-1.35)	0.808
**MUFA Intake at Breakfast**				
Q1	1412	121	1 (Reference)	-
Q2	2130	222	1.19 (0.91-1.55)	0.198
Q3	2113	241	1.45 (1.09-1.92)	0.010
Q4	2071	211	1.21 (0.93-1.58)	0.158
Q5	1962	204	1.33 (1-1.77)	0.052
**MUFA Intake at Lunch**				
Q1	2204	231	1 (Reference)	-
Q2	2029	200	1.27 (0.93-1.74)	0.133
Q3	1896	190	1.14 (0.83-1.58)	0.420
Q4	1895	197	1.21 (0.88-1.66)	0.246
Q5	1664	181	1.25 (0.93-1.68)	0.143
**MUFA Intake at Dinner**				
Q1	1934	168	1 (Reference)	-
Q2	1878	188	1.08 (0.84-1.39)	0.534
Q3	1964	213	1.25 (1-1.56)	0.052
Q4	1811	215	1.21 (0.93-1.57)	0.150
Q5	2001	215	1.02 (0.79-1.31)	0.888
		**Hypertension Mortaility**		
		**Case**	**HR (95% CI)**	**P value**
**Age≤60**				
**Daily TUFA Intake**				
Q1	4154	24	1 (Reference)	-
Q2	4146	18	1.01 (0.42-2.4)	0.984
Q3	4138	14	0.46 (0.21-1.04)	0.061
Q4	4084	20	0.87 (0.36-2.14)	0.763
Q5	3926	20	1 (0.48-2.1)	0.998
**TUFA Intake at Breakfast**				
Q1	4691	26	1 (Reference)	-
Q2	3905	11	0.38 (0.16-0.91)	0.031
Q3	3920	21	0.99 (0.46-2.12)	0.974
Q4	3914	18	0.97 (0.38-2.47)	0.943
Q5	4018	20	0.65 (0.31-1.38)	0.259
**TUFA Intake at Lunch**				
Q1	3863	18	1 (Reference)	-
Q2	3974	21	1.4 (0.5-3.9)	0.522
Q3	4115	14	0.55 (0.18-1.73)	0.304
Q4	4191	25	1 (0.38-2.61)	0.994
Q5	4305	18	0.79 (0.25-2.47)	0.678
**TUFA Intake at Dinner**				
Q1	4123	19	1 (Reference)	-

Q2	4184	21	1.04 (0.51-2.11)	0.908
Q3	4097	16	1.02 (0.49-2.1)	0.965
Q4	4032	14	0.76 (0.32-1.79)	0.526
Q5	4012	26	1.53 (0.77-3.03)	0.218
**Age>60**				
**Daily TUFA Intake**				
Q1	1873	123	1 (Reference)	-
Q2	1885	127	1.12 (0.8-1.59)	0.503
Q3	1887	129	1.03 (0.78-1.36)	0.831
Q4	1943	108	0.76 (0.53-1.08)	0.124
Q5	2100	119	1.09 (0.78-1.52)	0.603
**TUFA Intake at Breakfast**				
Q1	1337	70	1 (Reference)	-
Q2	2122	145	1.05 (0.73-1.52)	0.777
Q3	2108	141	1.27 (0.84-1.92)	0.259
Q4	2113	128	1.03 (0.69-1.53)	0.899
Q5	2008	122	1.12 (0.71-1.75)	0.633
**TUFA Intake at Lunch**				
Q1	2164	127	1 (Reference)	-
Q2	2055	138	1.36 (0.95-1.95)	0.089
Q3	1911	120	1.38 (0.96-1.99)	0.081
Q4	1837	121	1.38 (0.99-1.94)	0.056
Q5	1721	100	1.22 (0.8-1.88)	0.353
**TUFA Intake at Dinner**				
Q1	1904	113	1 (Reference)	-
Q2	1844	126	1.17 (0.8-1.71)	0.410
Q3	1931	119	0.91 (0.65-1.28)	0.600
Q4	1994	120	0.99 (0.72-1.37)	0.961
Q5	2015	128	1.04 (0.72-1.5)	0.838
**Age≤60**				
**Daily PUFA Intake**				
Q1	4213	23	1 (Reference)	-
Q2	4161	24	1.31 (0.57-2.99)	0.518
Q3	4073	21	1.1 (0.49-2.49)	0.821
Q4	4036	11	0.53 (0.21-1.36)	0.184
Q5	3965	17	1.26 (0.56-2.84)	0.573
**PUFA Intake at Breakfast**				
Q1	4796	23	1 (Reference)	-
Q2	3961	14	0.58 (0.21-1.6)	0.290
Q3	3888	25	1.63 (0.76-3.49)	0.203
Q4	3894	20	1.08 (0.4-2.92)	0.873
Q5	3909	14	0.67 (0.28-1.59)	0.361
**PUFA Intake at Lunch**				
Q1	3858	19	1 (Reference)	-

Q2	4028	18	0.69 (0.21-2.28)	0.544
Q3	4134	21	0.67 (0.2-2.2)	0.507
Q4	4214	22	0.63 (0.2-2)	0.432
Q5	4214	16	0.53 (0.15-1.92)	0.331
**PUFA Intake at Dinner**				
Q1	4140	24	1 (Reference)	-
Q2	4213	20	0.94 (0.45-1.97)	0.869
Q3	4117	14	0.8 (0.34-1.85)	0.597
Q4	4057	16	0.71 (0.37-1.39)	0.316
Q5	3921	22	1.3 (0.66-2.58)	0.445
**Age>60**				
**Daily PUFA Intake**				
Q1	1813	127	1 (Reference)	-
Q2	1874	127	0.89 (0.67-1.19)	0.443
Q3	1952	123	0.69 (0.52-0.92)	0.012
Q4	1990	124	0.89 (0.67-1.17)	0.398
Q5	2059	105	0.84 (0.63-1.12)	0.227
**PUFA Intake at Breakfast**				
Q1	1236	67	1 (Reference)	-
Q2	2068	143	0.86 (0.63-1.19)	0.359
Q3	2138	149	1.06 (0.74-1.53)	0.741
Q4	2131	130	0.86 (0.63-1.19)	0.356
Q5	2115	117	0.85 (0.55-1.32)	0.471
**PUFA Intake at Lunch**				
Q1	2167	129	1 (Reference)	-
Q2	2000	144	1.27 (0.9-1.79)	0.178
Q3	1897	122	1.14 (0.81-1.61)	0.445
Q4	1810	105	1.16 (0.8-1.7)	0.424
Q5	1814	106	1.21 (0.85-1.72)	0.286
**PUFA Intake at Dinner**				
Q1	1890	127	1 (Reference)	-
Q2	1815	113	0.97 (0.69-1.36)	0.852
Q3	1910	121	1 (0.68-1.49)	0.983
Q4	1972	125	0.87 (0.62-1.23)	0.419
Q5	2101	120	0.88 (0.62-1.25)	0.477
**Age≤60**				
**Daily MUFA Intake**				
Q1	4096	22	1 (Reference)	-
Q2	4134	16	0.69 (0.28-1.69)	0.416
Q3	4140	12	0.93 (0.37-2.34)	0.879
Q4	4072	24	1.16 (0.51-2.61)	0.719
Q5	4006	22	0.73 (0.33-1.63)	0.439
**MUFA Intake at Breakfast**				

Q1	4616	28	1 (Reference)	-
Q2	3901	9	0.25 (0.11-0.58)	0.002
Q3	3914	17	0.66 (0.3-1.42)	0.282
Q4	3955	19	0.93 (0.38-2.23)	0.862
Q5	4062	23	0.7 (0.34-1.44)	0.327
**MUFA Intake at Lunch**				
Q1	3822	19	1 (Reference)	-
Q2	3999	19	0.76 (0.27-2.17)	0.608
Q3	4133	13	0.46 (0.16-1.33)	0.151
Q4	4133	24	0.72 (0.29-1.81)	0.486
Q5	4361	21	0.64 (0.22-1.85)	0.404
**MUFA Intake at Dinner**				
Q1	4095	21	1 (Reference)	-
Q2	4150	16	0.84 (0.4-1.76)	0.637
Q3	4061	15	0.75 (0.38-1.47)	0.394
Q4	4118	19	0.84 (0.4-1.76)	0.643
Q5	4024	25	1.49 (0.76-2.89)	0.241
**Age>60**				
**Daily MUFA Intake**				
Q1	1930	113	1 (Reference)	-
Q2	1894	126	1.28 (0.96-1.7)	0.093
Q3	1887	123	1.22 (0.88-1.69)	0.235
Q4	1957	128	1.14 (0.8-1.64)	0.460
Q5	2020	116	1.02 (0.76-1.38)	0.890
**MUFA Intake at Breakfast**				
Q1	1412	68	1 (Reference)	-
Q2	2130	144	1.21 (0.84-1.74)	0.311
Q3	2113	146	1.42 (0.92-2.18)	0.113
Q4	2071	127	1.28 (0.82-1.98)	0.272
Q5	1962	121	1.22 (0.78-1.89)	0.379
**MUFA Intake at Lunch**				
Q1	2204	128	1 (Reference)	-
Q2	2029	128	1.42 (0.98-2.05)	0.063
Q3	1896	127	1.5 (1.02-2.19)	0.038
Q4	1895	117	1.37 (0.94-2)	0.099
Q5	1664	106	1.33 (0.91-1.95)	0.143
**MUFA Intake at Dinner**				
Q1	1934	125	1 (Reference)	-
Q2	1878	113	0.85 (0.6-1.21)	0.370
Q3	1964	104	0.85 (0.58-1.24)	0.398
Q4	1811	133	0.93 (0.7-1.22)	0.578
Q5	2001	131	0.94 (0.65-1.36)	0.738

Supplementary Table S10. Adjusted hazard ratios for mortalities across quintiles of dietary UFAs intake in all-day and three meals excluded participants with follow-up less than 2 years.

Variable	N	All-cause Mortaility			CVD Mortaility			Heart Mortaility			Hypertension Mortaility		
		Case	HR (95% CI)	P value	Case	HR (95% CI)	P value	Case	HR (95% CI)	P value	Case	HR (95% CI)	P value
**Daily TUFA Intake**													
Q1	5897	787	1 (Reference)	-	300	1 (Reference)	-	185	1 (Reference)	-	127	1 (Reference)	-
Q2	5908	770	0.93 (0.81-1.05)	0.236	335	1.14 (0.93-1.4)	0.202	219	1.18 (0.92-1.52)	0.198	128	1.08 (0.76-1.53)	0.668
Q3	5901	761	0.88 (0.75-1.02)	0.082	315	1.06 (0.86-1.31)	0.559	187	1.07 (0.83-1.37)	0.598	130	0.95 (0.69-1.3)	0.728
Q4	5909	759	0.81 (0.7-0.94)	0.007	286	0.92 (0.72-1.16)	0.469	195	0.98 (0.74-1.31)	0.911	109	0.8 (0.53-1.2)	0.281
Q5	5917	829	0.81 (0.7-0.94)	0.005	336	1.06 (0.84-1.34)	0.609	225	1.08 (0.83-1.42)	0.552	125	1.08 (0.76-1.54)	0.669
per SD			0.92 (0.88-0.97)	0.002		0.99 (0.92-1.06)	0.730		1.01 (0.93-1.1)	0.807		0.96 (0.86-1.07)	0.443
P for linear				0.003			0.746			0.923			0.866
P for nonlinear				0.658			0.197			0.219			0.379
**TUFA Intake at Breakfast**													
Q1	5946	538	1 (Reference)	-	211	1 (Reference)	-	125	1 (Reference)	-	85	1 (Reference)	-
Q2	5912	848	1.26 (1.07-1.49)	0.006	346	1.25 (0.98-1.59)	0.075	228	1.53 (1.15-2.05)	0.005	140	0.99 (0.72-1.38)	0.970
Q3	5892	881	1.38 (1.17-1.64)	<0.001	353	1.39 (1.06-1.81)	0.019	223	1.57 (1.17-2.09)	0.003	142	1.25 (0.84-1.86)	0.275
Q4	5888	859	1.3 (1.08-1.57)	0.006	332	1.24 (0.93-1.66)	0.148	225	1.43 (1.06-1.91)	0.018	129	1.01 (0.64-1.59)	0.960
Q5	5894	780	1.2 (1-1.44)	0.052	330	1.22 (0.91-1.64)	0.177	210	1.32 (0.94-1.85)	0.105	123	0.97 (0.64-1.46)	0.887
per SD			1.03 (0.97-1.08)	0.274		1.02 (0.95-1.11)	0.561		1.02 (0.92-1.12)	0.751		0.97 (0.85-1.1)	0.579
P for linear				0.084			0.293			0.342			0.949
P for nonlinear				0.255			0.134			0.211			0.37
**TUFA Intake at Lunch**													
Q1	5871	875	1 (Reference)	-	339	1 (Reference)	-	220	1 (Reference)	-	121	1 (Reference)	-
Q2	5887	803	1.15 (0.97-1.36)	0.110	330	1.27 (0.97-1.67)	0.087	201	1.26 (0.87-1.83)	0.221	146	1.49 (0.99-2.26)	0.059
Q3	5920	774	1.08 (0.94-1.25)	0.283	313	1.25 (0.97-1.6)	0.083	191	1.29 (0.95-1.77)	0.103	117	1.25 (0.85-1.82)	0.251
Q4	5920	757	1 (0.86-1.16)	0.951	308	1.1 (0.86-1.39)	0.450	210	1.22 (0.88-1.68)	0.223	129	1.27 (0.89-1.82)	0.181
Q5	5934	697	1.01 (0.87-1.17)	0.910	282	1.12 (0.89-1.41)	0.328	189	1.27 (0.95-1.69)	0.105	106	1.1 (0.69-1.74)	0.698
per SD			0.95 (0.91-0.99)	0.045		0.97 (0.92-1.03)	0.311		1.02 (0.96-1.09)	0.474		0.96 (0.86-1.07)	0.443

P for linear				0.217			0.87			0.188			0.673
P for nonlinear				0.277			0.153			0.063			0.593
**TUFA Intake at Dinner**													
Q1	5900	725	1 (Reference)	-	288	1 (Reference)	-	180	1 (Reference)	-	116	1 (Reference)	-
Q2	5910	728	0.99 (0.84-1.17)	0.923	295	1.03 (0.81-1.31)	0.801	177	0.96 (0.72-1.27)	0.754	128	1.13 (0.77-1.66)	0.529
Q3	5899	795	0.94 (0.82-1.09)	0.421	314	0.98 (0.8-1.2)	0.842	219	1.1 (0.85-1.43)	0.478	122	0.92 (0.65-1.31)	0.638
Q4	5916	792	0.94 (0.82-1.07)	0.351	329	1.07 (0.87-1.32)	0.524	226	1.21 (0.92-1.6)	0.166	115	0.88 (0.61-1.27)	0.488
Q5	5907	866	0.93 (0.81-1.06)	0.269	346	1.07 (0.85-1.33)	0.567	209	0.97 (0.72-1.29)	0.822	138	1.2 (0.84-1.71)	0.305
per SD			0.95 (0.91-0.99)	0.034		0.99 (0.93-1.05)	0.707		0.97 (0.9-1.04)	0.348		1 (0.91-1.11)	0.986
P for linear				0.176			0.484			0.665			0.651
P for nonlinear				0.267			0.054			0.047			0.435
**Daily PUFA Intake**													
Q1	5880	845	1 (Reference)	-	336	1 (Reference)	-	217	1 (Reference)	-	134	1 (Reference)	-
Q2	5905	780	0.95 (0.81-1.11)	0.511	332	1.04 (0.83-1.29)	0.740	206	1.03 (0.78-1.36)	0.843	132	0.99 (0.71-1.37)	0.938
Q3	5917	788	0.88 (0.76-1.03)	0.103	313	0.89 (0.72-1.1)	0.271	199	0.97 (0.77-1.21)	0.767	126	0.77 (0.56-1.07)	0.124
Q4	5908	762	0.83 (0.71-0.96)	0.014	303	0.92 (0.75-1.14)	0.460	195	0.92 (0.71-1.19)	0.517	118	0.86 (0.63-1.18)	0.358
Q5	5922	731	0.79 (0.67-0.93)	0.005	288	0.94 (0.74-1.21)	0.639	194	1 (0.73-1.36)	0.998	109	0.97 (0.68-1.37)	0.848
per SD			0.92 (0.88-0.98)	0.005		0.96 (0.89-1.05)	0.375		0.99 (0.89-1.1)	0.822		0.95 (0.85-1.06)	0.346
P for linear				0.001			0.424			0.763			0.658
P for nonlinear				0.463			0.683			0.993			0.563
**PUFA Intake at Breakfast**													
Q1	5956	526	1 (Reference)	-	195	1 (Reference)	-	116	1 (Reference)	-	80	1 (Reference)	-
Q2	5912	843	1.22 (1.05-1.42)	0.012	349	1.34 (1.1-1.63)	0.005	221	1.53 (1.15-2.05)	0.004	146	1.03 (0.76-1.4)	0.862
Q3	5879	904	1.36 (1.15-1.6)	0.000	368	1.44 (1.13-1.83)	0.003	239	1.61 (1.19-2.17)	0.002	146	1.22 (0.84-1.76)	0.288
Q4	5892	851	1.22 (1.02-1.45)	0.027	329	1.29 (1-1.65)	0.046	212	1.38 (1.02-1.88)	0.040	131	0.99 (0.67-1.46)	0.967
Q5	5893	782	1.11 (0.92-1.34)	0.256	331	1.22 (0.95-1.58)	0.123	223	1.41 (1.02-1.97)	0.040	116	0.89 (0.6-1.3)	0.538
per SD			1 (0.95-1.06)	0.929		1 (0.93-1.08)	0.911		1.03 (0.94-1.12)	0.596		0.95 (0.84-1.08)	0.417
P for linear				0.450			0.414			0.270			0.517
P for nonlinear				0.987			0.196			0.272			0.739

**PUFA Intake at Lunch**													
Q1	5860	903	1 (Reference)	-	350	1 (Reference)	-	230	1 (Reference)	-	126	1 (Reference)	-
Q2	5898	831	1.1 (0.93-1.31)	0.266	339	1.15 (0.88-1.51)	0.299	205	1.12 (0.8-1.55)	0.505	146	1.16 (0.76-1.76)	0.500
Q3	5917	761	1.02 (0.87-1.19)	0.830	310	1 (0.79-1.28)	0.979	196	1.05 (0.77-1.44)	0.747	125	0.99 (0.63-1.56)	0.970
Q4	5922	728	0.98 (0.84-1.13)	0.757	292	1.02 (0.78-1.32)	0.897	196	1.13 (0.84-1.51)	0.409	114	0.99 (0.64-1.55)	0.978
Q5	5935	683	0.92 (0.8-1.07)	0.298	281	0.99 (0.79-1.24)	0.943	184	1.09 (0.82-1.45)	0.551	108	0.96 (0.59-1.55)	0.855
per SD			0.96 (0.91-1.01)	0.098		0.95 (0.9-1.01)	0.092		0.99 (0.93-1.06)	0.797		0.94 (0.83-1.05)	0.254
P for linear				0.046			0.404			0.577			0.531
P for nonlinear				0.789			0.262			0.099			0.725
**PUFA Intake at Dinner**													
Q1	5896	781	1 (Reference)	-	318	1 (Reference)	-	191	1 (Reference)	-	135	1 (Reference)	-
Q2	5906	729	0.98 (0.84-1.14)	0.784	298	0.91 (0.73-1.14)	0.415	204	1.05 (0.78-1.41)	0.768	116	0.9 (0.66-1.23)	0.492
Q3	5905	761	0.91 (0.8-1.04)	0.149	294	0.89 (0.72-1.1)	0.287	189	0.92 (0.7-1.22)	0.566	118	0.84 (0.58-1.2)	0.337
Q4	5912	804	0.97 (0.85-1.11)	0.705	328	1.06 (0.86-1.31)	0.561	221	1.26 (0.95-1.67)	0.106	124	0.87 (0.63-1.2)	0.386
Q5	5913	831	0.87 (0.77-0.99)	0.042	334	0.93 (0.75-1.17)	0.549	206	0.91 (0.68-1.21)	0.499	126	0.96 (0.68-1.35)	0.812
per SD			0.94 (0.91-0.98)	0.010		0.97 (0.91-1.04)	0.414		0.95 (0.88-1.02)	0.161		0.99 (0.89-1.09)	0.797
P for linear				0.048			0.936			0.942			0.856
P for nonlinear				0.340			0.330			0.220			0.955
**Daily MUFA Intake**													
Q1	5902	741	1 (Reference)	-	282	1 (Reference)	-	169	1 (Reference)	-	117	1 (Reference)	-
Q2	5909	756	1.04 (0.89-1.21)	0.642	306	1.18 (0.93-1.5)	0.161	202	1.38 (1.03-1.85)	0.030	121	1.07 (0.77-1.48)	0.684
Q3	5908	748	0.96 (0.83-1.12)	0.619	317	1.23 (0.97-1.55)	0.083	203	1.26 (0.96-1.65)	0.094	126	1.21 (0.84-1.75)	0.296
Q4	5894	796	0.94 (0.82-1.08)	0.374	324	1.12 (0.9-1.41)	0.305	206	1.16 (0.9-1.5)	0.250	127	1.07 (0.72-1.6)	0.735
Q5	5919	865	0.87 (0.76-1)	0.043	343	1.06 (0.86-1.31)	0.563	231	1.18 (0.93-1.49)	0.161	128	0.96 (0.69-1.34)	0.808
per SD			0.94 (0.9-0.99)	0.020		1.01 (0.95-1.08)	0.703		1.03 (0.95-1.11)	0.483		0.98 (0.89-1.09)	0.700
P for linear				0.019			0.962			0.793			0.745
P for nonlinear				0.552			0.412			0.351			0.685
**MUFA Intake at Breakfast**													
Q1	5943	560	1 (Reference)	-	223	1 (Reference)	-	139	1 (Reference)	-	85	1 (Reference)	-

Q2	5908	842	1.21 (1.04-1.42)	0.017	329	1.13 (0.88-1.44)	0.346	211	1.25 (0.94-1.66)	0.127	136	0.96 (0.67-1.37)	0.827
Q3	5905	874	1.29 (1.1-1.51)	0.002	366	1.3 (1-1.69)	0.050	237	1.39 (1.07-1.82)	0.016	144	1.22 (0.77-1.92)	0.401
Q4	5885	829	1.28 (1.08-1.51)	0.005	325	1.26 (0.95-1.67)	0.110	218	1.37 (1.02-1.83)	0.036	129	1.15 (0.72-1.84)	0.558
Q5	5891	801	1.23 (1.03-1.46)	0.020	329	1.16 (0.86-1.55)	0.326	206	1.11 (0.82-1.5)	0.496	125	0.98 (0.63-1.52)	0.925
per SD			1.04 (0.99-1.09)	0.102		1.03 (0.95-1.12)	0.433		1.01 (0.92-1.11)	0.866		0.98 (0.86-1.11)	0.718
P for linear				0.027			0.225			0.419			0.735
P for nonlinear				0.093			0.183			0.388			0.271
**MUFA Intake at Lunch**													
Q1	5873	878	1 (Reference)	-	342	1 (Reference)	-	223	1 (Reference)	-	122	1 (Reference)	-
Q2	5898	772	1.1 (0.94-1.3)	0.236	319	1.21 (0.95-1.54)	0.112	193	1.11 (0.87-1.43)	0.401	135	1.35 (0.91-2.01)	0.136
Q3	5909	751	1.07 (0.93-1.23)	0.367	307	1.2 (0.96-1.5)	0.103	190	1.18 (0.91-1.54)	0.213	123	1.26 (0.86-1.85)	0.232
Q4	5921	795	1.07 (0.92-1.24)	0.385	314	1.16 (0.9-1.48)	0.244	210	1.33 (1.02-1.75)	0.039	126	1.2 (0.82-1.76)	0.333
Q5	5931	710	0.96 (0.83-1.11)	0.583	290	1.1 (0.88-1.36)	0.402	195	1 (0.74-1.34)	0.990	113	1.11 (0.73-1.68)	0.614
per SD			0.96 (0.92-1)	0.062		0.99 (0.94-1.05)	0.807		1.05 (0.98-1.11)	0.168		0.98 (0.87-1.1)	0.739
P for linear				0.309			0.809			0.092			0.917
P for nonlinear				0.110			0.207			0.119			0.567
**MUFA Intake at Dinner**													
Q1	5903	718	1 (Reference)	-	286	1 (Reference)	-	172	1 (Reference)	-	127	1 (Reference)	-
Q2	5906	726	1.02 (0.87-1.19)	0.823	287	1.1 (0.91-1.33)	0.339	185	1.11 (0.87-1.43)	0.401	112	0.88 (0.64-1.21)	0.427
Q3	5905	756	0.91 (0.79-1.06)	0.230	302	1.04 (0.84-1.29)	0.721	212	1.18 (0.91-1.54)	0.213	105	0.86 (0.59-1.27)	0.453
Q4	5918	837	1.05 (0.91-1.2)	0.485	341	1.18 (0.96-1.45)	0.114	229	1.33 (1.02-1.75)	0.039	136	0.94 (0.69-1.28)	0.699
Q5	5900	869	0.92 (0.8-1.06)	0.251	356	1.1 (0.89-1.36)	0.353	213	1 (0.74-1.34)	0.990	139	1.11 (0.78-1.56)	0.567
per SD			0.97 (0.93-1.01)	0.153		1 (0.95-1.06)	0.929		0.95 (0.91-1.07)	0.693		1.01 (0.91-1.12)	0.817
P for linear				0.386			0.28			0.697			0.461
P for nonlinear				0.556			0.089			0.135			0.317

Supplementary Table S11. Adjusted hazard ratios for mortalities across quintiles of dietary UFAs intake in all-day and three meals excluded participants self-reported CVD.

Variable	All-cause Mortaility		CVD Mortaility		Heart Mortaility		Hypertension Mortaility	
	HR (95% CI)	P value	HR (95% CI)	P value	HR (95% CI)	P value	HR (95% CI)	P value
**Daily TUFA Intake**								
Q1	1 (Reference)	-	1 (Reference)	-	1 (Reference)	-	1 (Reference)	-
Q2	0.888 (0.766-1.028)	0.110	0.941 (0.735-1.206)	0.629	0.973 (0.717-1.322)	0.861	0.862 (0.582-1.276)	0.454
Q3	0.881 (0.747-1.039)	0.130	0.98 (0.745-1.288)	0.883	1.027 (0.721-1.462)	0.883	0.739 (0.511-1.068)	0.106
Q4	0.821 (0.693-0.973)	0.024	0.877 (0.665-1.155)	0.346	0.812 (0.568-1.16)	0.248	0.842 (0.56-1.266)	0.405
Q5	0.795 (0.687-0.921)	0.002	0.921 (0.711-1.193)	0.528	0.909 (0.652-1.267)	0.570	0.934 (0.636-1.372)	0.726
**TUFA Intake at Breakfast**								
Q1	1 (Reference)	-	1 (Reference)	-	1 (Reference)	-	1 (Reference)	-
Q2	1.251 (1.049-1.493)	0.013	1.305 (0.994-1.713)	0.055	1.276 (0.896-1.816)	0.175	1.175 (0.796-1.736)	0.413
Q3	1.475 (1.222-1.781)	0.000	1.639 (1.212-2.217)	0.002	1.738 (1.237-2.441)	0.002	1.457 (0.948-2.24)	0.086
Q4	1.419 (1.181-1.704)	0.000	1.436 (1.021-2.02)	0.038	1.459 (0.958-2.222)	0.078	1.22 (0.767-1.94)	0.398
Q5	1.267 (1.054-1.523)	0.012	1.338 (0.961-1.865)	0.084	1.391 (0.916-2.11)	0.120	0.904 (0.6-1.363)	0.626
**TUFA Intake at Dinner**								
Q1	1 (Reference)	-	1 (Reference)	-	1 (Reference)	-	1 (Reference)	-
Q2	1.017 (0.869-1.191)	0.828	0.952 (0.768-1.181)	0.654	0.945 (0.679-1.314)	0.734	0.909 (0.61-1.354)	0.637
Q3	1.001 (0.856-1.17)	0.993	0.998 (0.772-1.29)	0.989	1.217 (0.872-1.696)	0.245	0.912 (0.618-1.347)	0.640
Q4	0.98 (0.832-1.154)	0.806	1.082 (0.847-1.383)	0.525	1.185 (0.873-1.609)	0.272	0.895 (0.634-1.264)	0.525
Q5	0.898 (0.762-1.058)	0.196	1.014 (0.793-1.298)	0.908	0.966 (0.688-1.358)	0.842	1.001 (0.694-1.443)	0.996
**Daily PUFA Intake**								
Q1	1 (Reference)	-	1 (Reference)	-	1 (Reference)	-	1 (Reference)	-
Q2	0.912 (0.786-1.058)	0.220	0.819 (0.665-1.009)	0.060	0.803 (0.603-1.071)	0.133	0.738 (0.515-1.058)	0.097
Q3	0.846 (0.714-1.001)	0.051	0.847 (0.643-1.114)	0.232	0.919 (0.679-1.244)	0.580	0.779 (0.537-1.13)	0.185
Q4	0.776 (0.666-0.903)	0.001	0.845 (0.665-1.075)	0.169	0.877 (0.655-1.173)	0.371	0.67 (0.465-0.966)	0.032
Q5	0.736 (0.615-0.882)	0.001	0.826 (0.645-1.06)	0.131	0.819 (0.58-1.156)	0.253	0.855 (0.595-1.23)	0.396
**PUFA Intake at Breakfast**								

Q1	1 (Reference)	-	1 (Reference)	-	1 (Reference)	-	1 (Reference)	-
Q2	1.276 (1.059-1.537)	0.011	1.332 (0.991-1.791)	0.057	1.248 (0.831-1.875)	0.282	1.192 (0.79-1.798)	0.399
Q3	1.451 (1.197-1.759)	0.000	1.673 (1.235-2.266)	0.001	1.693 (1.15-2.492)	0.008	1.638 (1.082-2.482)	0.020
Q4	1.298 (1.066-1.579)	0.010	1.499 (1.044-2.152)	0.029	1.512 (0.955-2.394)	0.077	1.165 (0.746-1.818)	0.498
Q5	1.263 (1.03-1.549)	0.025	1.311 (0.937-1.835)	0.113	1.409 (0.886-2.238)	0.145	0.89 (0.566-1.4)	0.611
**PUFA Intake at Dinner**								
Q1	1 (Reference)	-	1 (Reference)	-	1 (Reference)	-	1 (Reference)	-
Q2	0.915 (0.781-1.071)	0.266	0.88 (0.675-1.148)	0.341	1.01 (0.723-1.41)	0.954	0.899 (0.61-1.325)	0.585
Q3	0.907 (0.773-1.064)	0.227	0.829 (0.634-1.084)	0.168	0.856 (0.621-1.18)	0.339	0.768 (0.496-1.19)	0.234
Q4	0.954 (0.83-1.096)	0.504	1.002 (0.777-1.293)	0.985	1.107 (0.815-1.503)	0.512	0.912 (0.63-1.32)	0.622
Q5	0.807 (0.688-0.947)	0.009	0.87 (0.678-1.114)	0.266	0.888 (0.641-1.229)	0.469	0.897 (0.625-1.288)	0.552
**Daily MUFA Intake**								
Q1	1 (Reference)	-	1 (Reference)	-	1 (Reference)	-	1 (Reference)	-
Q2	1.022 (0.866-1.207)	0.791	1.049 (0.791-1.392)	0.736	1.131 (0.788-1.622)	0.501	0.961 (0.671-1.377)	0.828
Q3	0.905 (0.763-1.073)	0.247	1.048 (0.782-1.404)	0.753	1.043 (0.739-1.472)	0.810	0.984 (0.634-1.528)	0.943
Q4	0.974 (0.836-1.135)	0.735	1.117 (0.875-1.427)	0.371	1.08 (0.788-1.481)	0.627	1.11 (0.76-1.622)	0.585
Q5	0.877 (0.754-1.019)	0.086	0.974 (0.73-1.302)	0.859	0.974 (0.703-1.349)	0.874	0.936 (0.628-1.394)	0.742
**MUFA Intake at Breakfast**								
Q1	1 (Reference)	-	1 (Reference)	-	1 (Reference)	-	1 (Reference)	-
Q2	1.253 (1.051-1.493)	0.012	1.105 (0.815-1.498)	0.515	1.144 (0.784-1.668)	0.482	1.01 (0.664-1.536)	0.964
Q3	1.419 (1.193-1.688)	0.000	1.424 (1.054-1.925)	0.022	1.471 (1.027-2.108)	0.036	1.331 (0.846-2.095)	0.213
Q4	1.406 (1.178-1.679)	0.000	1.31 (0.94-1.826)	0.110	1.411 (0.936-2.126)	0.099	1.193 (0.721-1.974)	0.489
Q5	1.317 (1.095-1.585)	0.004	1.238 (0.876-1.749)	0.223	1.227 (0.826-1.823)	0.307	0.988 (0.636-1.533)	0.956
**MUFA Intake at Dinner**								
Q1	1 (Reference)	-	1 (Reference)	-	1 (Reference)	-	1 (Reference)	-
Q2	0.998 (0.832-1.198)	0.985	0.939 (0.727-1.213)	0.624	0.873 (0.624-1.221)	0.423	0.756 (0.515-1.11)	0.151
Q3	0.927 (0.786-1.093)	0.362	1.093 (0.857-1.394)	0.469	1.323 (0.973-1.8)	0.074	0.805 (0.525-1.234)	0.317
Q4	1.088 (0.923-1.281)	0.313	1.176 (0.907-1.526)	0.219	1.275 (0.906-1.795)	0.162	0.916 (0.67-1.253)	0.580
Q5	0.91 (0.771-1.072)	0.256	1.115 (0.879-1.416)	0.366	0.98 (0.704-1.364)	0.903	1.061 (0.734-1.535)	0.750

Supplementary Table S12. Adjusted hazard ratios for mortalities across quintiles of dietary UFAs intake in all-day and three meals excluded participants with regular night shifts.

Variable	All-cause Mortaility		CVD Mortaility		Heart Mortaility		Hypertension Mortaility	
	HR (95% CI)	P value	HR (95% CI)	P value	HR (95% CI)	P value	HR (95% CI)	P value
**Daily TUFA Intake**								
Q1	1 (Reference)	-	1 (Reference)	-	1 (Reference)	-	1 (Reference)	-
Q2	0.953 (0.853-1.064)	0.384	1.121 (0.915-1.373)	0.266	1.159 (0.911-1.476)	0.227	0.941 (0.672-1.318)	0.721
Q3	0.902 (0.801-1.015)	0.085	0.978 (0.806-1.186)	0.816	0.985 (0.777-1.248)	0.899	0.809 (0.621-1.054)	0.115
Q4	0.853 (0.747-0.975)	0.021	0.883 (0.711-1.097)	0.258	0.934 (0.725-1.202)	0.591	0.75 (0.524-1.074)	0.115
Q5	0.872 (0.766-0.993)	0.039	0.978 (0.803-1.19)	0.820	1.007 (0.781-1.298)	0.958	0.918 (0.679-1.243)	0.577
**TUFA Intake at Breakfast**								
Q1	1 (Reference)	-	1 (Reference)	-	1 (Reference)	-	1 (Reference)	-
Q2	1.208 (1.067-1.367)	0.003	1.136 (0.911-1.418)	0.254	1.235 (0.948-1.609)	0.116	0.958 (0.696-1.319)	0.790
Q3	1.275 (1.122-1.449)	0.000	1.305 (1.076-1.581)	0.007	1.378 (1.091-1.739)	0.008	1.244 (0.886-1.748)	0.205
Q4	1.212 (1.043-1.409)	0.013	1.118 (0.884-1.414)	0.347	1.126 (0.87-1.457)	0.365	1.05 (0.707-1.56)	0.805
Q5	1.2 (1.046-1.377)	0.010	1.194 (0.942-1.513)	0.141	1.213 (0.919-1.601)	0.171	0.925 (0.651-1.314)	0.660
**TUFA Intake at Dinner**								
Q1	1 (Reference)	-	1 (Reference)	-	1 (Reference)	-	1 (Reference)	-
Q2	0.967 (0.85-1.099)	0.602	1.023 (0.833-1.255)	0.828	1.027 (0.781-1.35)	0.847	1.116 (0.794-1.57)	0.523
Q3	0.949 (0.829-1.086)	0.442	0.956 (0.791-1.157)	0.642	1.093 (0.862-1.385)	0.460	0.897 (0.664-1.213)	0.477
Q4	0.925 (0.816-1.05)	0.226	1.02 (0.85-1.224)	0.830	1.122 (0.892-1.412)	0.322	0.962 (0.703-1.317)	0.806
Q5	0.876 (0.772-0.995)	0.042	0.98 (0.809-1.186)	0.832	0.984 (0.757-1.278)	0.901	1.009 (0.75-1.356)	0.954
**Daily PUFA Intake**								
Q1	1 (Reference)	-	1 (Reference)	-	1 (Reference)	-	1 (Reference)	-
Q2	0.946 (0.827-1.082)	0.416	0.986 (0.804-1.21)	0.893	0.97 (0.756-1.244)	0.807	0.999 (0.753-1.325)	0.994
Q3	0.871 (0.764-0.993)	0.039	0.855 (0.691-1.057)	0.145	0.894 (0.702-1.139)	0.359	0.885 (0.646-1.213)	0.443
Q4	0.814 (0.732-0.905)	0.000	0.823 (0.682-0.992)	0.042	0.791 (0.63-0.993)	0.043	0.838 (0.63-1.113)	0.220
Q5	0.791 (0.681-0.917)	0.002	0.844 (0.681-1.046)	0.121	0.876 (0.662-1.161)	0.354	0.857 (0.647-1.135)	0.278
**PUFA Intake at Breakfast**								
Q1	1 (Reference)	-	1 (Reference)	-	1 (Reference)	-	1 (Reference)	-

Q2	1.186 (1.048-1.343)	0.007	1.245 (1.054-1.47)	0.010	1.433 (1.15-1.784)	0.002	1.135 (0.858-1.502)	0.371
Q3	1.298 (1.137-1.482)	0.000	1.391 (1.132-1.71)	0.002	1.463 (1.12-1.91)	0.006	1.479 (1.07-2.043)	0.018
Q4	1.181 (1.035-1.346)	0.014	1.223 (0.984-1.519)	0.069	1.32 (1.022-1.705)	0.034	0.983 (0.708-1.366)	0.919
Q5	1.148 (0.997-1.321)	0.056	1.141 (0.922-1.411)	0.222	1.302 (1.013-1.674)	0.040	0.882 (0.599-1.3)	0.523
**PUFA Intake at Dinner**								
Q1	1 (Reference)	-	1 (Reference)	-	1 (Reference)	-	1 (Reference)	-
Q2	0.933 (0.816-1.067)	0.309	0.992 (0.791-1.243)	0.941	1.139 (0.867-1.495)	0.346	1.024 (0.765-1.371)	0.872
Q3	0.895 (0.785-1.021)	0.098	0.87 (0.719-1.054)	0.154	0.883 (0.695-1.122)	0.304	0.872 (0.637-1.194)	0.389
Q4	0.934 (0.832-1.05)	0.250	1.016 (0.842-1.227)	0.864	1.157 (0.932-1.438)	0.184	0.916 (0.688-1.219)	0.542
Q5	0.812 (0.721-0.916)	0.001	0.857 (0.699-1.049)	0.134	0.872 (0.679-1.121)	0.282	0.891 (0.666-1.192)	0.433
**Daily MUFA Intake**								
Q1	1 (Reference)	-	1 (Reference)	-	1 (Reference)	-	1 (Reference)	-
Q2	1.009 (0.886-1.15)	0.887	1.045 (0.846-1.291)	0.682	1.11 (0.865-1.426)	0.409	0.924 (0.696-1.227)	0.583
Q3	1.001 (0.881-1.137)	0.991	1.179 (0.96-1.447)	0.116	1.234 (0.968-1.574)	0.089	0.985 (0.728-1.334)	0.921
Q4	1.016 (0.899-1.148)	0.798	1.091 (0.9-1.323)	0.369	1.084 (0.853-1.377)	0.508	1.034 (0.756-1.415)	0.832
Q5	0.908 (0.794-1.038)	0.157	0.951 (0.765-1.183)	0.650	1.023 (0.793-1.32)	0.860	0.845 (0.618-1.156)	0.289
**MUFA Intake at Breakfast**								
Q1	1 (Reference)	-	1 (Reference)	-	1 (Reference)	-	1 (Reference)	-
Q2	1.165 (1.038-1.306)	0.010	1.119 (0.912-1.374)	0.278	1.324 (1.058-1.656)	0.015	0.916 (0.654-1.282)	0.604
Q3	1.27 (1.119-1.441)	0.000	1.325 (1.079-1.627)	0.008	1.383 (1.091-1.751)	0.008	1.282 (0.891-1.844)	0.178
Q4	1.256 (1.09-1.448)	0.002	1.145 (0.909-1.441)	0.247	1.278 (0.976-1.673)	0.074	1.039 (0.705-1.531)	0.847
Q5	1.215 (1.061-1.392)	0.005	1.189 (0.941-1.502)	0.144	1.189 (0.926-1.527)	0.172	0.966 (0.659-1.417)	0.860
**MUFA Intake at Dinner**								
Q1	1 (Reference)	-	1 (Reference)	-	1 (Reference)	-	1 (Reference)	-
Q2	1.011 (0.88-1.162)	0.874	1.08 (0.903-1.291)	0.396	1.104 (0.861-1.415)	0.430	0.947 (0.705-1.272)	0.715
Q3	0.905 (0.79-1.037)	0.149	1.04 (0.857-1.264)	0.686	1.199 (0.939-1.53)	0.144	0.818 (0.573-1.166)	0.263
Q4	1.022 (0.898-1.165)	0.736	1.08 (0.894-1.304)	0.423	1.218 (0.946-1.569)	0.125	0.877 (0.68-1.13)	0.307
Q5	0.885 (0.772-1.015)	0.079	1.04 (0.871-1.241)	0.665	1.011 (0.782-1.306)	0.934	0.997 (0.732-1.356)	0.983

Supplementary Table S13. Adjusted hazard ratios for mortalities across quintiles of the difference between total consumption at lunch and dinner and breakfast.

Quintile	All-cause Mortaility		CVD Mortaility		Heart Mortaility		Hypertension Mortaility	
	HR (95% CI)	P value	HR (95% CI)	P value	HR (95% CI)	P value	HR (95% CI)	P value
**ΔTUFA Intake***								
Q1	1 (Reference)	-	1 (Reference)	-	1 (Reference)	-	1 (Reference)	-
Q2	0.998 (0.871-1.144)	0.980	1.036 (0.849-1.265)	0.725	1.099 (0.855-1.413)	0.458	0.982 (0.713-1.298)	0.898
Q3	1.033 (0.912-1.171)	0.608	0.954 (0.778-1.170)	0.650	1.028 (0.818-1.261)	0.810	0.923 (0.679-1.254)	0.604
Q4	0.886 (0.773-1.016)	0.083	0.993 (0.834-1.182)	0.939	1.103 (0.891-1.365)	0.364	0.987 (0.731-1.332)	0.930
Q5	0.807 (0.704-0.925)	0.002	0.903 (0.737-1.105)	0.318	0.905 (0.723-1.132)	0.377	1.043 (0.763-1.425)	0.790
P for linear trend		0.001		0.246		0.371		0.747
**ΔPUFA Intake**								
Q1	1 (Reference)	-	1 (Reference)	-	1 (Reference)	-	1 (Reference)	-
Q2	0.987 (0.858-1.136)	0.856	1.022 (0.817-1.278)	0.847	1.058 (0.821-1.364)	0.659	0.952 (0.682-1.309)	0.759
Q3	0.903 (0.793-1.029)	0.126	0.849 (0.704-1.025)	0.087	0.865 (0.674-1.110)	0.252	0.838 (0.627-1.120)	0.838
Q4	0904 (0.791-1.033)	0.135	0.955 (0.782-1.167)	0.651	0.989 (0.779-1.256)	0.929	0.901 (0.654-1.242)	0.522
Q5	0.788 (0.693-0.895)	<0.001	0.818 (0.680-0.985)	0.034	0.795 (0.635-0.995)	0.045	0.901 (0.675-1.210)	0.485
P for linear trend		<0.001		0.022		0.034		0.426
**ΔMUFA Intake**								
Q1	1 (Reference)	-	1 (Reference)	-	1 (Reference)	-	1 (Reference)	-
Q2	0.977 (0.853-1.119)	0.732	0.902 (0.744-1.095)	0.295	1.020 (0.811-1.282)	0.866	0.813 (0.622-1.0621)	0.127
Q3	1.022 (0.907-1.152)	0.718	0.987 (0.795-1.225)	0.906	1.107 (0.876-1.398)	0.390	0.986 (0.733-1.326)	0.923
Q4	0.904 (0.777-1.052)	0.190	0.985 (0.795-1.221)	0.890	1.113 (0.870-1.425)	0.391	0.980 (0.711-1.351)	0.902
Q5	0.850 (0.743-0.974)	0.020	0.966 (0.801-1.164)	0.711	1.068 (0.858-1.328)	0.553	0.999 (0.724-1.380)	0.996
P for linear trend		0.013		0.900		0.419		0.598

*Δ = UFAs at lunch + dinner - breakfast, %E.

Supplementary Figure S1. Associations of the difference in UFAs intakes between dinner and breakfast with the risk of hypertension mortality.

Hypertension Mortality

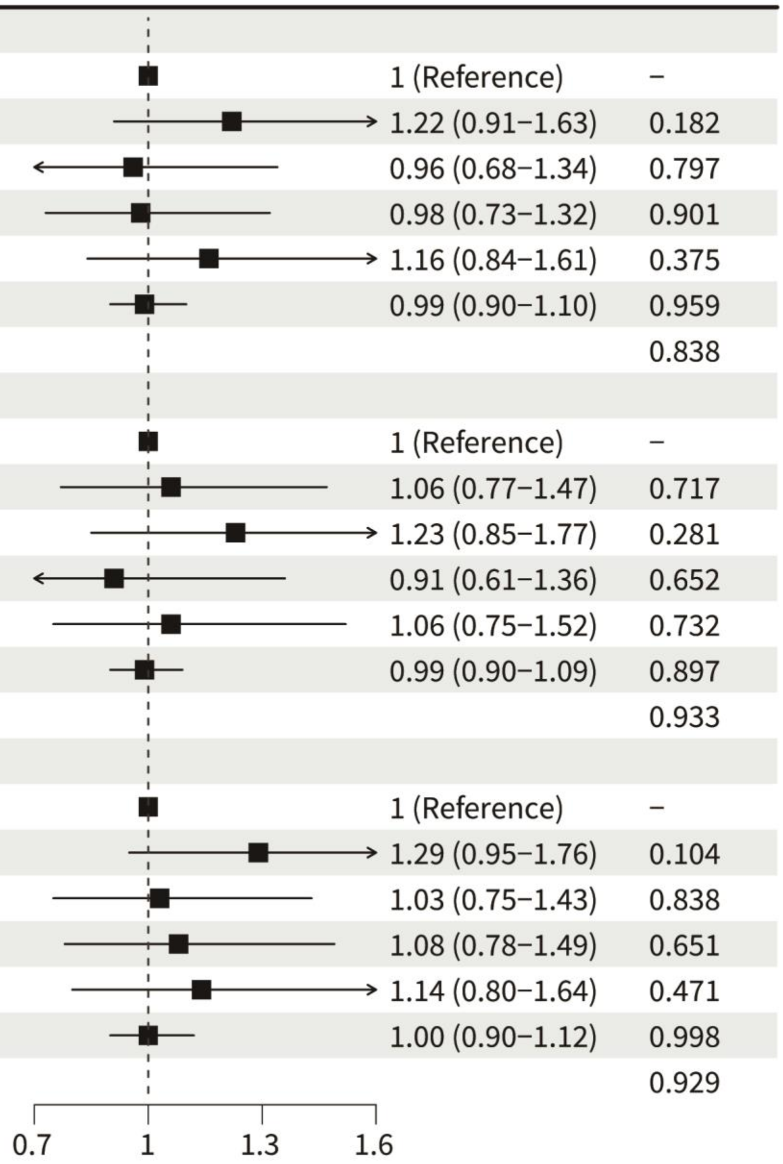

Intake	N	Death	HR (95%CI)	*P* Value
**ΔTUFA**				
Q1	6027	136	1 (Reference)	–
Q2	6027	166	1.22 (0.91–1.63)	0.182
Q3	6026	128	0.96 (0.68–1.34)	0.797
Q4	6030	126	0.98 (0.73–1.32)	0.901
Q5	6026	146	1.16 (0.84–1.61)	0.375
per SD			0.99 (0.90–1.10)	0.959
P for linear				0.838
**ΔPUFA**				
Q1	6028	136	1 (Reference)	–
Q2	6026	154	1.06 (0.77–1.47)	0.717
Q3	6028	157	1.23 (0.85–1.77)	0.281
Q4	6027	116	0.91 (0.61–1.36)	0.652
Q5	6027	139	1.06 (0.75–1.52)	0.732
per SD			0.99 (0.90–1.09)	0.897
P for linear				0.933
**ΔMUFA**				
Q1	6027	134	1 (Reference)	–
Q2	6026	165	1.29 (0.95–1.76)	0.104
Q3	6032	131	1.03 (0.75–1.43)	0.838
Q4	6026	132	1.08 (0.78–1.49)	0.651
Q5	6025	140	1.14 (0.80–1.64)	0.471
per SD			1.00 (0.90–1.12)	0.998
P for linear				0.929

Supplementary Figure S2. Associations of the difference intake levels at breakfast and dinner of UFAs with the risk of hypertension mortality.

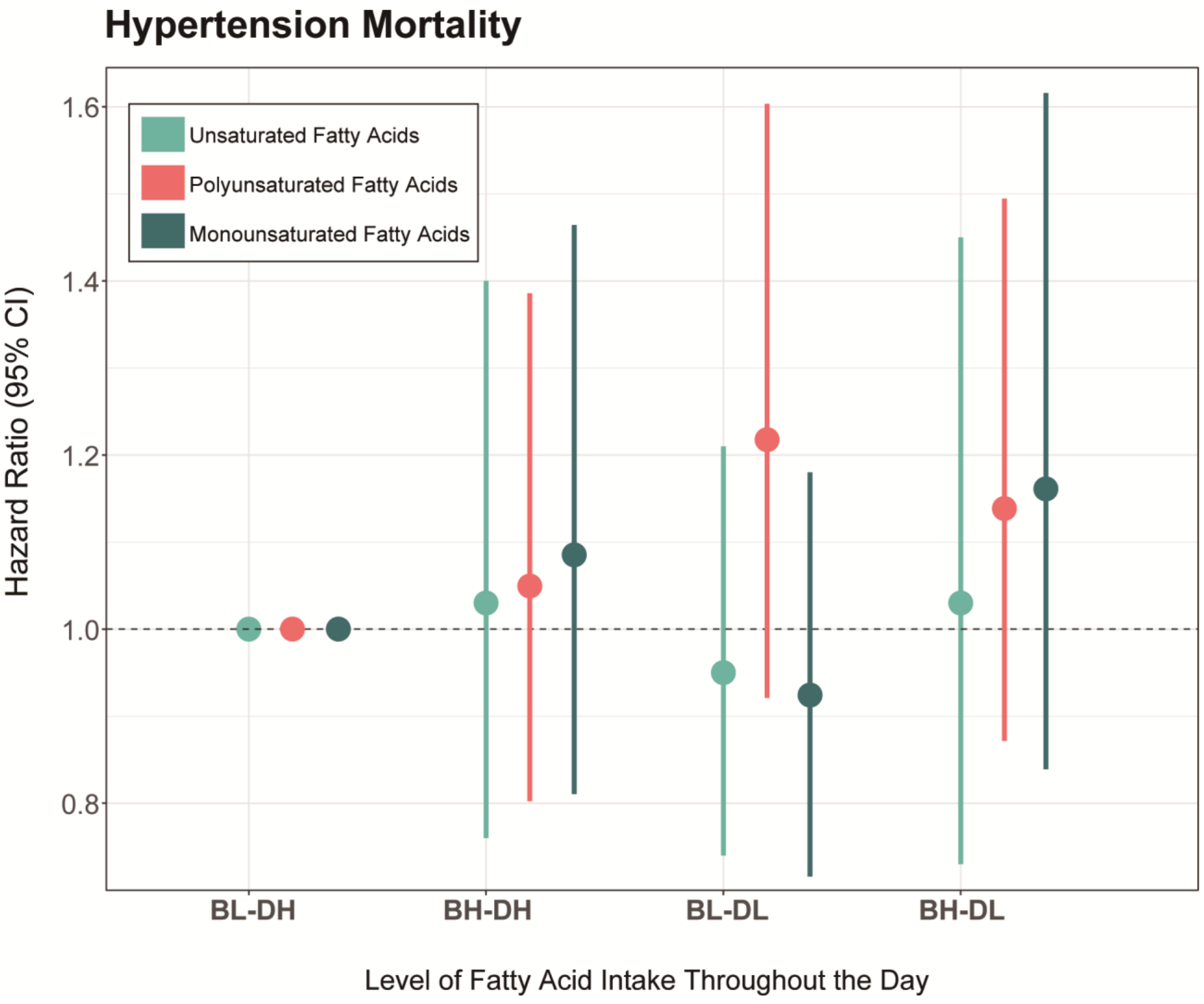

Supplement: Supplementary file 1 [file nutrients-16-02071-s001.zip › nutrients-3073025-supplementary.pdf]
